# Supplementary material for: Biofilm heterogeneity-adaptive photoredox catalysis enables red light-triggered nitric oxide release for combating drug-resistant infections
Source: Nat Commun. 2023 Nov 18;14:7510. doi: 10.1038/s41467-023-43415-8 (PMC10657346; doi:10.1038/s41467-023-43415-8)
Supplement: Supplementary file 1 — Supplementary Information [file 41467_2023_43415_MOESM1_ESM.pdf]

Supplementary Information

Biofilm Heterogeneity-Adaptive Photoredox Catalysis Enables Red Light-Triggered Nitric Oxide Release for Combating Drug-Resistant Infections

Jian Cheng[1], Guihai Gan[2], Shaoqiu Zheng[2], Guoying Zhang[2], Chen Zhu[1], Shiyong Liu[2*] and Jinming Hu[2*]*

[1] *Department of Orthopedics, The First Affiliated Hospital of University of Science and Technology of China (USTC), Division of Life Sciences and Medicine, University of Science and Technology of China, Hefei, Anhui Province, 230001 China.*
[2] *Department of Pharmacy, The First Affiliated Hospital of University of Science and Technology of China (USTC), and Key Laboratory of Precision and Intelligent Chemistry, Department of Polymer Science and Engineering, University of Science and Technology of China, Hefei, Anhui Province, 230026, China.*

* To whom correspondence should be addressed. E-mail: zhuchena@ustc.edu.cn (C.Z.), sliu@ustc.edu.cn (S.L.); jmhu@ustc.edu.cn (J.H.).

Materials

4-Chloronitrobenzene (98%), 4-iodoanisole (98%), ciprofloxacin (Cip, 98%), 5,5-dimethyl-1-pyrroline-1-oxide (DMPO, 98%), dibutyltin dilaurate (DBTL, 98%), 2-(azepan-1-yl)ethan-1-ol (93%), and triethylamine (TEA, 99.5%) were purchased from Energy Chemical (Shanghai, China). 2-Aminoethanol (99%), 1-bromobenzene (99.5%), potassium carbonate (K_2CO_3, 99%), sodium nitrite ($NaNO_2$, 99%), methacryloyl chloride (98%), 2-(diisopropylamino)ethyl methacrylate (DPAM, 98%), 2-(diethylamino)ethyl methacrylate (DEAM, 99%), 2-phenyl-4,4,5,5-tetramethylimidazoline-1-oxyl 3-oxide (PTIO, 98%), sodium ascorbate (SA, 99%), and potassium hydroxide (KOH, 85%) were purchased from Sinopharm Chemical Reagent Co. Ltd (China). Copper(I) chloride (CuCl, 99%), 2-isocyanatoethyl methacrylate (98%), and 3-(4,5-dimethylthiazol-2-yl)-2,5-diphenyltetrazolium bromide (MTT, 98%) were purchased from Sigma-Aldrich (St. Louis, MO, USA). Fluorescein isothiocyanate isomer I (FITC, 95%), coumarin 120 (97.5%), and Nile red (97.5%) were purchased from J&K Scientific Co., Ltd (Beijing, China). 2,2'-Azobis(2-methylpropionitrile) (AIBN, 98%) was purified by recrystallization from 95% ethanol. Singlet oxygen sensor green (SOSG) and LIVE/DEAD® BacLight™ bacterial viability kit reagents were purchased from Thermo Fisher and used as received unless otherwise noted. DPAM and DEAM were purified by distillation. Dichloromethane (DCM) was purified in a Solvent Purification System (Pure Solv™). Other reagents and solvents were used directly unless otherwise stated. Water was deionized on a Milli-Q® direct water purification system to reach a specific resistance of 18.2 MΩ cm. Pyrylium cation NO fluorescence probe (NOFP)[1], RhBM[2], palladium tetraphenyltetrabenzoporphyrin (PdTPTBP)[3], 2-hexamethyleneimino ethyl methacrylate (C7A)[4], and poly(ethylene glycol) (PEG)-based macroRAFT agent[5] were prepared according to previous literature reports. All other reagents were purchased from Sinopharm Chemical Reagent Co., Ltd. and used as received unless otherwise stated. Ciprofloxacin-resistant clinical isolates of *Pseudomonas aeruginosa* (CRPA) were used for antibiofilm and in vivo anti-infectious studies.

Characterization methods

^1H and ^{13}C NMR spectra were taken on a 400 MHz Bruker nuclear magnetic resonance (NMR) spectrometer. Deuterated chloroform ($CDCl_3$) and dimethylsulfoxide (DMSO-d_6) were used as solvents. High-resolution electrospray ionization mass spectrometry (HR-ESI-MS) spectra were performed on Waters XEVO® G2-XS-TOF Mass Spectrometer equipped with an electrospray interface. High-performance liquid chromatography (HPLC) analysis was performed with a Shimadzu HPLC system, equipped with an LC-20AP binary pump, an SPD-20A UV-vis detector, and a Symmetry C18 column. UV-vis absorption spectra were acquired on a UV-3600i Plus UV-vis spectrophotometer (Shimadzu). Molecular weights and molecular weight distributions were determined by size exclusion chromatography (SEC) equipped with Waters 1515 pump and Waters 2414 differential refractive index detector (set at 30 °C). It used a series of two linear Styragel columns (HR2 and HR4) at an oven temperature of 45 °C. The eluent was THF at a flow rate of 1.0 mL/min. Narrowly dispersed polystyrenes were employed as the standards for calibration. Transmission electron microscopy (TEM) was conducted on a JEOL 2010 electron microscope at an

acceleration voltage of 200 kV. Scanning electron microscopy (SEM) was conducted on a Zeiss Gemini 500 field emission scanning electron microscope at an acceleration voltage of 3 kV. Dynamic light scattering (DLS) and zeta potential measurements were conducted on a Zetasizer Nano ZS (Malvern). Electron paramagnetic resonance (EPR) spectra were recorded on a JEOL JES FA200 ESR spectrometer (300 K, 9.063 GHz, Xband) at room temperature. It used the following parameters, microwave power: 1 mW; modulation frequency: 100 kHz; and modulation amplitude: 0.35 mT. Hematoxylin and Eosin (H&E), Masson staining, CD31, and TNF-α staining images were obtained on an IX71 fluorescence microscope (Olympus, Japan). Confocal laser scanning microscopy (CLSM) images were acquired using a Leica TCS SP5 microscope.

Sample synthesis

Synthetic schemes utilized for the preparation of NBNH, NBNO, NBNOM, NBNHM, MBNO, and BNO, as well as the diblock copolymers of **BP1**, **BP2**, **BP3**, **BP4**, **BP5**, **BP6**, **BP7**, and **BP8**, are depicted in Supplementary Figs. 1 and 24.

Synthesis of NBNH (Supplementary Fig. 1)

A mixture comprising 2-aminoethanol (5.8 g, 95.5 mmol) and potassium carbonate (13.2 g, 95.5 mmol) was introduced into a solution containing 4-chloronitrobenzene (3 g, 19.1 mmol) dissolved in DMF (30 mL). The resulting solution was stirred at 90 ^{o}C for 8 h. Upon completion, the mixture was filtered following the removal of DMF through vacuum evaporation. The resulting mixture was then diluted with water (100 mL) and subjected to extraction with DCM. The organic layer was subsequently dried using Na_2SO_4. The filtrate obtained from this process was concentrated under reduced pressure. To obtain the desired product, the mixture was purified via column chromatography on silica gel. The eluent used for this process was a mixture of PE: EA = 1:1 (v/v) (2.4 g, 68.6%). ^{1}H NMR (400 MHz, DMSO-d_6, δ, ppm, TMS, Supplementary Fig. 2):δ 7.98 (d, J = 9.3 Hz, 2H), 7.33 (t, J = 5.7 Hz, 1H), 6.67 (d, J = 9.4 Hz, 2H), 4.84 (t, J = 5.3 Hz, 1H), 3.57 (q, J = 5.6 Hz, 2H), 3.24 (q, J = 5.8 Hz, 2H). ^{13}C NMR (101 MHz, DMSO-d_6, δ, ppm, Supplementary Fig. 2) δ 155.23, 135.93, 126.67, 111.22, 59.71, 45.52. ESI-MS (Supplementary Fig. 2): m/z calc. for $C_8H_{10}N_2O_3$ $[M+Na]^+$: 205.0584, found: 205.0575.

Synthesis of NBNO (Supplementary Fig. 1)

NBNH (300 mg, 1.65 mmol) was dissolved in acetic acid (20 mL), followed by the addition of $NaNO_2$ (230 mg, 3.3 mmol). The resulting mixture was stirred at room temperature for a duration of 6 h. Subsequently, the residue was transferred into a saturated solution of $NaHCO_3$. The aqueous phase was then subjected to extraction with DCM and the combined organic phase was dried using Na_2SO_4. After removing DCM by a rotary evaporator, the residue was purified by column chromatography on silica gel to afford the desired product (312 mg, 89.6%). ^{1}H NMR (400 MHz, chloroform-d, δ, ppm, TMS, Supplementary Fig. 3): δ 8.35 (d, J = 9.2 Hz, 2H), 7.92 (d, J = 9.2 Hz, 2H), 4.22 (t, J = 5.3 Hz, 2H), 3.87 (t, J = 5.3 Hz, 2H). ^{13}C NMR (101 MHz, Chloroform-d, δ, ppm, Supplementary Fig. 3) δ 125.30, 125.11, 119.13, 118.47, 59.12, 46.68. ESI-MS (Supplementary Fig. 3): m/z calc. for $C_8H_9N_3O_4$ $[M+Na]^+$: 234.0485, found: 234.0489.

Synthesis of NBNOM monomer (Supplementary Fig. 1)

NBNO (500 mg, 2.4 mmol) and dibutyltin dilaurate (DBTL, 50 μL) were dissolved in 20 mL of anhydrous DCM. Subsequently, 2-isocyanatoethylmethacrylate (447 mg, 2.88 mmol) dissolved in 2 mL of anhydrous DCM was added dropwise to the reaction mixture. The resulting mixture was then stirred overnight at room temperature. Following this, the mixture was subjected to washing with saturated brine. After drying over Na_2SO_4, the residue was purified by column chromatography on silica gel to yield the desired product (802 mg, 91.3%). ^{1}H NMR (400 MHz, chloroform-*d*, δ, ppm, TMS, Supplementary Fig. 4) δ 8.36 (d, *J* = 9.2 Hz, 2H), 7.84 (d, *J* = 9.2 Hz, 2H), 6.10 (s, 1H), 5.60 (s, 1H), 5.00 (s, 1H), 4.31 (t, *J* = 5.9 Hz, 2H), 4.21 (t, *J* = 5.4 Hz, 4H), 3.47 (q, *J* = 5.5 Hz, 2H), 1.93 (s, 3H). ^{13}C NMR (101 MHz, Chloroform-*d*, δ, ppm, Supplementary Fig. 4) δ 167.31, 155.65, 146.64, 145.97, 135.86, 126.21, 125.29, 118.16, 63.41, 60.19, 42.52, 40.43, 18.28. HPLC analysis (Supplementary Fig. 4): elution peak at 5.3 min (3:2, v/v, MeCN/H_2O; λ = 350 nm). ESI-MS (Supplementary Fig. 4): m/z calc. for $C_{15}H_{18}N_4O_7$ $[M+Na]^+$: 389.1068, found: 389.1061.

Synthesis of NBNHM (Supplementary Fig. 1)

NBNH (500 mg, 2.75 mmol) and dibutyltin dilaurate (DBTL, 50 μL) were dissolved in 20 mL of anhydrous DCM. Subsequently, 2-isocyanatoethylmethacrylate (512 mg, 3.3 mmol) dissolved in 2 mL of anhydrous DCM was added dropwise to the reaction mixture. The resulting mixture was then stirred overnight at room temperature. Afterward, the mixture was subjected to washing with saturated brine. After drying over Na_2SO_4, the residue was purified by column chromatography on silica gel to yield the desired product (830 mg, 89.5%). ^{1}H NMR (400 MHz, chloroform-*d*, δ, ppm, TMS, Supplementary Fig. 5) δ 8.09 (d, *J* = 9.2 Hz, 2H), 6.56 (d, *J* = 9.1 Hz, 2H), 6.12 (s, 1H), 5.60 (s, 1H), 5.10 (t, *J* = 6.2 Hz, 1H), 4.34 (t, *J* = 5.2 Hz, 2H), 4.26 (t, *J* = 5.2 Hz, 3H), 3.52 (q, *J* = 5.5 Hz, 2H), 3.46 (t, *J* = 5.3 Hz, 2H), 1.94 (s, 1H). ^{13}C NMR (101 MHz, chloroform-*d*, δ, ppm, Supplementary Fig. 5) δ 167.38, 156.53, 153.01, 138.23, 135.87, 126.41, 111.10, 63.50, 63.06, 43.20, 40.50, 18.32. HPLC analysis (Supplementary Fig. 5): elution peak at 4.8 min (3:2, v/v, MeCN/H_2O; λ = 350 nm). ESI-MS (Supplementary Fig. 5): m/z calc. for $C_{15}H_{19}N_3O_6$ $[M+Na]^+$: 360.1166, found: 360.1162.

Synthesis of MBNO (Supplementary Fig. 1)

A flask was charged with CuCl (0.126 g, 1.28 mmol), 4-iodoanisole (3 g, 12.8 mmol), 2-aminoethanol (3.9 g, 63.9 mmol), and KOH (1.44 g, 25.6 mmol). The reaction mixture was stirred at room temperature for 8 h. Following this, the resulting mixture was diluted with water, and then subjected to extraction with DCM. The organic phase was dried using Na_2SO_4. The filtrate was concentrated under reduced pressure. To obtain the desired product, the mixture was further purified by column chromatography on silica gel (2.05 g, 95.6%). For the next step, the product (200 mg, 1.2 mmol) was dissolved in acetic acid (15 mL), and $NaNO_2$ (165 mg, 2.38 mmol) was added. The mixture was stirred at room temperature for 6 h. Then, a saturated solution of $NaHCO_3$ was added to the mixture. The aqueous phase was extracted with DCM, and the combined organic phase was dried over Na_2SO_4. After removing the DCM using a rotary evaporator, the residue was purified by column chromatography on silica gel to yield the desired product (220 mg, 93.5%). ^{1}H NMR (400 MHz, chloroform-*d*, δ, ppm, TMS, Supplementary Fig. 6) δ 7.52 (d, *J* = 9.0 Hz, 2H), 6.99 (d, *J* = 9.0 Hz, 2H), 4.18

(t, J = 5.5 Hz, 2H), 3.86 (s, 3H), 3.83 (t, J = 5.5 Hz, 2H). ^{13}C NMR (101 MHz, chloroform-*d*, δ, ppm, Supplementary Fig. 6) δ 159.20, 135.36, 122.46, 114.59, 59.25, 55.59, 48.62. ESI-MS (Supplementary Fig. 6): m/z calc. for $C_9H_{12}N_2O_3$ $[M+Na]^+$: 219.0742, found: 219.0740.

Synthesis of BNO (Supplementary Fig. 1)

A flask was charged with CuCl (0.19 g, 1.92 mmol), 1-bromobenzene (3 g, 19.2 mmol), 2-aminoethanol (5.9 g, 96.6 mmol), and KOH (2.15 g, 38.4 mmol). The reaction mixture was stirred at 90 ^{o}C for 8 h. Following this, the resulting mixture was diluted with water and subjected to extraction with DCM. The organic phase was dried using Na_2SO_4. The filtrate obtained was concentrated under reduced pressure. To obtain the desired product, the mixture was purified by column chromatography on silica gel. The resulting product was obtained (1.93 g, 73.4%). For the next step, the product (200 mg, 1.46 mmol) was dissolved in acetic acid (15 mL), and $NaNO_2$ (165 mg, 2.38 mmol) was added. The mixture was stirred at room temperature for 6 h. Then, a saturated solution of $NaHCO_3$ was added to the mixture. The aqueous phase was extracted with DCM, and the combined organic phase was dried over Na_2SO_4. After removing the DCM using a rotary evaporator, the residue was purified by column chromatography on silica gel, affording the desired product (150 mg, 61.9%). ^{1}H NMR (400 MHz, chloroform-*d*, δ, ppm, TMS, Supplementary Fig. 7) δ 7.62 (d, J = 8.4 Hz, 2H), 7.47 (t, J = 7.9 Hz, 2H), 7.37 (t, J = 7.4 Hz, 1H), 4.20 (t, J = 5.6 Hz, 2H), 3.81 (t, J = 5.6 Hz, 2H), 2.23 (s, 1H). ^{13}C NMR (101 MHz, chloroform-*d*, δ, ppm, Supplementary Fig. 7) δ 142.05, 129.47, 127.65, 120.37, 59.06, 47.90. ESI-MS (Supplementary Fig. 7): m/z calc. for $C_8H_{10}N_2O_2$ $[M+Na]^+$: 189.0634, found: 189.0636.

Synthesis of 2-(hexamethyleneimino) ethyl methacrylate (C7AM)

First, 2-(Hexamethyleneimino) ethanol (1.43 g, 10 mmol), triethylamine (1.21 g, 12 mmol), and inhibitor hydroquinone (0.011 g, 0.1 mmol) were dissolved in 20 mL anhydrous THF. Methacryloyl chloride (1.25 g, 12 mmol) was dissolved in 10 mL THF and added dropwise into the flask. The solution was refluxed in THF for 2 h. After reaction, the solution was filtered to remove the precipitated salts, and THF was removed by rotovap. The resulting residue was distilled in vacuo (80 ~ 90 ^{o}C at 0.05 mm Hg) as a colorless liquid (1.33 g, 63%). ^{1}H NMR (400 MHz, chloroform-*d*, δ, ppm, TMS) δ 6.11 (s, 1H), 5.56 (s, 1H), 4.26 (t, J = 6.2 Hz, 2H), 2.86 (t, J = 6.2 Hz, 2H), 2.78 – 2.65 (m, 4H), 1.95 (s, 3H), 1.62 (d, J = 25.1 Hz, 8H).

Synthesis of palladium tetraphenyltetrabenzoporphyrin (PdTPTBP)

Phenylacetic acid (12.7 g, 93.5 mmol), sodium hydroxide (4 g, 100 mmol) and 350 mL water were mixed together, the pH value was adjusted to 8.0. Then $ZnCl_2$ (7.5 g, 56.1 mmol) was dissolved in water and the pH value was adjusted to 3.0. Zinc phenylacetate was obtained as a white precipitate and dry. The product Zinc phenylacetate (1.22 g, 2.5 mmol), phthalimide (1.47 g, 10.0 mmol) and phenylacetic acid (1.80 g, 13.3 mmol) were ground in a mortar and loaded into a 50 mL round-bottom flask. The solid mixture was split into equal portions, placed into 2.5 mL vials. The vials were heated to a temperature of 280 ^{o}C and stirred for 1 h until the color turned dark green, and subsequently left to cool. The melt was diluted with acetone and the mixture was precipitated into water/ethanol/saturated sodium bicarbonate aqueous solution (v/v/v,

100:20:5). The sediment was purified on column chromatography (Al_2O_3, eluent: DCM) to obtain ZnTPTBP. The product (228 mg, 0.26 mmol) was dissolved in 500 mL of DCM and 0.5 mL of methanesulfonic acid was slowly added and stirred for 24 h. The mixture was extracted with H_2O/sat. $NaHCO_3$ (10/1, v/v) until no protonated ligand could be observed. The product was dried over Na_2SO_4 and the solvent was removed. The sediment was purified on column chromatography (Al_2O_3, eluent: DCM) to obtain H_2TPTBP. The free ligand (164 mg, 0.2 mmol) was dissolved in 200 mL toluene. The solution was heated to reflux at 170 °C. N_2 was bubbled through the reaction mixture. $Pd(C_6H_5CN)_2Cl_2$ (152.2 mg, 0.322 mmol) was added in small pre-dissolved portions over 8 h. After cooling, the solvent removed using rotary evaporation. The crude product was purified on column chromatography (Al_2O_3, eluent: PE/DCM = 2/1).

Synthesis of poly(ethylene glycol) (PEG)-based macroRAFT agent

4-Cyano-4-(((propylthio) carbonothioyl)thio)pentanoic acid (1.38 g, 5 mmol), DCC (1.03 g, 5 mmol) and DMAP (0.06 g, 0.5 mmol) in 40 mL of anhydrous THF were introduced under nitrogen atmosphere in a flask containing 12.5 g of dried MPEO (2.5 mmol). The esterification reaction proceeded under stirring at room temperature for 72 h. The polymer was recovered by precipitation of the mixture in cold diethyl ether. After filtration, the product was dired under vacuum at 40 °C and stored at 5~8 °C.

Synthesis of PEG-*b*-P(NBNO-*co*-Pd) diblock copolymer through reversible addition-fragmentation chain transfer (RAFT) polymerization (Supplementary Fig. 24)

Briefly, NBNOM (55 mg, 0.15 mmol), PdM (29.5 mg, 0.025 mmol), PEG-based macroRAFT agent (26.5 mg, 0.005 mmol), and AIBN (0.16 mg, 0.001 mmol) were dissolved in 150 μL DMSO in a reaction tube equipped with a magnetic stirring bar. The reaction tube was subjected to three cycles of freeze-pump-thaw to degas the contents and then sealed under vacuum. After being thermostated at 70 °C in an oil bath for 8 h, the reaction was quenched by immersion in liquid nitrogen, and the tube was opened. The resulting mixture was precipitated into 50 mL of diethyl ether, and this precipitation process was repeated three times. The sediment was filtered and dried under vacuum overnight, PEG-*b*-P(NBNO-*co*-Pd) (**BP1**) diblock copolymer was obtained as a green solid. The resulting diblock copolymer was denoted as PEG_{133}-*b*-$P(NBNO_{0.88}$-*co*-$Pd_{0.12})_{21}$ by ^1H NMR analysis (Supplementary Fig. 25; Abbreviated as **BP1**). The number average molecular weight (M_n) of **BP1** diblock copolymer was 15.0 kDa and the molecular weight distribution (M_w/M_n) was determined to be 1.06 (Supplementary Table 2).

The synthesis of **BP2** diblock copolymers, with the substitution of NBNOM for NBNHM (50.6 mg, 0.15 mmol), and the synthesis of **BP6** diblock copolymers, with the replacement of PdM with RhBM (13.9 mg, 0.025 mmol), followed similar protocols. The structural parameters of these copolymers are summarized in Supplementary Table 2.

Synthesis of PEG-*b*-P(C7A-*co*-Pd) diblock copolymer through RAFT polymerization (Supplementary Fig. 24)

Briefly, C7AM (53 mg, 0.25 mmol), PdM (29.5 mg, 0.025 mmol), PEG-based macroRAFT agent (26.5 mg, 0.005 mmol), and AIBN (0.16 mg, 0.001 mmol) were

dissolved in 150 μL DMSO in a reaction tube equipped with a magnetic stirring bar. The reaction tube was subjected to three cycles of freeze-pump-thaw to degas the contents and then sealed under vacuum. After being thermostated at 70 °C in an oil bath for 8 h, the reaction was quenched into liquid nitrogen and opened. The reaction mixture was diluted with THF (2 mL). The residue obtained was subsequently dialyzed in distilled water and subjected to lyophilization, resulting in the formation of a green powder. The resulting diblock copolymer was denoted as PEG_{133}-*b*-$P(C7A_{0.95}$-*co*-$Pd_{0.05})_{47}$ by ^{1}H NMR analysis (Supplementary Fig. 26; Abbreviated as **BP3).** The number average molecular weight (M_n) of **BP3** diblock copolymer was 18.4 kDa and the molecular weight distribution (M_w/M_n) was determined to be 1.10 (Supplementary Table 2).

The synthesis of **BP4** (substituting C7AM with DPAM, 53.3 mg, 0.25 mmol), **BP5** (substituting C7AM with DEAM, 46.3 mg, 0.25 mmol), **BP7** (without adding PdM), and **BP8** (substituting PdM with RhBM, 13.9 mg, 0.025 mmol) diblock copolymers followed similar protocols, and the structural parameters of these copolymers are summarized in Supplementary Table 2.

Synthesis of nitric oxide fluorescent probe (NOFP)

$BF_3 \cdot OEt_2$ (3.0 mL, 24 mmol) were added to a solution of 4-hydroxy-3-nitrobenzaldehyde (2 g, 12 mmol) and 4-methoxyacetophenone (3.6 g, 24 mmol) in anhydrous toluene (20 mL). The solution was refluxed for 2 h。 After cooling to room temperature, acetone (20 mL) was added and the solution was poured into excess ether (400 mL). The precipitate was formed which was then filtered, washed with ether and dried under vacuum. $SnCl_2 \cdot 2H_2O$ (0.81 g, 3.48 mmol) was added to a solution of the product (0.3 g, 0.58 mmol) in 30 mL DCM and excess of hydrochloric acid (0.53 mL, 17.4 mmol) under N_2 atmosphere. The reaction mixture was refluxed for 3 h. After this time, the reaction mixture was neutralized with sodium hydroxide and extracted with DCM. Finally, the organic phase were collected, dried with Na2SO4 and the solvent was removed. Apurple powder was obtained.(231 mg, 81%). ^{1}H NMR (400 MHz, DMSO-d_6, δ, ppm, TMS) δ 8.18 (s, 1H), 8.08 (d, J = 6.3 Hz, 4H), 7.96 (d, J = 9.6 Hz, 1H), 7.14 (d, J = 8.8 Hz, 4H), 7.11 (s, 2H), 6.24 (d, J = 9.3 Hz, 1H), 4.77 (s, 2H), 3.87 (s, 6H).

Red light-mediated NO release and Quantification of NO release

To investigate the NO release of NBNO in the presence of PdTPTBP photocatalyst, the mixture of these compounds was dissolved in either DMSO or DMF. Subsequently, the mixture was exposed to irradiation using an LED lamp. The changes in the UV-vis spectra were monitored and recorded during the process. The NO release amounts were calculated using the established protocol using the following equations:

$$A_{\lambda_1} = c_0 b\left(\varepsilon_{\lambda_1}^{NO} x_{NO} + \varepsilon_{\lambda_1}^{NH} x_{NH}\right)$$

$$A_{\lambda_2} = c_0 b\left(\varepsilon_{\lambda_2}^{NO} x_{NO} + \varepsilon_{\lambda_2}^{NH} x_{NH}\right)$$

$$x_{NO} = \frac{1}{c_0 b} \bullet \left[\frac{A_{\lambda_1} \bullet \varepsilon_{\lambda_2}^{NH} - A_{\lambda_2} \bullet \varepsilon_{\lambda_1}^{NH}}{\varepsilon_{\lambda_1}^{NO} \bullet \varepsilon_{\lambda_2}^{NH} - \varepsilon_{\lambda_2}^{NO} \bullet \varepsilon_{\lambda_1}^{NH}}\right]$$

$$x_{NH} = \frac{1}{c_0 b} \bullet \left[\frac{A_{\lambda_2} \bullet \varepsilon_{\lambda_1}^{NO} - A_{\lambda_1} \bullet \varepsilon_{\lambda_2}^{NO}}{\varepsilon_{\lambda_1}^{NO} \bullet \varepsilon_{\lambda_2}^{NH} - \varepsilon_{\lambda_2}^{NO} \bullet \varepsilon_{\lambda_1}^{NH}} \right]$$

Where A_{λ_1} and A_{λ_2} are the absorbance at the wavelengths of λ_1 and λ_2, respectively. $\varepsilon_{\lambda_1}^{NO}$, $\varepsilon_{\lambda_1}^{NH}$, $\varepsilon_{\lambda_2}^{NO}$, $\varepsilon_{\lambda_2}^{NH}$ are the molar extinction coefficients of NBNO and NBNH at the wavelengths of λ_1 and λ_2, respectively. c_0 is the initial concentration of NBNO and b is the light path length (b = 0.2 cm), x_{NO} and x_{NH} are the mole fractions of NBNO and NBNH at any irradiation time points. Note that the quantitative conversion of NBNO to NBNH led to the release of an equivalent amount of NO. Therefore, the NO release amounts can thus be calculated by UV-vis spectroscopy.

For micellar nanoparticles, the red light-triggered NO release following a similar protocol. The UV-vis spectra of the nanoparticles were monitored during red light irradiation, and the NO release amounts were calculated based on the recorded UV-vis spectra.

Measurement of the photolysis quantum yield of NBNO in the presence of PdTPTBP

The quantum yield of a photoreaction is defined as follows:

$$\Phi = \frac{number\ of\ reacted\ molecules\ per\ time\ unit}{number\ of\ photos\ absorbed\ per\ time\ unit}$$

The number of photos absorbed per unit was determined by using Reinecke's salt actinometry at excitation wavelengths of 630 nm in an aqueous solution. The mechanism is as follows:

$$[Cr(NH_3)_2(SCN)_4]^- + H_2O \xrightarrow{hv} [Cr(NH_3)_2(SCN)_3(H_2O)] + SCN^-$$

$$Fe^{3+} + SCN^- \longrightarrow Fe(SCN)^{2+}$$

The number of photons was determined from the SCN^- released after irradiation, which was further monitored by the addition of Fe^{3+} to form the blood-red $Fe(SCN)^{2+}$ complex ($\lambda_{abs.}$ = 450 nm, ε = 4300 M^{-1} cm^{-1}). Reineck's salt was commercially available as ammonium salt, which was converted to potassium salt by cation exchange before use. Briefly, the ammonium salt, $NH_4[Cr(NH_3)_2(SCN)_4]$ (1 eq.), was dissolved in warm water (50 ^oC) to give a saturated solution. Excess solid potassium nitrate (4 eq.) was added and dissolved into the above solution. Next, the solution was cooled with ice water and filtered to obtain the crystal precipitate, which was then washed with cold water and dried under vacuum to obtain the product, $K[Cr(NH_3)_2(SCN)_4]$. These operations were carried out in the dark to avoid photodegradation of the product.

The actinomertric procedure was also performed in dark conditions, and the procedure is described as follows. Freshly prepared aqueous solutions of $K[Cr(NH_3)_2(SCN)_4]$ (0.03 M) were used to determine the photon flux of 630 nm LED light. Before use, the solution was filtered with a syringe filter (0.22 μm) to obtain a transparent solution. The above solution (100 μL) was irradiated with 630 nm light for appropriate periods. Another 100 μL of the solution was placed in the same environment without irradiation to serve as a dark control. Next, the solutions were accurately diluted with 0.9 mL of a mixture of 0.1 M $Fe(NO_3)_3$ and 0.5 M $HClO_4$. After incubation in the dark for 5 min,

the solutions were further diluted to 2 mL with water to measure the absorbance at 450 nm in a cuvette. The irradiation time should be short to avoid more than 10% decomposition of $K[Cr(NH_3)_2(SCN)_4]$. The number of photons absorbed per unit time, *Nhv/t*, was calculated based on the above measurement and the following equations:

$$\text{mole } SCN^- = \frac{\Delta A_{450\,nm}}{l \times \varepsilon_{450\,nm}} \times 20$$

$$\frac{Nhv}{t} = \frac{moles\ of\ SCN^-}{\Phi_\lambda \times t \times F}$$

Where $\Delta A_{450\,nm}$ is the absorption difference between the irradiated solution and the dark control; l is the optical path length of the cuvette; $\varepsilon_{450\,nm}$ (= 4300 $M^{-1}\,cm^{-1}$) is the extinction coefficient of $Fe(SCN)^{2+}$; Φ_λ is the quantum yield of $K[Cr(NH_3)_2(SCN)_4]$ at the irradiation wavelength (Φ_{630} = 0.28); t is the irradiation time; F is the absorption correction factor, which is given by $F = 1\text{-}10^{-OD}$ (OD represents the absorption value of the $K[Cr(NH_3)_2](SCN)_4)$ solution at 630 nm).

The number of reacted molecules per unit time was determined by HPLC analysis of the DMSO solution of NBNO (0.25 mM) and PdTPTBP (0.025 mM) with the same irradiation wavelength and intensity as the above photo flux measurements. Briefly, the mixture solution (100 μL) was transferred to a quartz cell and exposed to 630 nm light irradiation for 1 min. The irradiated solution was diluted with 900 μL of a mixture of CH_3CN/H_2O (*v/v*, 1/1) and analyzed by HPLC (Supplementary Fig. 11).

pH titration experiments

In a typical experiment using **BP3** diblock copolymers as an example, 10 mg of **BP3** was initially dissolved in 5 mL of a 0.1 M HCl solution to achieve a polymer concentration of 2.0 mg/mL. Acid-base titration was conducted by gradually adding small volumes of a 0.1 M NaOH solution while maintaining constant stirring. The pH values during the titration process were measured using a Mettler Toledo pH meter (FE20). The complete protonated state (100% protonation degree) and deprotonated state (0% protonation degree) of **BP3** were determined by identifying the two extreme value points on the first derivation of pH titration curves. Similar titration procedures were followed for the other polymers.

Dissolved oxygen measurement

The O_2 concentration measurements were acquired by an oxygen electrode (JPSJ-605F, INESA Scientific Instrument Co., Ltd). The micelles (2.5 mL) were respectively added into a tube, and the oxygen electrode was submerged in the micelle dispersion. Under 630 nm light irradiation, the concentrations of dissolved oxygen were continuously recorded.

EPR tests of NO release

EPR spectroscopy was recorded on a JES-FA200 (JEOL) spectrometer. The measurements were conducted at room temperature and the following parameters were used: modulation frequency: 100 kHz; modulation amplitude: 0.35 mT; scanning field: 324.3 ± 5 mT; microwave power: 1 mW; microwave frequency: 9.063 GHz. PTIO was used as a spin-trapping agent. The micelle concentration was 0.2 g/L containing PTIO(30 μM) in all cases and the mixture was subjected to 630 nm light irradiation at pre-determined time intervals.

Monitoring NO release of micelles by NOFP

Micelles (0.2 g/L, PBS 7.4, 10 mM) were irradiated under 630 nm light (39 mW/cm^2), and the irradiated dispersion (1 mL) was immediately mixed with NOFP solution (50 μM). The mixture was stirred at room temperature for 5 min and the fluorescence intensities were measured.

Cytotoxicity assay

L929 cells were cultured in DMEM supplemented with 10% fetal bovine serum (FBS) and antibiotics (100 units/mL penicillin and 0.1 mg/mL streptomycin). The cell culture was maintained at 37 °C in a humidified atmosphere containing 5% CO_2. To determine cell viability, L929 cells were seeded onto a 96-well plate at a density of 10,000 cells per well, with each well containing 100 μL of DMEM. After 24 h of incubation, the DMEM was replaced with 100 μL of fresh medium containing the desired micellar nanoparticles. Following an additional 24 h incubation period, 10 μL of MTT reagent in PBS buffer (5 mg/mL) was added to each well. The cells were then incubated for another 4 h. Subsequently, the culture medium in each well was removed and replaced by 100 μL of DMSO. The absorbance values were recorded at a wavelength of 490 nm using a microplate reader (Thermo Fisher Scientific). The cell viability was calculated using the following equation:

$$\text{Cell viability}(\%) = \frac{\left(A_{490\ nm,treated} - A_{490\ nm,blank}\right)}{\left(A_{490\ nm,control} - A_{490\ nm,blank}\right)} \times 100\%$$

Where $A_{490\ nm,treated}$ and $A_{490\ nm,control}$ are the absorbance values in the presence and absence of micelles. $A_{490\ nm,blank}$ is the absorbance value of the plate with an identical volume of MTT solution without cells, respectively. The cytotoxicity assay against RAW 264.7 cells followed a similar protocol.

Hemolysis assay

The hemolytic activity of micellar nanoparticles was tested using sheep red blood cells (2%) (purchased from Jiangsu Kejing Biological Technology Co., Ltd.). Initially, the erythrocyte dispersion was centrifuged at 371 g for 10 min. The cells were then resuspended in PBS, and this process was repeated three times to remove hemoglobin from the cells. Next, the micellar nanoparticles were diluted in PBS at concentrations ranging from 0.1 mg/mL to 0.0625 mg/mL using a two-fold gradient dilution. A positive control was prepared using 1% Triton X-100 in PBS, while a negative control consisted of pure PBS. Equal volumes of the erythrocyte suspension and each concentration of the micellar nanoparticles were mixed and incubated at 37 °C for 1 h. After incubation, the mixture was centrifuged at 371 g for 10 min. Subsequently, 100 μL of the supernatant was transferred to a 96-well plate, and the absorbance at 576 nm was measured. The percentage of hemolysis was calculated using the following equation:

$$\text{hemolysis}(\%) = \frac{\left(A_{576\ nm,treated} - A_{576\ nm,blank}\right)}{\left(A_{576\ nm,control} - A_{576\ nm,blank}\right)} \times 100\%$$

where $A_{576\ nm,treated}$ is the absorbance in the presence of micelles, $A_{576\ nm,control}$ is the absorbance in the presence of Triton X-100, and $A_{576\ nm,blank}$ is the absorbance

of the plate with an identical volume of PBS, respectively.

Detection of red light-triggered NO release in biofilms

The CRPA biofilms were cultured according to the above method. Briefly, after various treatments, the biofilms were homogenized by ultrasound (200 W, 40 kHz) for 20 min. The homogenized biofilms were then centrifuged to separate the supernatant (6.0×10^3 g), which contained the released compounds, from the remaining biofilm debris. The supernatant was collected for further analysis. Griess reagent was then added to the collected supernatant, and incubated at 37 ^{o}C for 10 min to allow the reaction to occur. After the incubation, the absorbance of the samples was measured at OD530 using a microtiter plate reader. To quantify the nitrite concentrations, the measured OD530 values were compared to the calibration curve. The corresponding nitrite concentration was determined based on the linear relationship between the absorbance and concentration. All assays included three replications and were repeated in three independent experiments.

Confocal laser scanning microscopy (CLSM) characterization of NO release in CRPA biofilm

The CRPA biofilms were cultured according to the above method. Once the biofilms were formed, they were treated with different groups: PBS, **G1**, **G2**, and **BP1** micelles mixture with NOFP (50 μM), respectively. After the treatment, the biofilms were incubated at 37 ^{o}C for 1 h and then washed with PBS to remove any micelles present on the surface. Next, the treated biofilms were subjected to irradiation with 630 nm light irradiation (39 mW/cm^2) for 30 min, while some groups were left unirradiated as control. The biofilms were stained with Nile red (50 μM) for 15 min. After staining, the biofilms were washed with PBS to remove any excess NOFP. A 488 nm laser was used to excite the NOFP, while a 543 nm laser was used to excite the Nile red.

Hematoxylin and eosin (H&E) and Masson trichrome staining

Following the harvesting of the wounds and surrounding tissues, they were fixed in a solution of neutral buffered formalin (10%) for 24 h. After fixation, the tissues were embedded in paraffin and sectioned sequentially at a thickness of 4 μm using a Leica microtome (RM2016). H&E staining was performed on the skin sections to assess granulation tissue formation and wound maturity. Masson trichrome staining was employed to evaluate the extent of collagen deposition in the healed tissues during the wound-healing process. All histological analyses were conducted on a minimum of 4 independent wounds. Representative images presented in the study were chosen from these multiple replicates. Whole-section images were captured using an IX71 microscope (Olympus) to provide a comprehensive view of the tissue sections.

CD31 and TNF-α immunofluorescent staining

Skin sections embedded in paraffin were cut into slices with a thickness of 4 μm. An antibody directed against the murine endothelial cell surface marker (CD31, Cat#GB11063-2-100 and TNF-α, Cat#GB11188-100, Servicebio Biological, China, used at a dilution of 1:200) was employed to assess the levels of endothelial cell proliferation and the amounts of inflammatory factors in the wound section during the process of wound healing. After treating the blocking solution (5%, w/v, BSA in distilled water) to avoid non-specific binding sites, the primary antibody was applied

for 1.5 h at room temperature, followed by exhaustive washing with PBS for 20 min. Subsequently, a secondary antibody conjugated to Cy3® Goat Anti-Rabbit (IgG) (Cat#GB21303, Servicebio Biological, China, used at a dilution of 1:300) was added and incubated for 1 h to label the antigen. All the amounts of antibodies for staining were followed the manufacturer's instructions. Finally, the sections were mounted on a coverslip in the darkness at 4 ^{o}C. Fluorescence images were captured using 543 nm excitation and the emission channel was set as 570 ~ 600 nm.

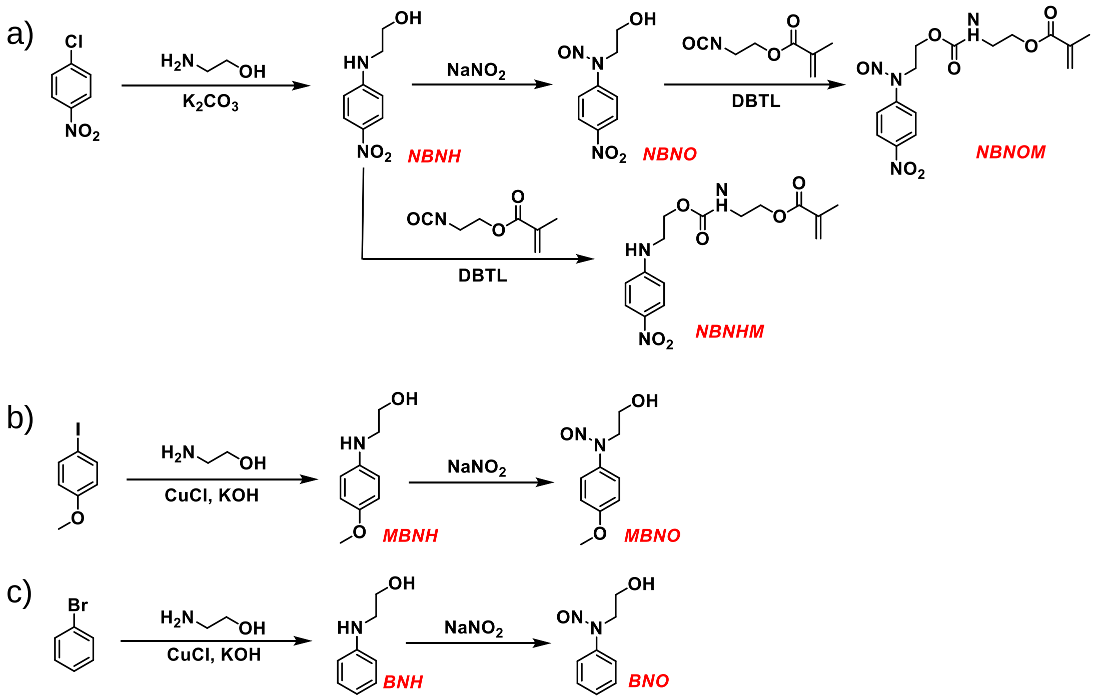

Supplementary Fig. 1. Synthetic routes of (a) NBNOM and NBNHM, (b) MBNO, and (c) BNO.

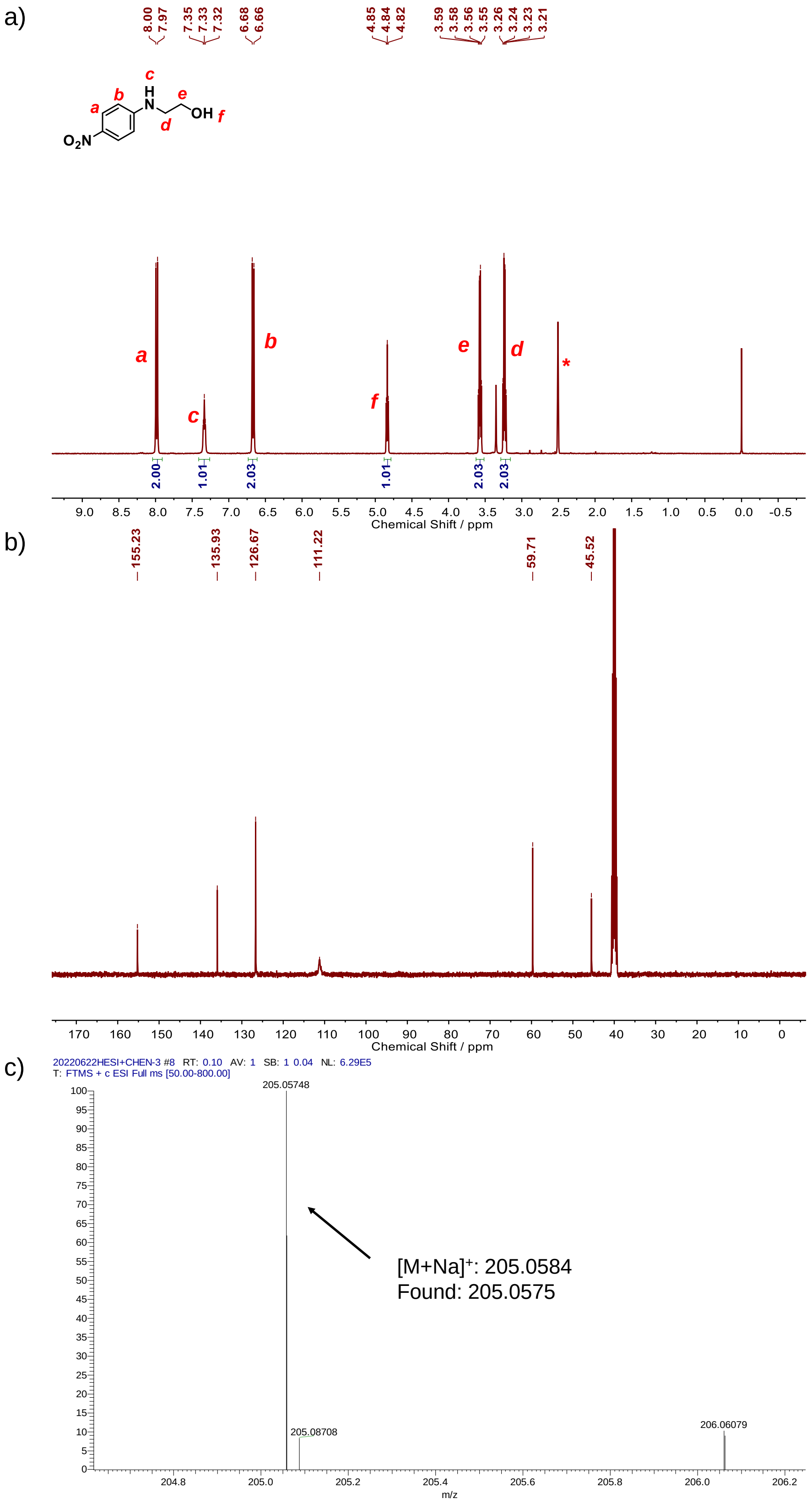

Supplementary Fig. 2. (a) ^{1}H and (b) ^{13}C NMR in $CDCl_3$ and (c) ESI mass spectra recorded for NBNH.

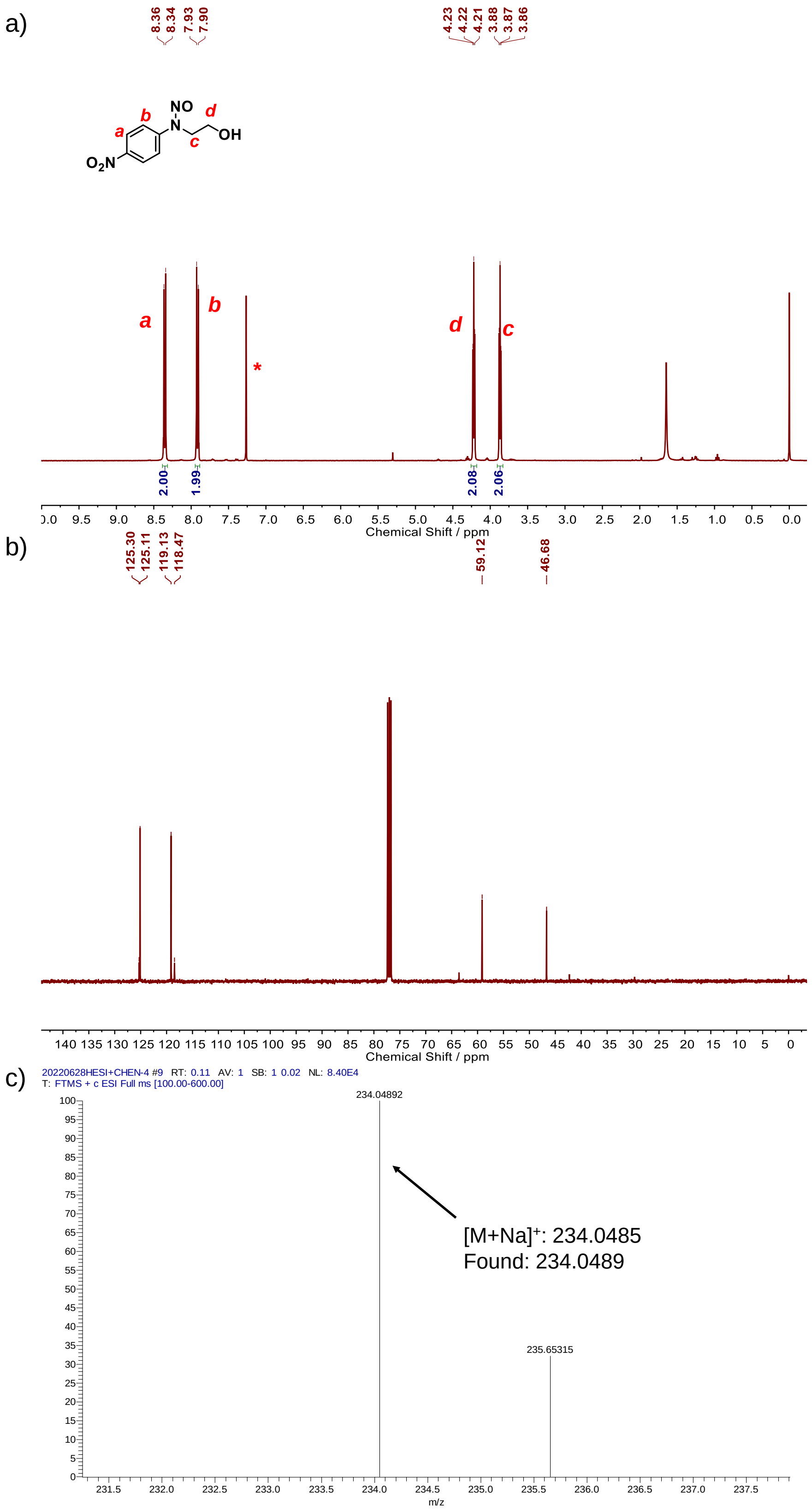

Supplementary Fig. **3**. (a) ^{1}H and (b) ^{13}C NMR in $CDCl_3$ and (c) ESI mass spectra recorded for NBNO.

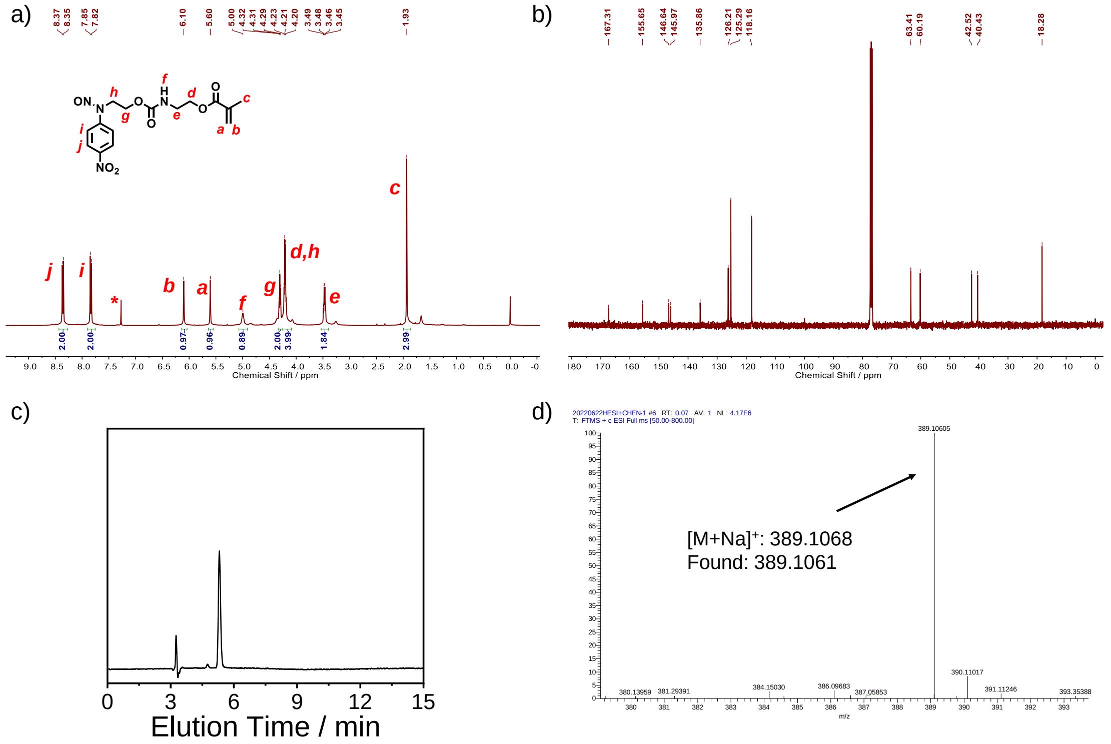

Supplementary Fig. 4. (a) ^{1}H and (b) ^{13}C NMR spectra recorded in $CDCl_3$ for NBNOM. (c) HPLC elution profile detected at 350 nm for NBNOM using MeCN/H_2O = 6/4 (*v/v*) as the eluent. (d) ESI analysis for NBNOM.

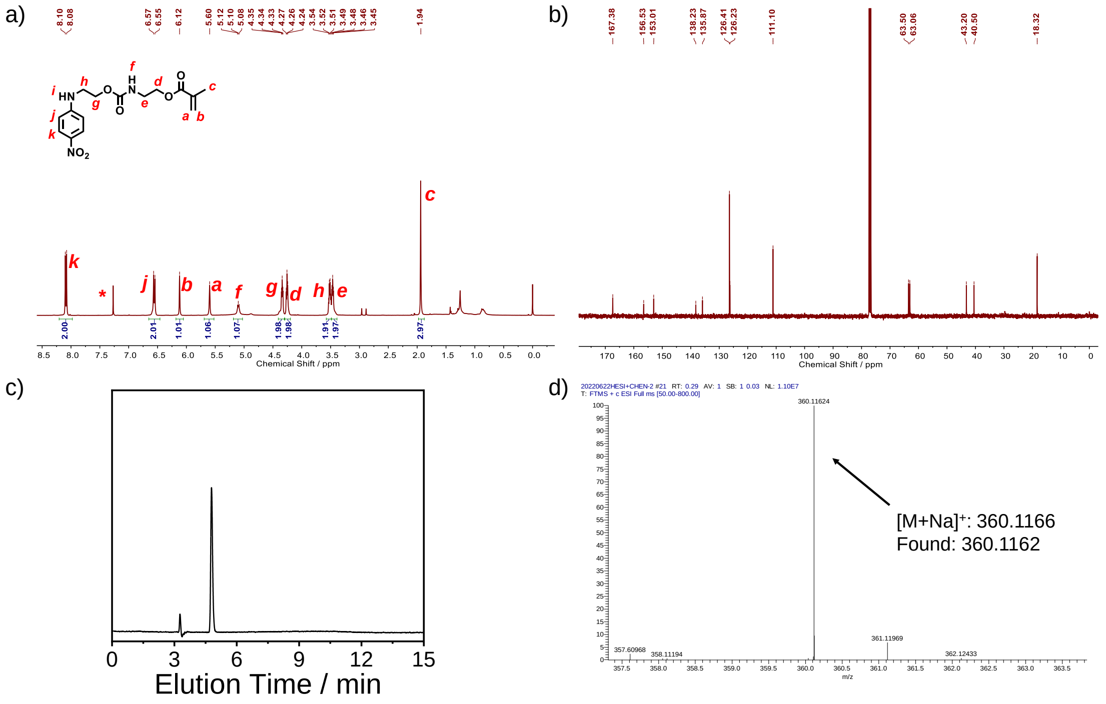

Supplementary Fig. 5. (a) ^{1}H and (b) ^{13}C NMR spectra recorded in $CDCl_3$ for NBNHM. (c) HPLC elution profile detected at 350 nm for NBNHM using $MeCN/H_2O$ = 6/4 (*v/v*) as the eluent. (d) ESI analysis for NBNHM.

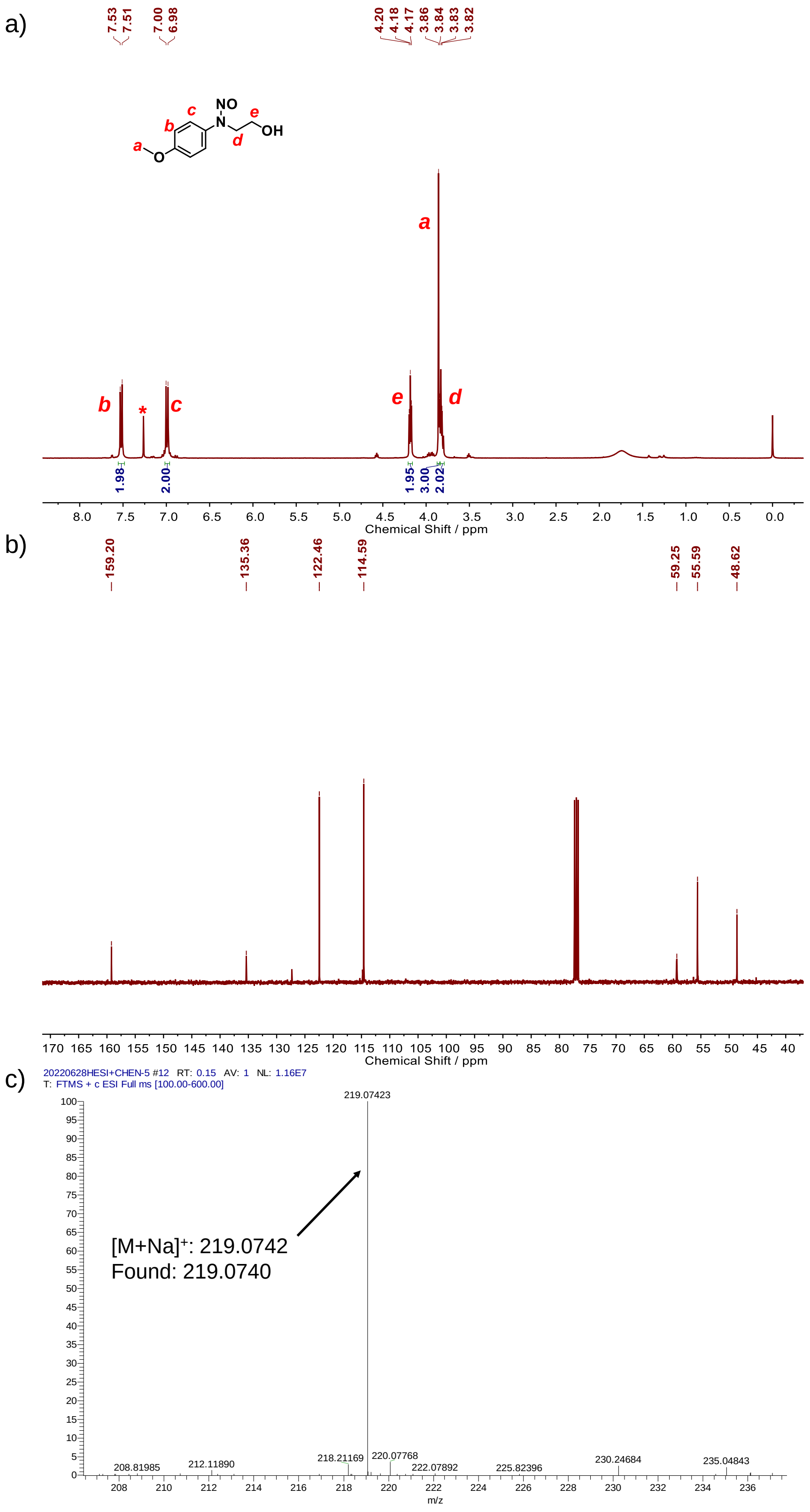

Supplementary Fig. 6. (a) ^{1}H and (b) ^{13}C NMR in $CDCl_3$ and (c) ESI mass spectrum recorded for MBNO.

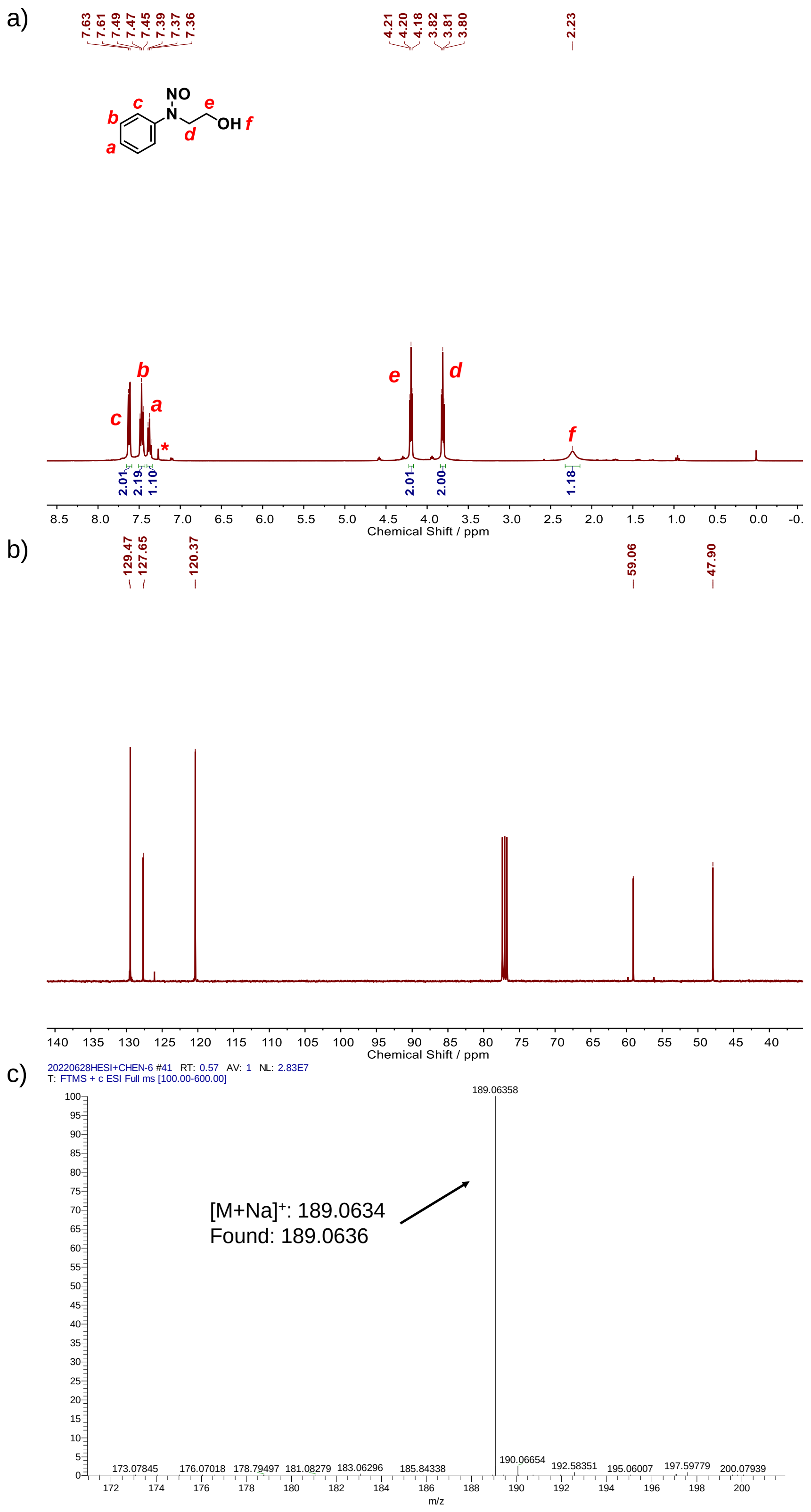

Supplementary Fig. 7. (a) ^{1}H and (b) ^{13}C NMR in $CDCl_3$ and (c) ESI mass spectra recorded for BNO.

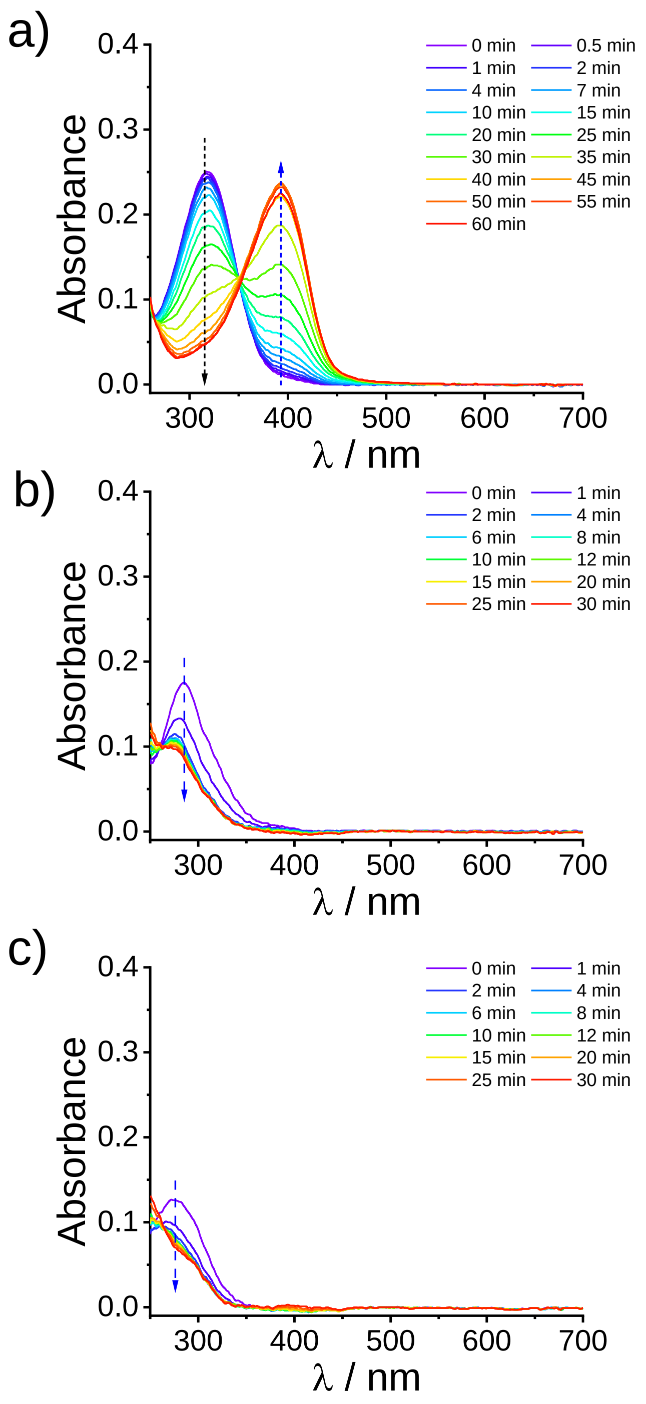

Supplementary Fig. 8. Evolution of UV-vis absorbance spectra of DMSO solutions (100 μM) of (a) NBNO, (b) MBNO, and (c) BNO under 365 nm light irradiation (6.8 mW/cm^2).

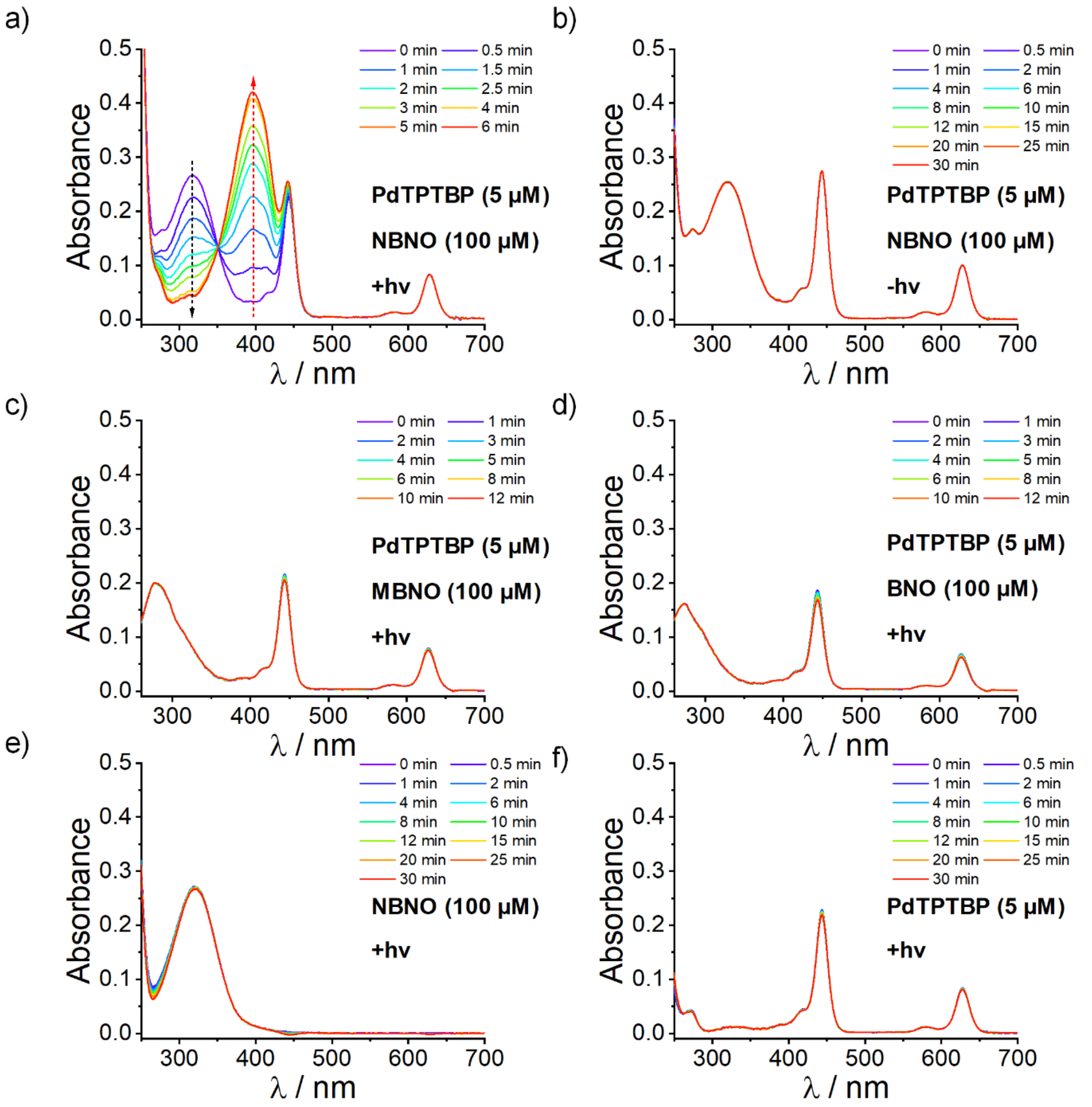

Supplementary Fig. **9**. (a, b) Evolution of UV-vis absorbance spectra of DMSO solutions of PdTPTBP (5 μM)/NBNO (100 μM) (a) with and (b) without 630 nm light irradiation. (c, d) Evolution of UV-vis absorbance spectra of DMSO solutions of (c) PdTPTBP (5 μM)/MBNO (100 μM), and (d) PdTPTBP (5 μM)/BNO (100 μM) with 630 nm light irradiation. (e, f) Evolution of UV-vis absorbance spectra of DMSO solutions of (e) NBNO (100 μM) and (f) PdTPTBP (5 μM) with 630 nm irradiation. In all cases, the irradiation intensity was 39 mW/cm^2.

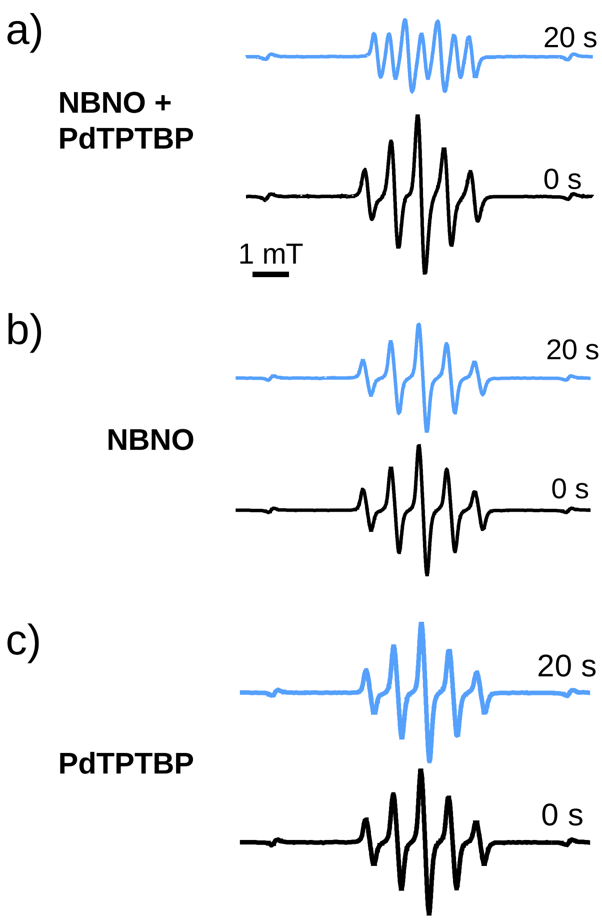

Supplementary Fig. 10. EPR spectra of DMSO solutions of (a) NBNO (100 μM) and PdTPTBP (5 μM), (b) NBNO (100 μM), and (c) PdTPTBP (5 μM) with or without 630 nm irradiation for 20 s. In all cases, the irradiation intensity was 39 mW/cm^2.

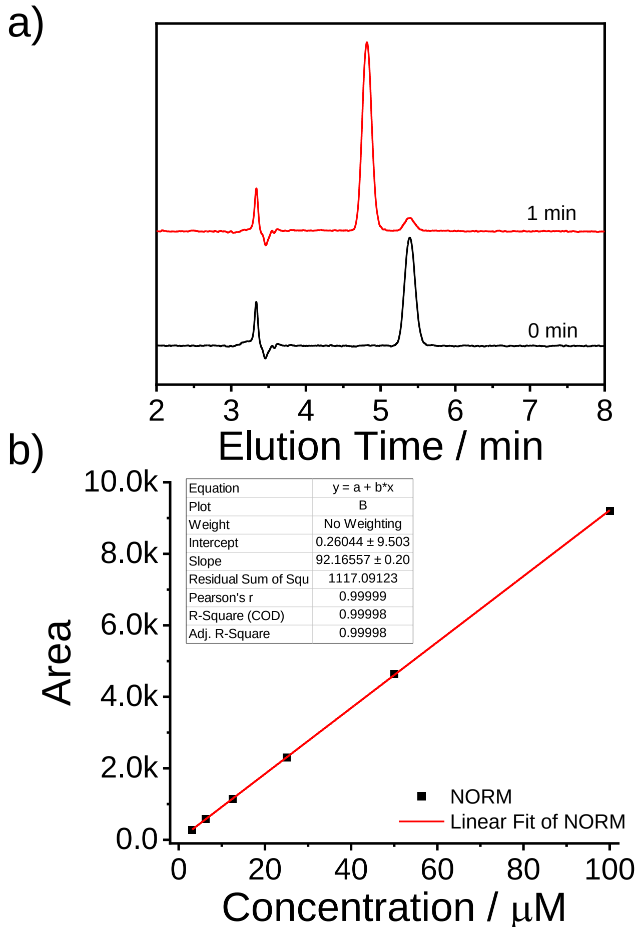

Supplementary Fig. 11. (a) HPLC elution profile detected at 350 nm for NBNO (250 μM)/PdTPTBP (25 μM) mixture in DMSO before and after 630 nm irradiation for 1 min. (b) The integral area was determined by HPLC (350 nm) as a function of the concentrations of NBNO (MeCN/H_2O = 1:1).

Supplementary Notes. Based on the established calibration curve, the reacted content of NBNO within 1 min was determined to be 216.8 μM. The number of photons absorbed per unit time, *Nhv/t*, was calculated using the aforementioned equations. Where $\Delta A_{450\,nm}$ was calculated to be 0.155; l is the optical path length of the cell (l = 0.2 cm); t is the irradiation time (t = 60 s); F is the absorption correction factor (F = 0.45).

$$\text{mole } SCN^{-} = \frac{\Delta A_{450\,nm}}{l \times \varepsilon_{450\,nm}} \times 20 = \frac{0.155}{0.2 \times 4300} \times 20 = 0.003605$$

$$\frac{Nhv}{t} = \frac{moles\ of\ SCN^{-}}{\Phi_{\lambda} \times t \times F} = \frac{0.003605}{0.28 \times 60 \times 0.45} = 0.000477$$

$$\Phi = \frac{number\ of\ reacted\ molecules\ per\ time\ unit}{number\ of\ photos\ absorbed\ per\ time\ unit} = \frac{0.0002168/60}{0.000477} = 0.0076$$

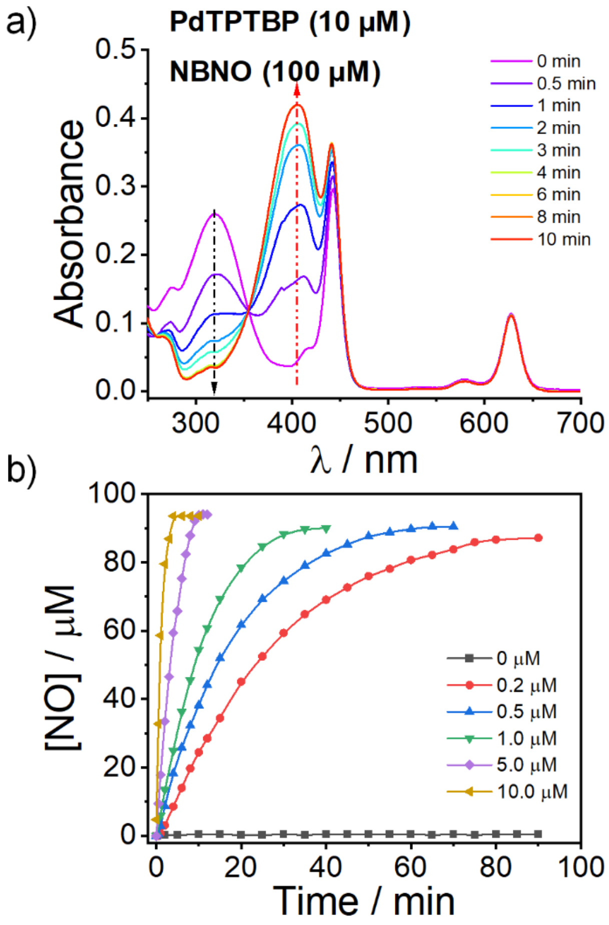

Supplementary Fig. 12. (a) Evolution of UV-vis absorbance spectra of DMSO solutions of PdTPTBP (10 μM)/NBNO (100 μM) under 630 nm light irradiation. (b) Time-dependent released NO contents from NBNO (100 μM) in the presence of different concentrations of PdTPTBP under 630 nm irradiation (39 mW/cm^2).

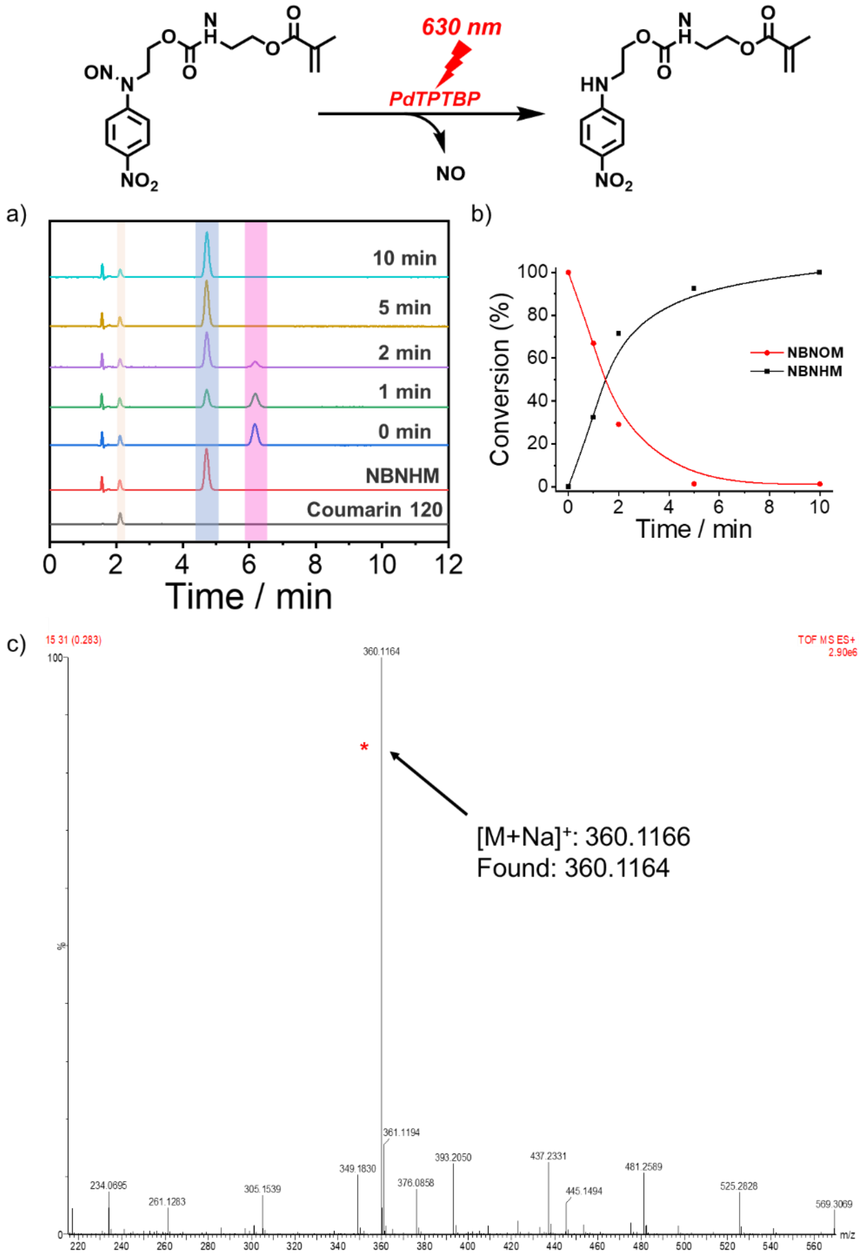

Supplementary Fig. 13. (a) Partial HPLC elution profiles using MeCN: H_2O = 1:1 (*v/v*) as the eluent of **NBNOM** (250 μM) and **PdTPTBP** (25 μM) under 630 nm light irradiation (39 mW/cm^2) for varying times (0, 1, 2, 5, and 20 min). Coumarin 120 was used as an internal standard. (b) Changes in peak areas versus coumarin 120 internal standard of compounds **NBNOM** and **NBNHM**, revealing the exclusive conversion of **NBNOM** to **NBNHM**. (c) HRMS analysis of **NBNOM** and **PdTPTBP** solution under 630 nm light irradiation for 10 min. The asterisk indicates the formation of **NBNHM**.

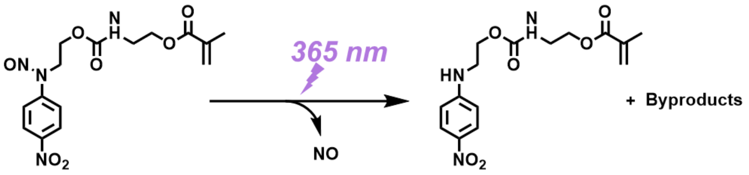

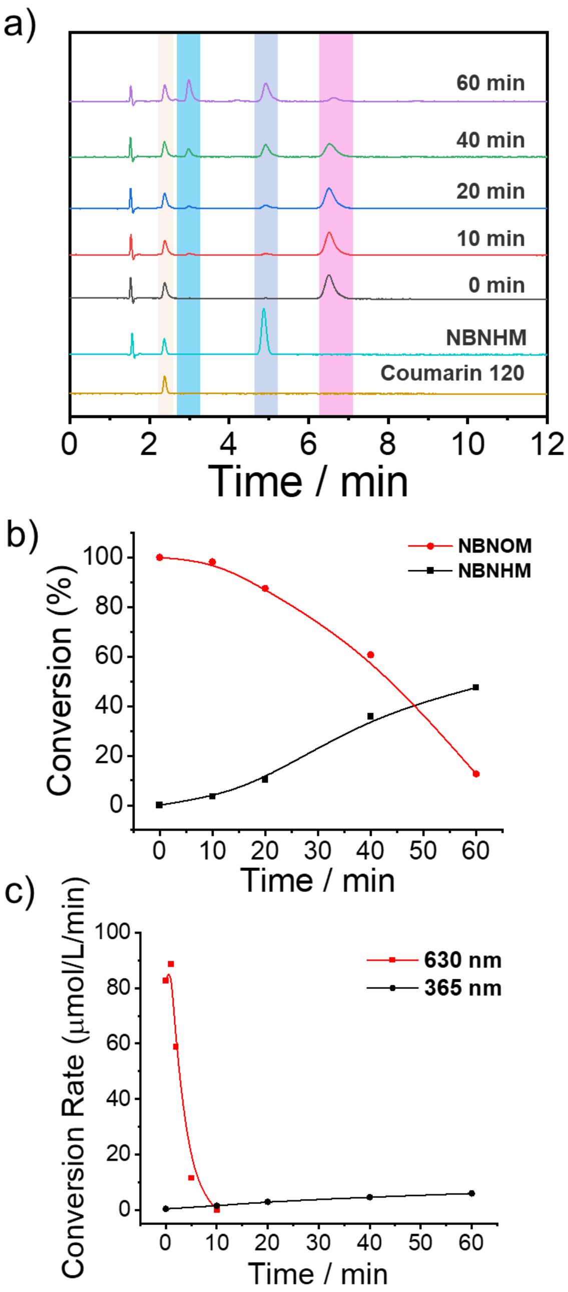

Supplementary Fig. 14. (a) Partial HPLC elution profiles of **NBNOM** (250 μM) under 365 nm light irradiation (6.8 mW/cm^2) for varying times (0, 10, 20, 40, and 60 min) using MeCN: H_2O = 1:1 (*v/v*) as the eluent. Coumarin 120 was used as an internal standard. (b) Peak areas against coumarin 120 internal standard of compounds **NBNOM** and **NBNHM**, revealing the conversion of **NBNOM** to **NBNHM** and byproducts under UV light irradiation.

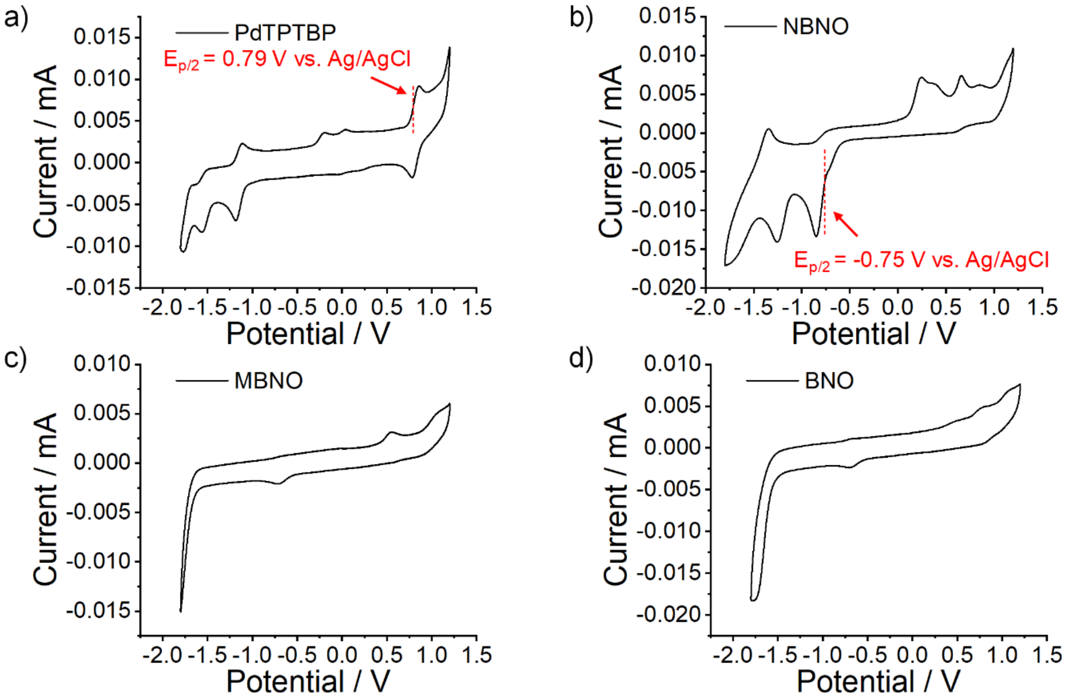

Supplementary Fig. 15. Cyclic voltammogram curves of DMF solutions (a) **PdTPTBP**, (b) **NBNO**, (c) **MBNO,** and (d) **BNO**. The concentration of **PdTPTBP** was 0.5 mM and the concentrations of **NBNO**, **MBNO**, and **BNO** were 1 mM in all cases.

Supplementary Notes. Cyclic voltammetry was carried out using 0.1 M tetrabutylammonium perchlorate as the electrolyte in DMF at 25 °C with N_2 purge for 30 min, and Ag/AgCl was used as the reference electrode. The maximal photoluminescence of PdTPTBP was estimated to be 796 nm, and the $E_{1/2}^{OX}$ of PdTPTBP was determined to be 0.79 V (vs Ag/AgCl, Supplementary Fig. 14). Using photoluminescence maximum and $E_{1/2}^{OX}$, the excited state reduction potential of PdTPTBP was estimated according to the following equations:

$$E_{1/2}(\mathrm{PdTPTBP}^{\bullet+}/\mathrm{PdTPTBP}^{*}) = E_{1/2}{}^{OX} - E_{0,0}$$

$$E_{0,0} = hc/\lambda_{max} = 1240\ \mathrm{nm}/\lambda_{max}$$

As such, the excited state reduction potential was calculated to be $E_{1/2}(\mathrm{PdTPTBP}^{\bullet+}/\mathrm{PdTPTBP}^{*}) = 0.79\ \mathrm{V} - 1240\ \mathrm{nm}/796\ \mathrm{nm} = -0.77\ \mathrm{V}$.

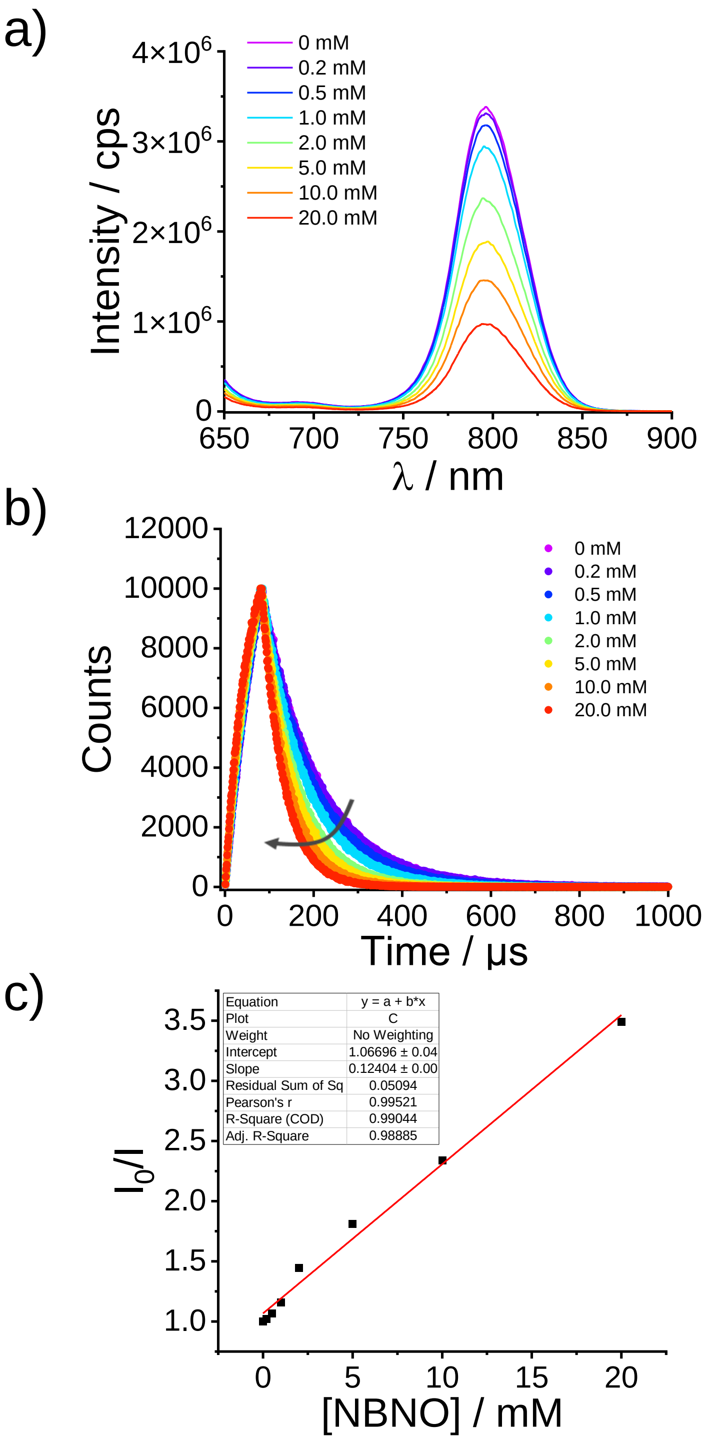

Supplementary Fig. **16**. (a) Steady-state phosphorescence intensity changes (λ_{ex} = 630 nm) of **PdTPTBP** (10 μM) in the presence of varying concentrations of **NBNO**. (b) Phosphorescence lifetime changes of **PdTPTBP** (10 μM) in the presence of different concentrations of **NBNO**, λ_{ex} = 460 nm. (c) Stern-Volmer plot of phosphorescence intensity quenching of **PdTPTBP** (10 μM) in the presence of varying concentrations of NBNO. The samples were degassed via three freeze-pump-thaw cycles prior to measurements.

Supplementary Notes. The mixed solutions were degassed by three freeze-pump-thaw cycles in DMF. The k_{sv} constants was calculated according to the Stern-Volmer equation:

$$\frac{I_0}{I} = 1 + k_{sv} \times Q$$

Where I_0 and I stand for the photoluminescence intensities of **PdTPTBP** (10 μM) in the absence of **NBNO** and in the presence of varying amounts of **NBNO** (0.2, 0.5, 1.0, 2.0, 5.0, 10.0, and 20.0 mM), respectively. Q is the concentration of **NBNO**. The quenching constants (k_q) was calculated by the following equation:

$$k_q = k_{sv}/\tau_T$$

τ_T is the phosphorescence lifetime of **PdTPTBP** in degassed DMF.

The value of k_{sv} was determined to be 124 M^{-1}, and the value of k_q was determined to be 7.33×10^5 $M^{-1}s^{-1}$.

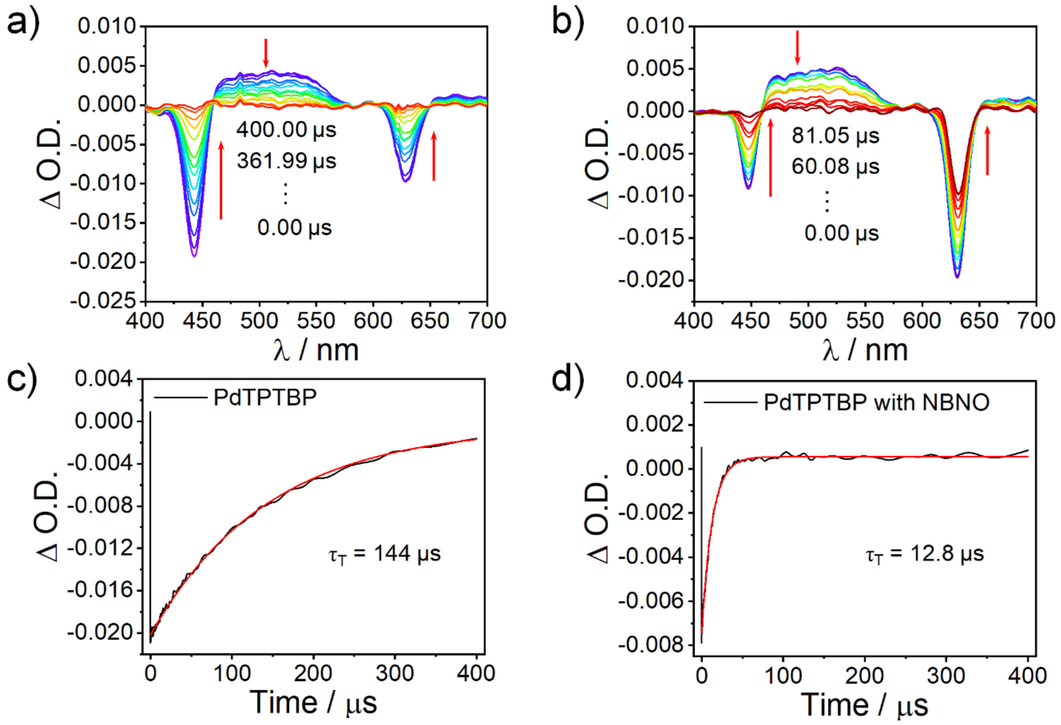

Supplementary Fig. 17. (a, b) Nanosecond time-resolved transient absorption spectra (λ_{ex} = 630 nm) and (c, d) corresponding decay curves at 445 nm of Ar-saturated DMSO solutions of (a, c) **PdTPTBP** (10 μM) and (b, d) **PdTPTBP** (10 μM)/**NBNO** (20 mM) mixture.

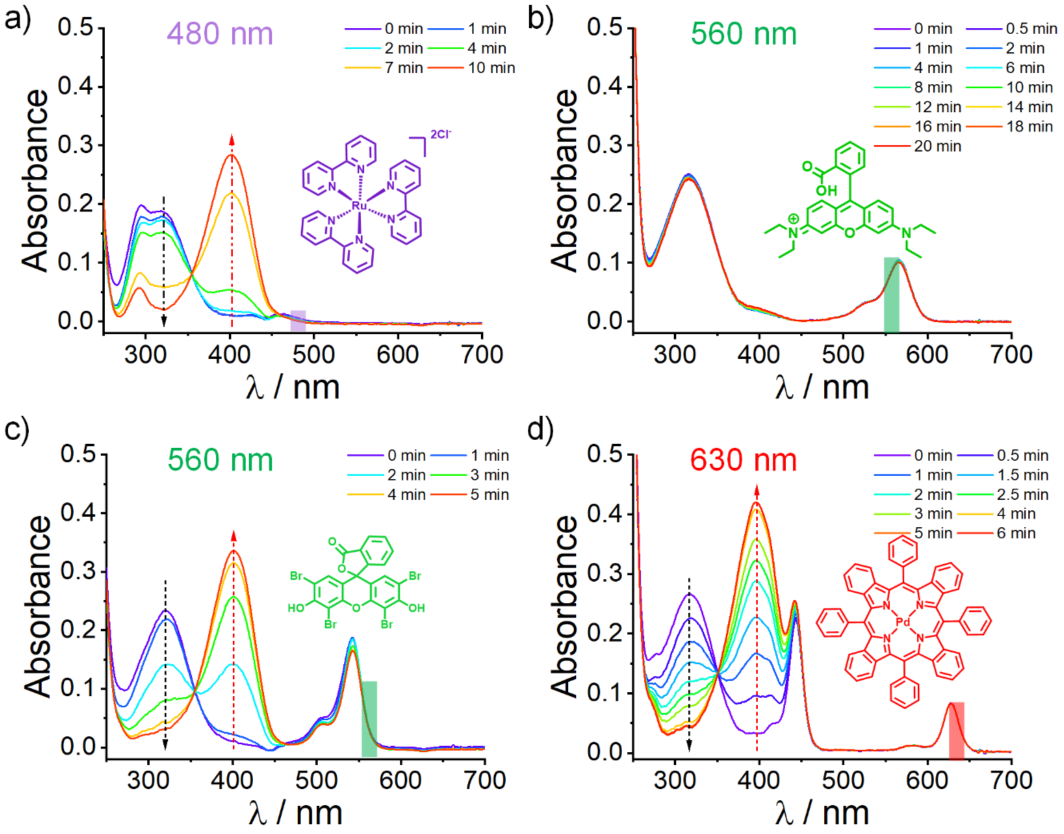

Supplementary Fig. 18. Evolution of UV-vis absorbance spectra of DMSO solutions of **NBNO** (100 μM) in the presence of varying photocatalysts (5 μM): (a) tris(2,2′-bipyridyl)ruthenium chloride hexahydrate with 480 nm LED light irradiation (41 mW/cm^2), (b) Rhodamine B with 560 nm light irradiation (22 mW/cm^2), (c) 2',4',5',7'-tetrabromofluorescein with 560 nm light irradiation (22 mW/cm^2), and (d) **PdTPTBP** with 630 nm LED light irradiation (39 mW/cm^2) (also shown in Supplementary Fig. 8).

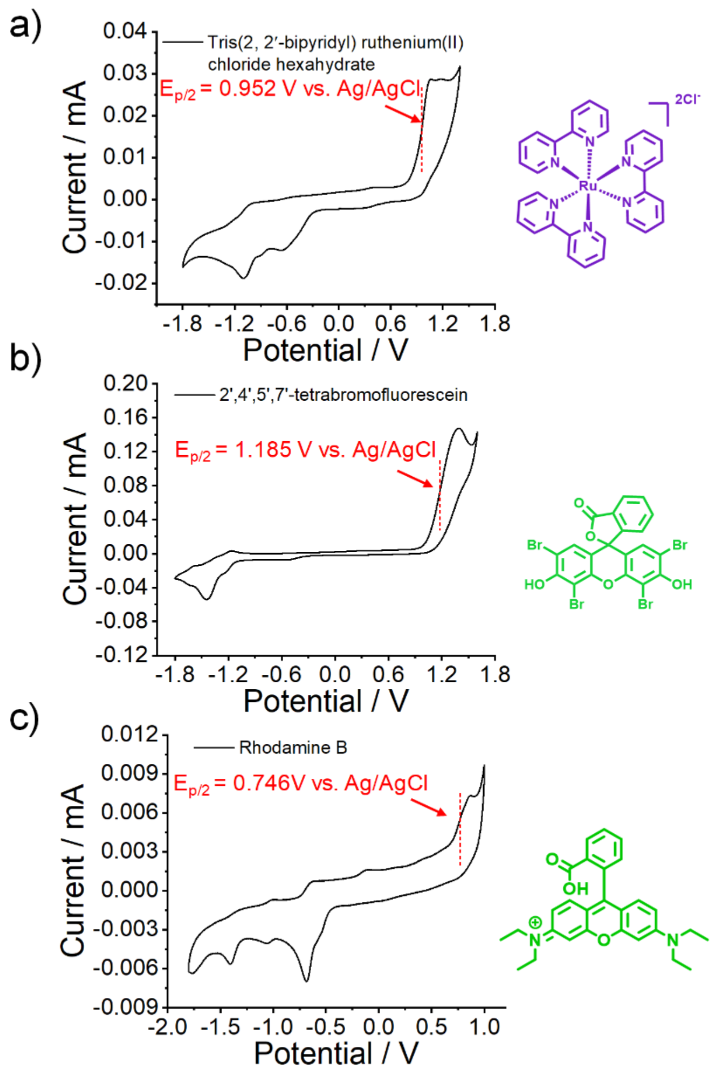

Supplementary Fig. 19. Cyclic voltammogram curves of DMF solutions (a) tris(2,2'-bipyridyl) ruthenium chloride hexahydrate, (b) 2',4',5',7'-tetrabromofluorescein, and (c) Rhodamine B. The photocatalyst concentrations were 0.5 mM in all cases.

Supplementary Notes. The excited state reduction potential of tris(2,2'-bipyridyl) ruthenium chloride hexahydrate, 2',4',5',7'-tetrabromofluorescein, and Rhodamine B was estimated.

$$E_{1/2}\left(Ru(bpy)_3{}^{\bullet+}/Ru(bpy)_3{}^{*}\right) = 0.952\ V - 1240\ nm/620\ nm = -1.05\ V$$

$$E_{1/2}(Eosin\ Y^{\bullet+}/Eosin\ Y^{*}) = 1.185\ V - 1240\ nm/538\ nm = -1.12\ V$$

$$E_{1/2}(RhB^{\bullet+}/RhB^{*}) = 0.746\ V - 1240\ nm/580\ nm = -1.39\ V$$

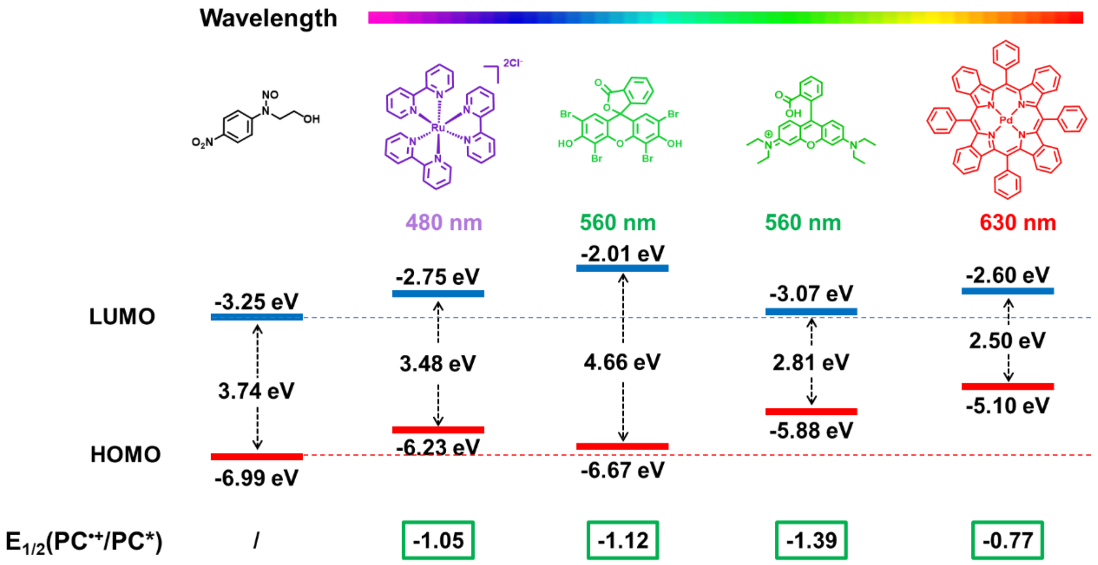

Supplementary Fig. 20. The relative energetic dispositions for the HOMO and LUMO of **NBNO**, tris(2,2′-bipyridyl) ruthenium chloride hexahydrate, 2',4',5',7'-tetrabromofluorescein, Rhodamine B, and **PdTPTBP**.

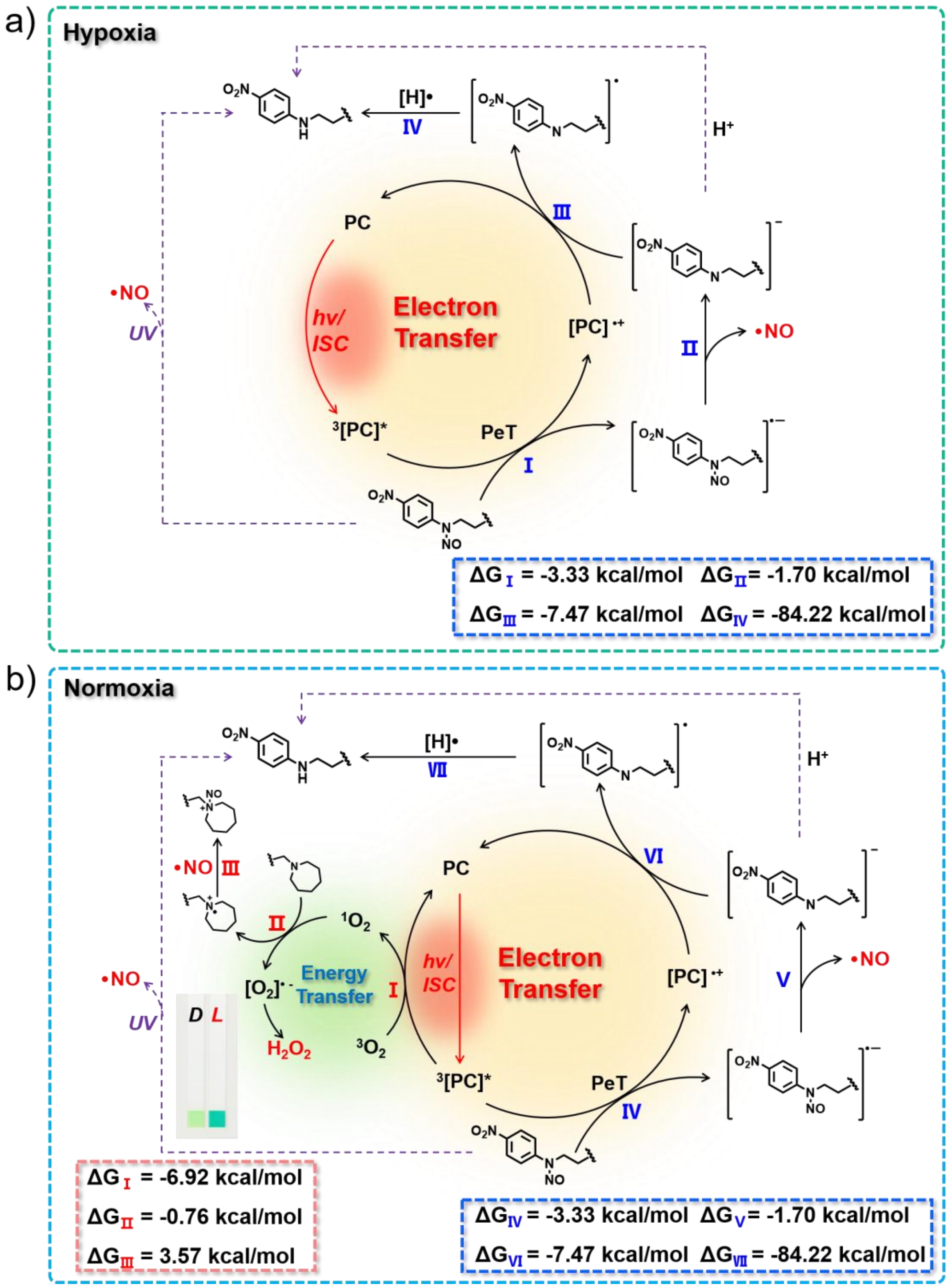

Supplementary Fig. 21. Proposed mechanisms of the activation of **NBNO** with **PdTPTBP** in (a) hypoxic and (b) normoxic conditions. Inset: detection of H_2O_2 using a test strip (D, dark; L, light).

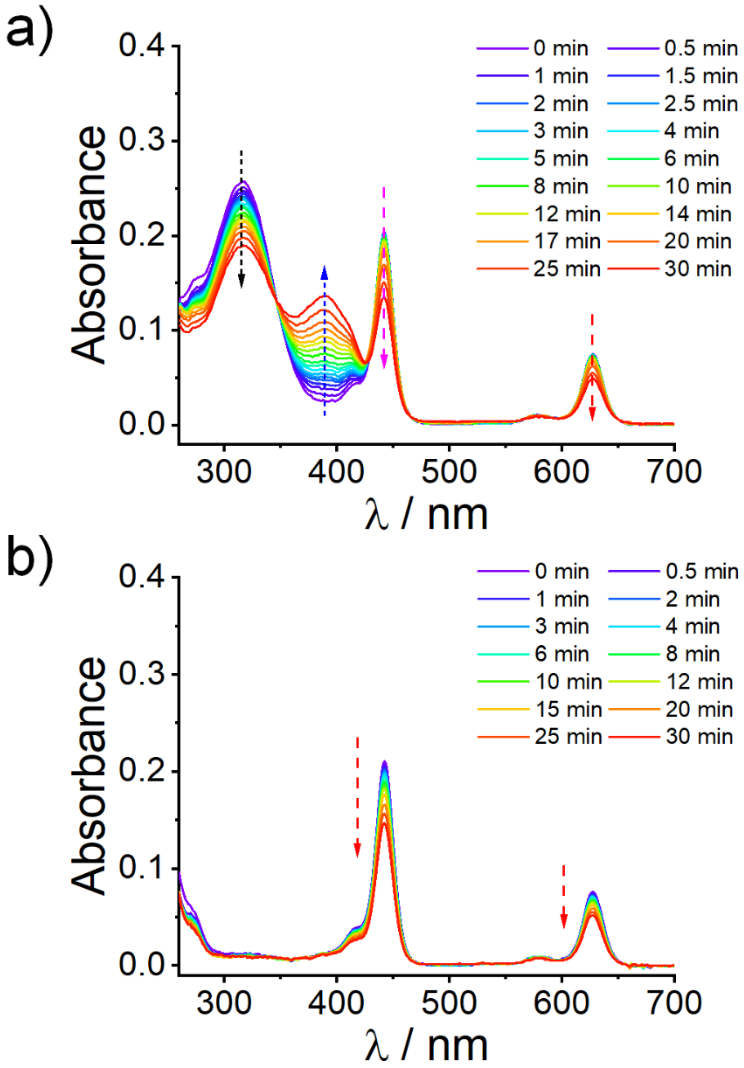

Supplementary Fig. 22. (a) Evolution of UV-vis absorbance spectra of DMF solutions of **PdTPTBP** (5 μM)/**NBNO** (100 μM) mixture under 630 nm light irradiation. (b) Evolution of UV-vis absorbance spectra of DMF solutions of PdTPTBP (5 μM) under 630 nm light irradiation.

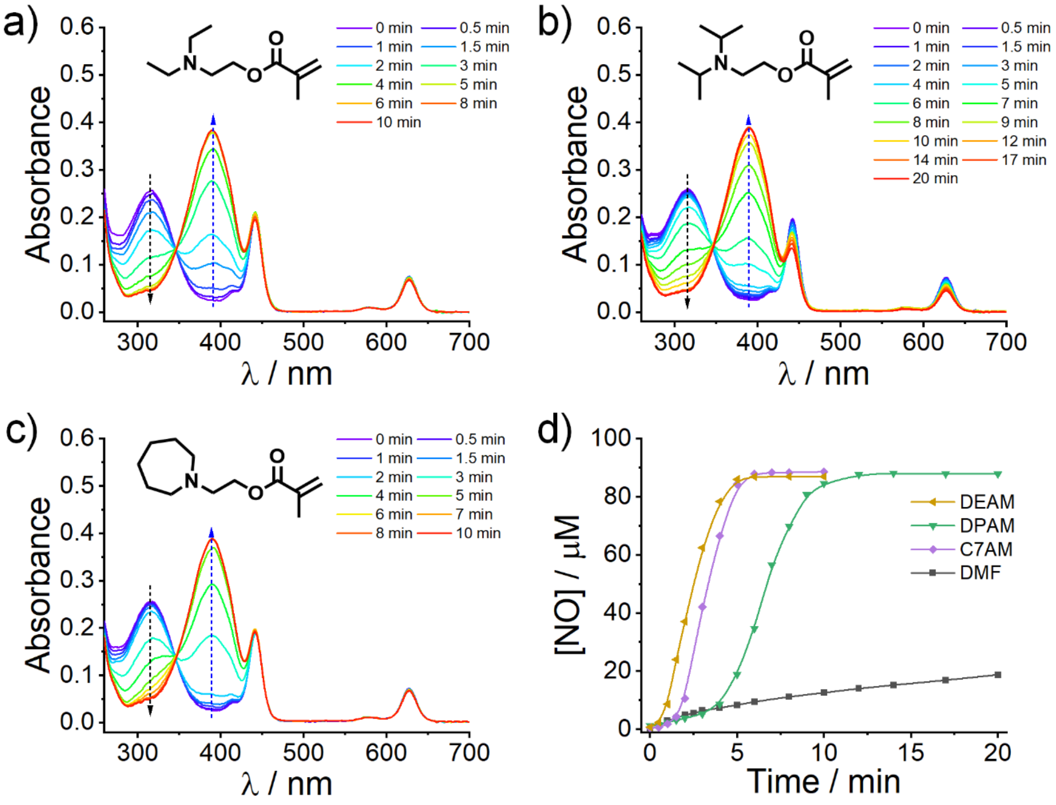

Supplementary Fig. 23. Evolution of UV-vis absorbance spectra of DMF solutions of **PdTPTBP** (5 μM)/**NBNO** (100 μM)/tertiary amine (500 μM) mixtures under 630 nm irradiation. (a) DEAM, (b) DPAM, and (c) C7AM. (d) Time-dependent NO release in the presence of varying tertiary amines under 630 nm irradiation.

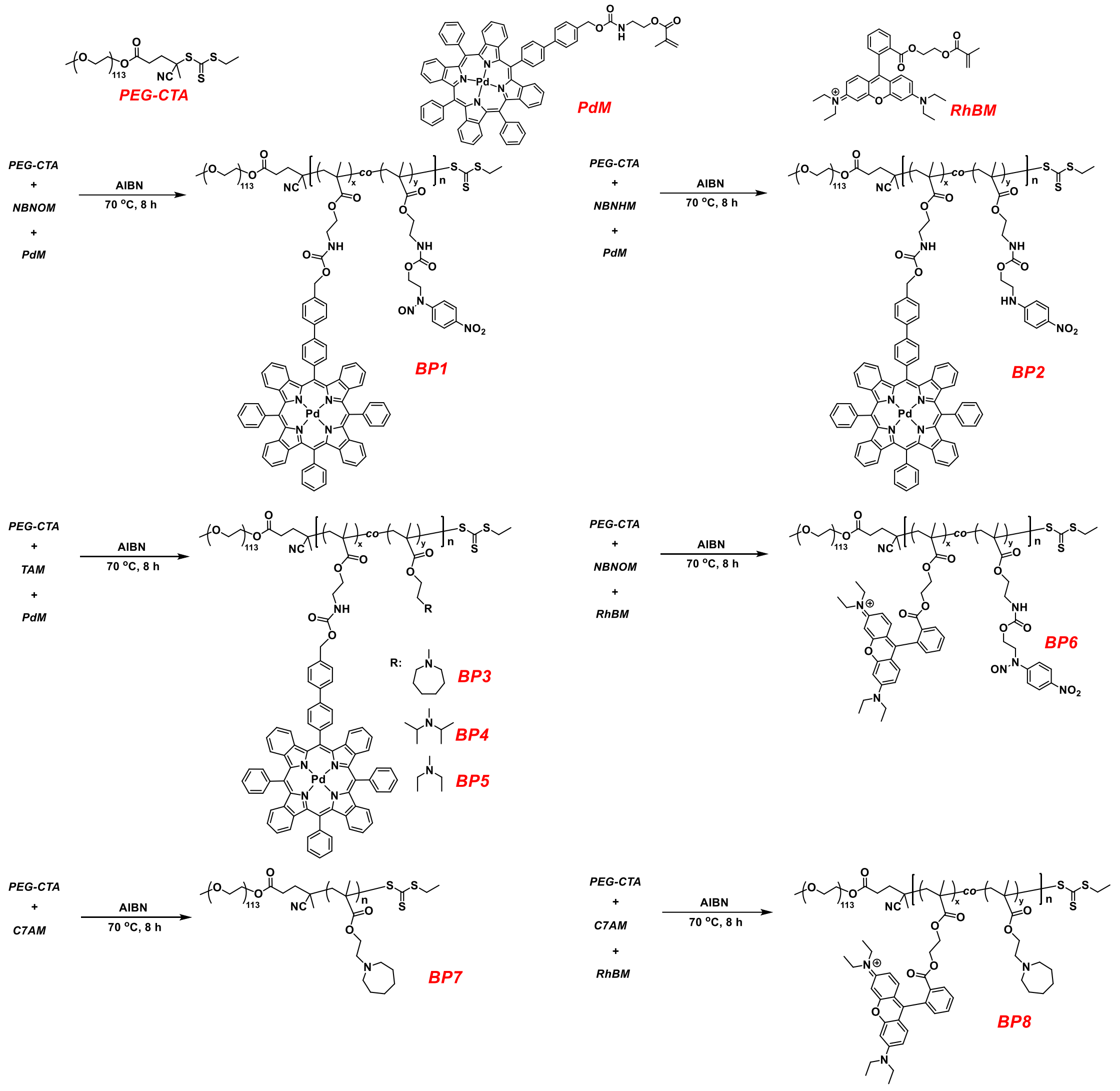

Supplementary Fig. 24. Synthetic routes of **BP1-BP8** diblock copolymers.

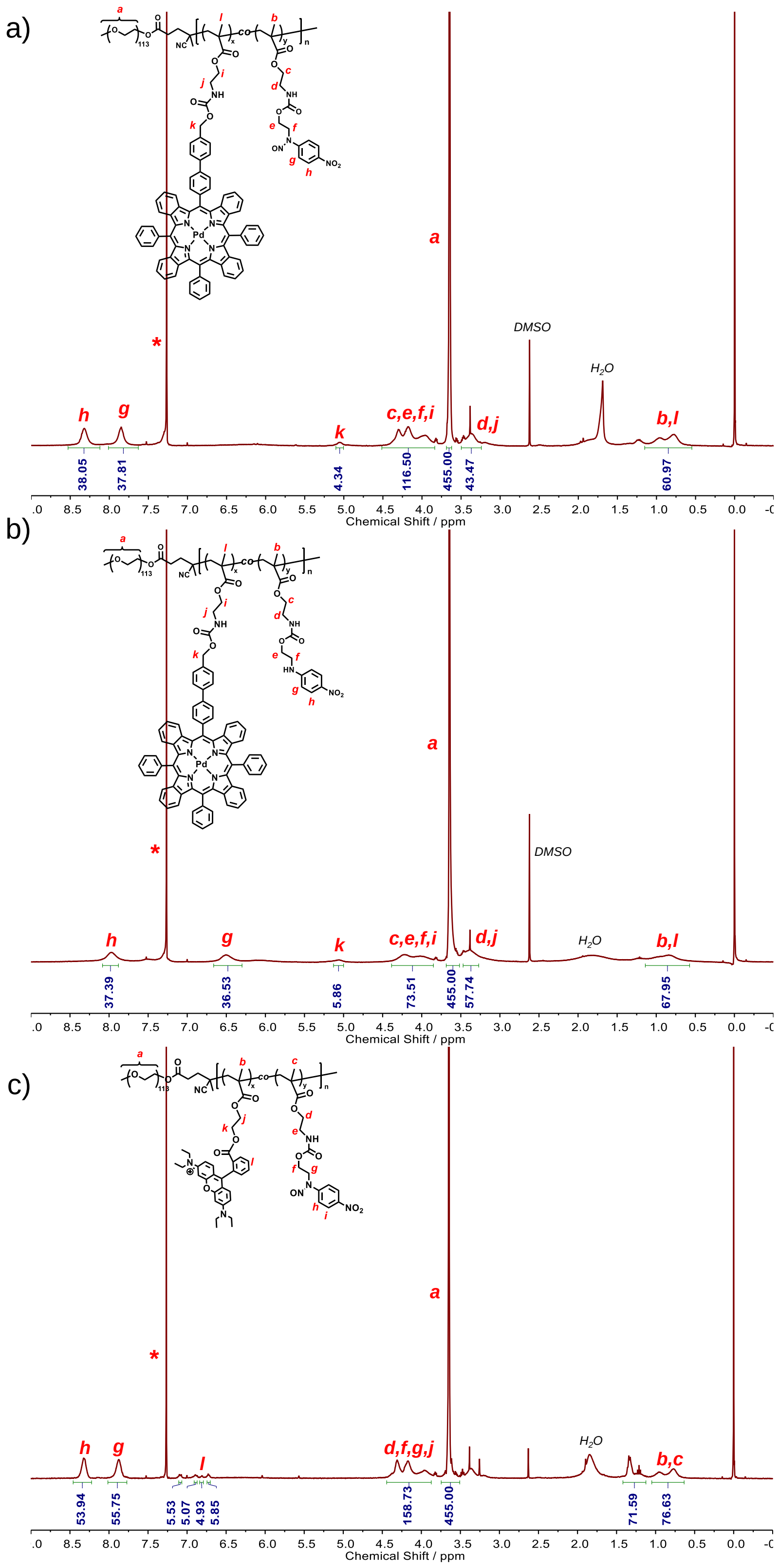

Supplementary Fig. **25**. ^{1}H NMR spectra recorded in $CDCl_3$ for (a) **BP1**, (b) **BP2**, and (c) **BP6** diblock copolymers.

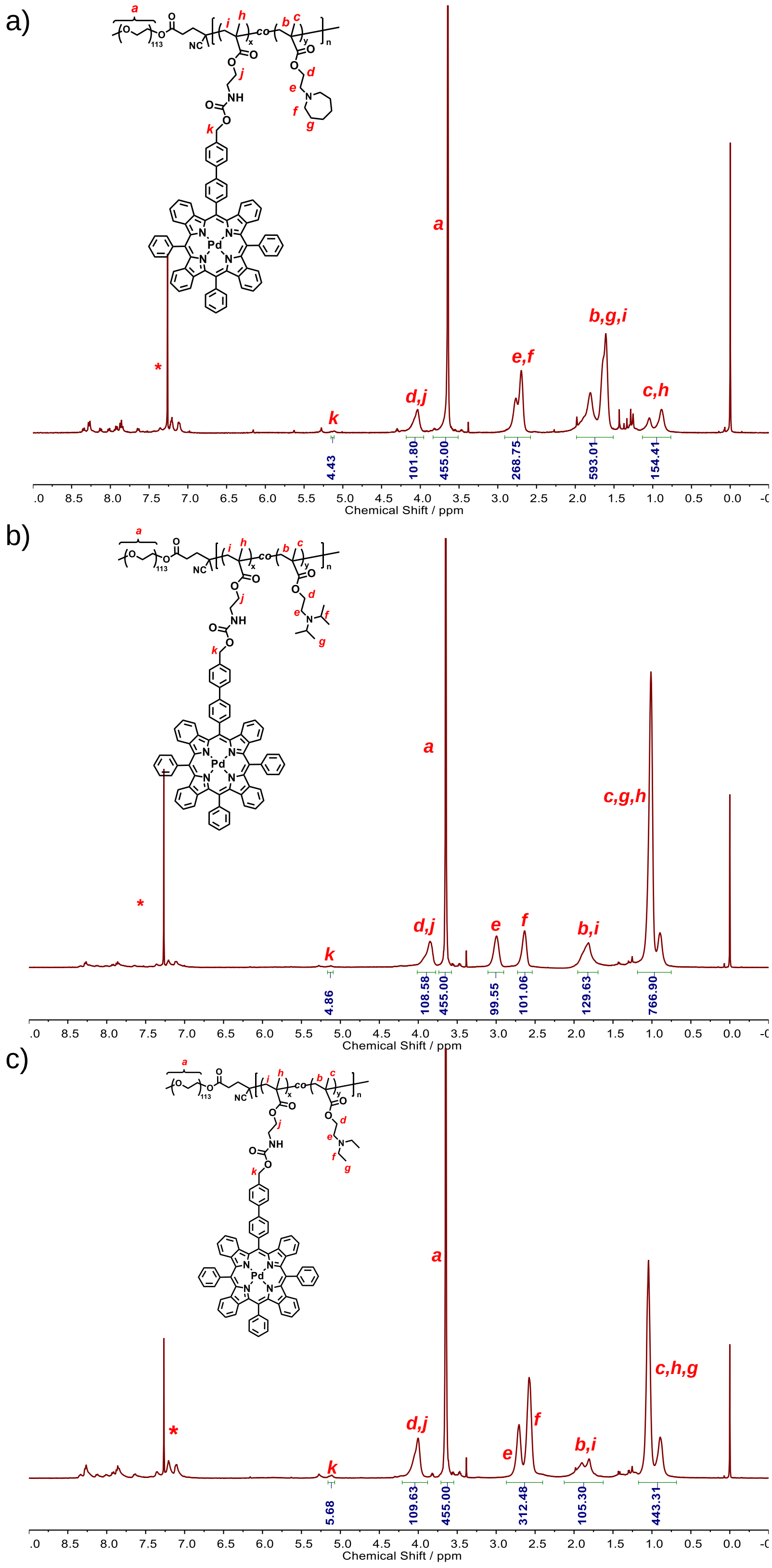

Supplementary Fig. **26**. ^{1}H NMR spectra recorded in $CDCl_3$ for (a) **BP3**, (b) **BP4**, and (c) **BP5** diblock copolymers.

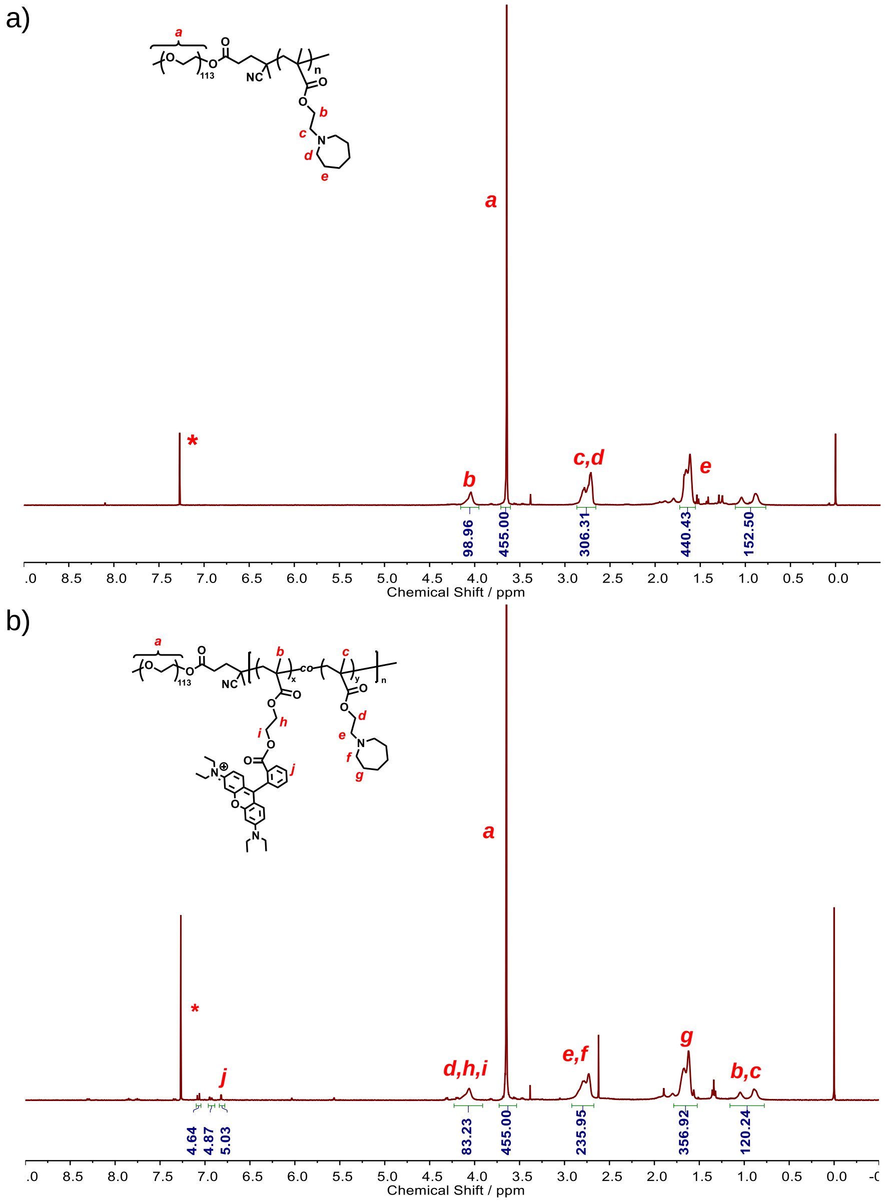

Supplementary Fig. 27. ^{1}H NMR spectra recorded in $CDCl_3$ for (a) **BP7** and (b) **BP8** diblock copolymers.

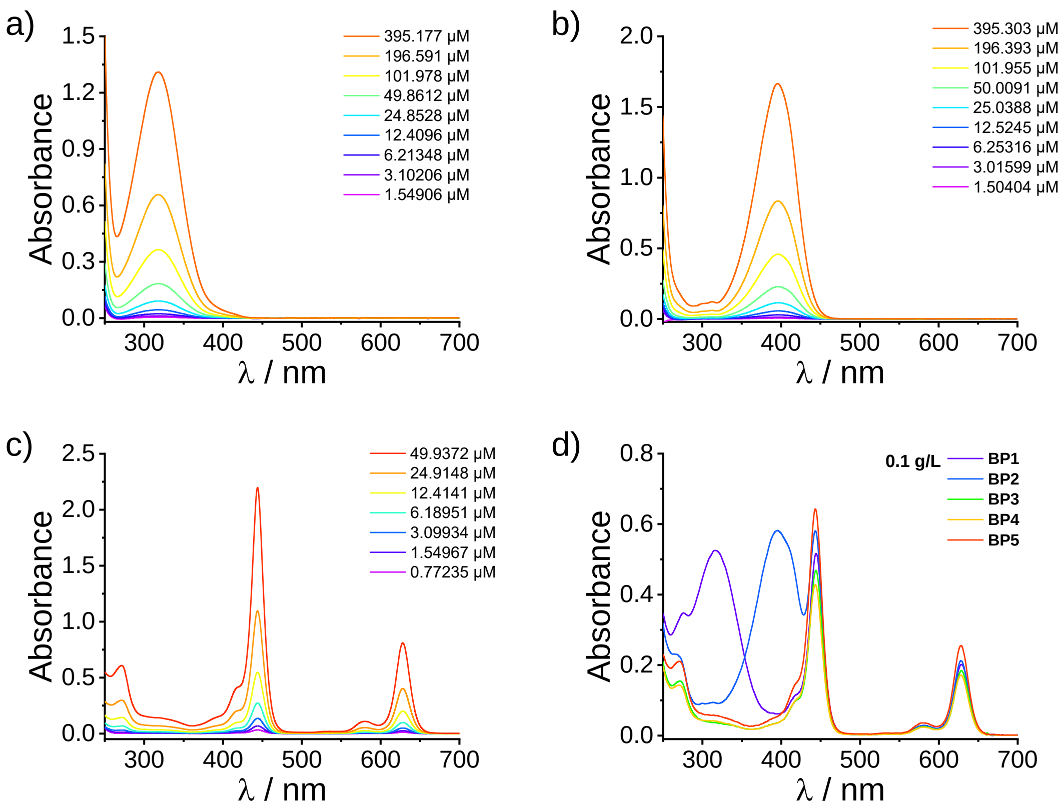

Supplementary Fig. 28. (a-c) Concentration-dependent UV-vis absorbance spectra of (a) **NBNOM**, (b) **NBNHM**, and (c) **PdM** in DMSO. (d) UV-Vis absorption spectra were recorded for DMSO solutions (0.1 g/L) of **BP1**–**BP5** block copolymers.

Supplementary Notes. NBNOM and PdM concentrations within **BP1** at a concentration of 0.1 g/L were determined to be 0.132 mM and 0.0124 mM according to the calibration curve in Supplementary Fig. 28. Alternatively, based on the NMR result depicted in Supplementary Fig. 25, the NBNOM concentration within the same polymer was calculated to be 0.123 mM, and the PdM concentration was found to be 0.0128 mM, in good agreement with results obtained from the UV-vis calibration curve.

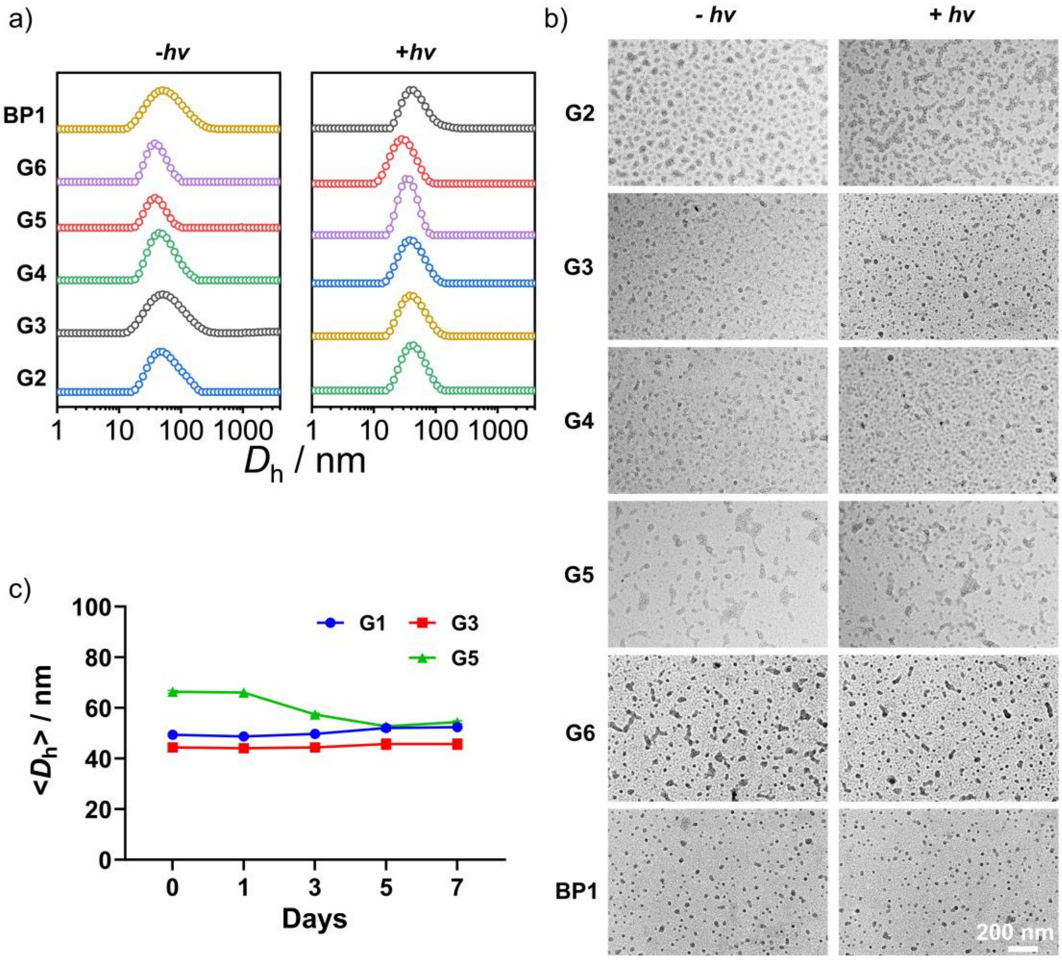

Supplementary Fig. **29**. (a, b) Intensity-average hydrodynamic diameter distributions (a) and TEM images (b) of **G2**-**G6** and **BP1** micelles with or without 630 nm light irradiation for 30 min. (c) Time-dependent changes in the hydrodynamic diameter, <D_h>, of **G1**, **G3,** and **G5**.

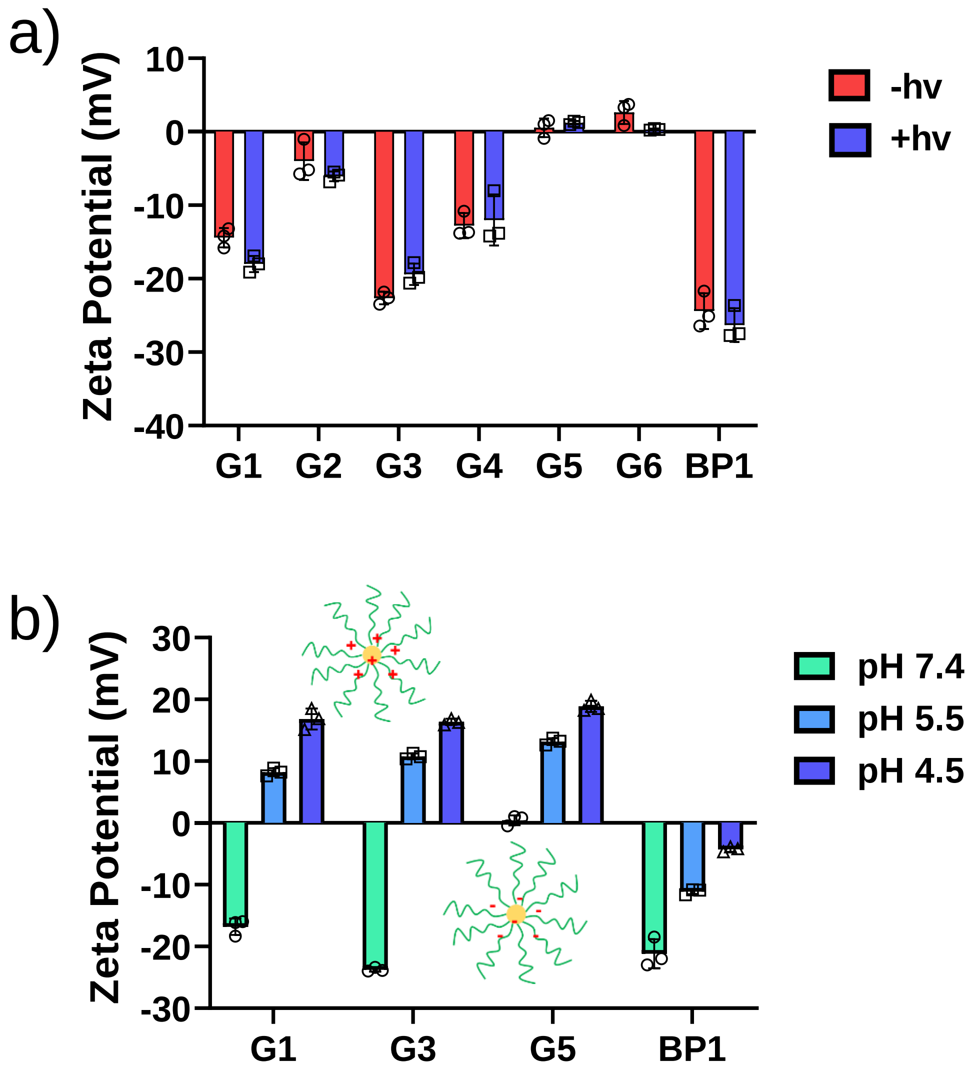

Supplementary Fig. 30. (a) ζ potentials of aqueous dispersions of **G1**-**G6** and **BP1** micelles with or without 630 nm light irradiation for 30 min at pH 7.4. (b) ζ potentials of **G1**, **G3**, **G5**, and **BP1** micelles at different pH values (7.4, 5.5, and 4.5) (n = 3 independent samples).

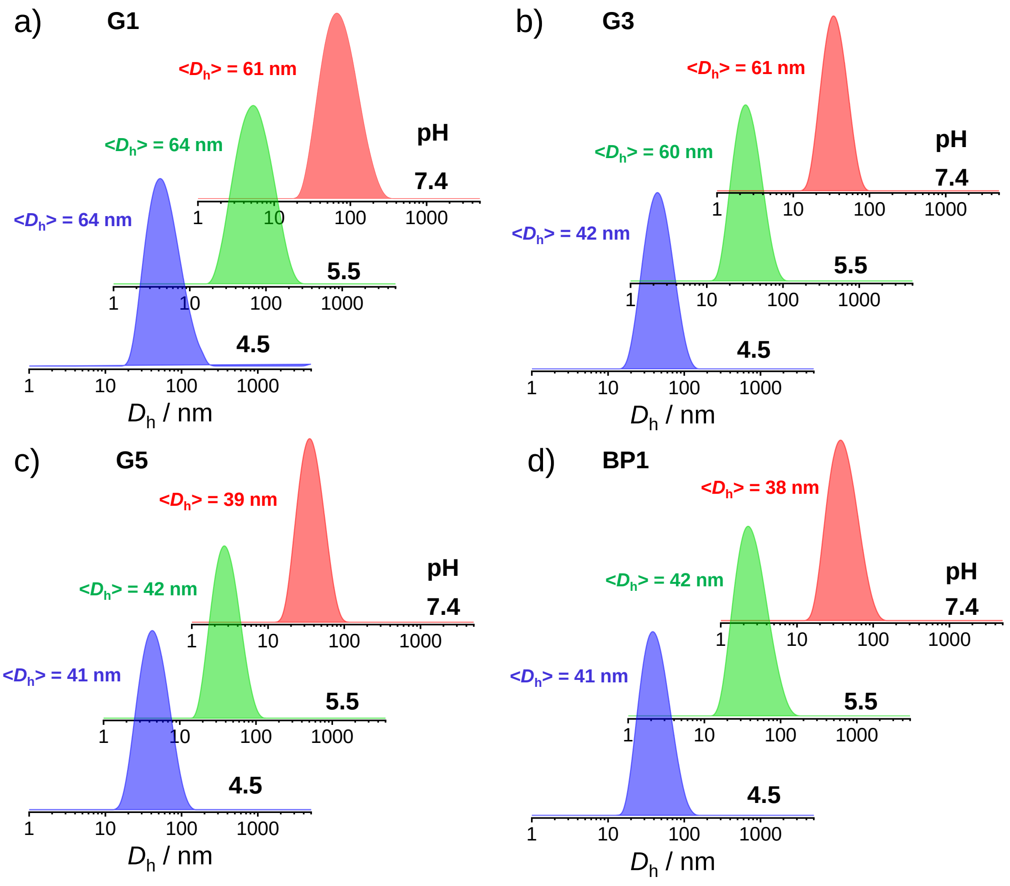

Supplementary Fig. 31. Intensity-average hydrodynamic diameter distributions of aqueous dispersions (0.2 g/L) of (a) **G1**, (b) **G3**, (c) **G5**, and (d) **BP1** micelles at varying pH values.

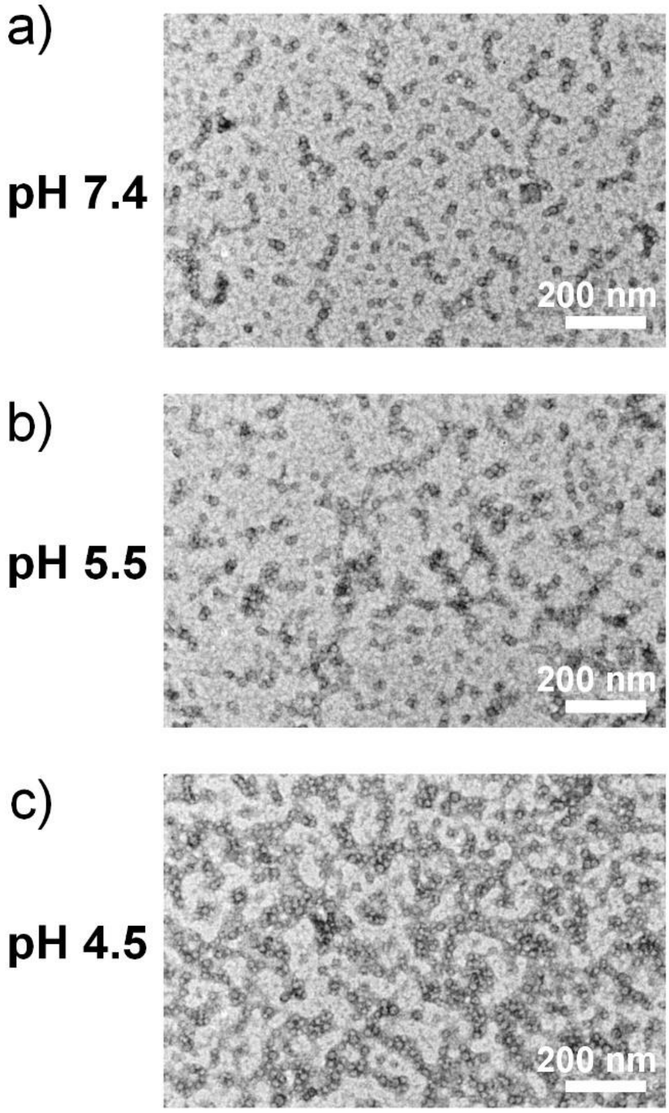

Supplementary Fig. **32**. TEM images of **G1** micelles at pH (a) 7.4, (b) 5.5, and (c) 4.5.

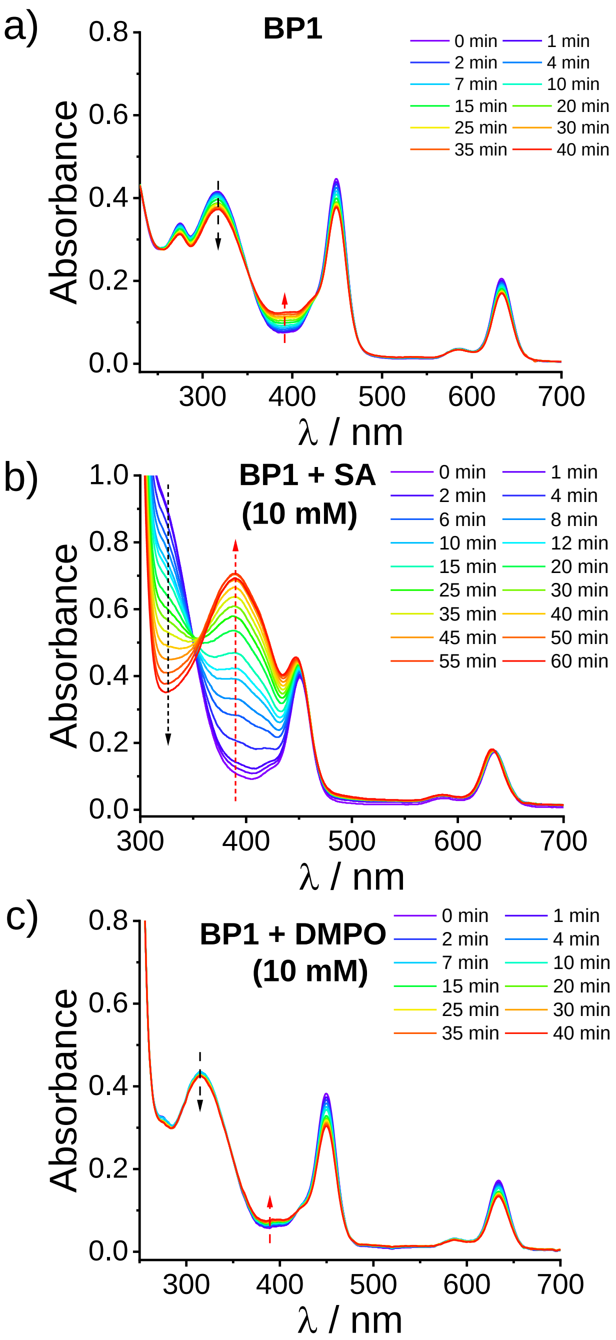

Supplementary Fig. **33**. (a) Evolution of UV-vis absorbance spectra of **BP1** micelles under 630 nm (39 mW/cm^2) light irradiation. (b, c) Evolution of UV-vis absorbance spectra of **BP1** micelles in the presence of (b) sodium ascorbate (10 mM) and (c) DMPO (10 mM) under 630 nm light irradiation.

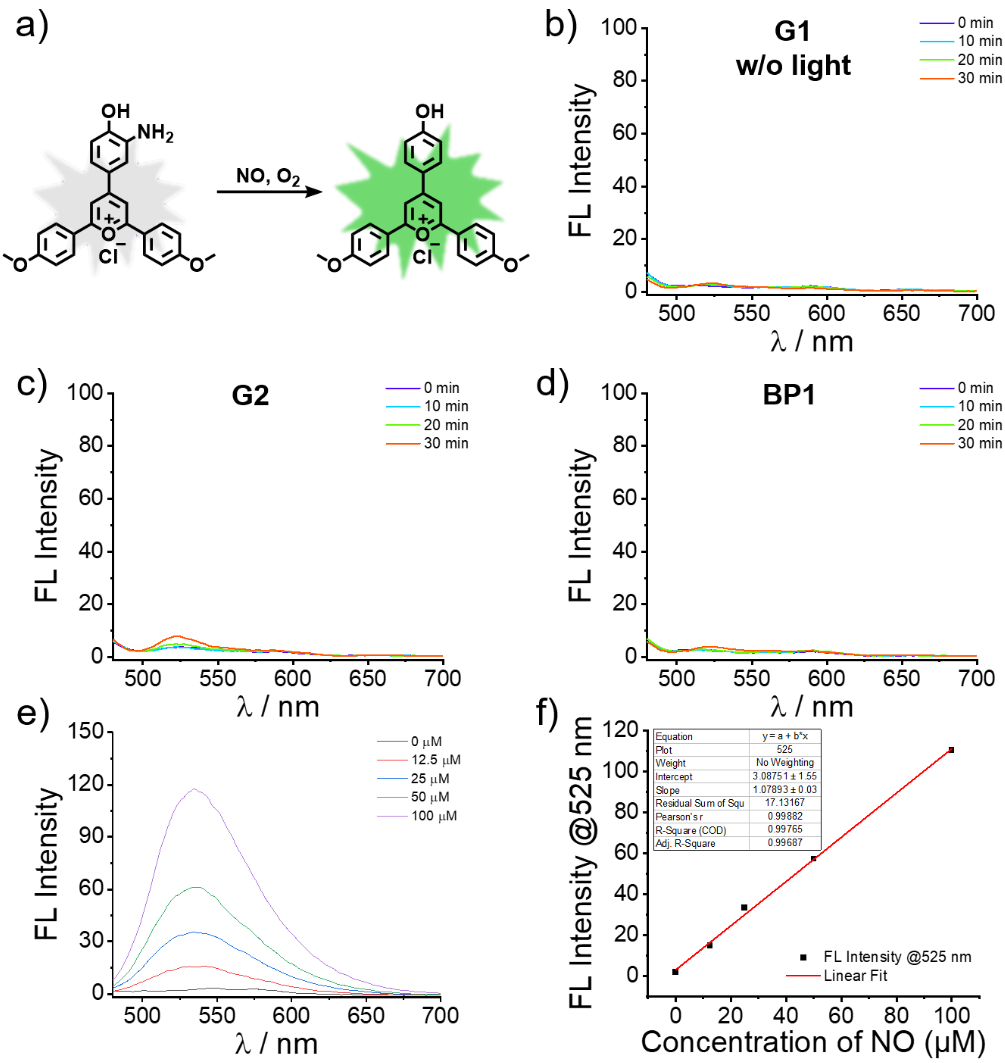

Supplementary Fig. 34. (a) The proposed working mechanism of NOFP. Evolution of fluorescence emission spectra of pyrylium cation-based NO probe (NOFP) (50 μM) under varying conditions: (b) **G1** micelles without light irradiation, (c) **G2** micelles under 630 nm light irradiation, and (d) **BP1** micelles under 630 nm light irradiation. (e) Emission spectra of NOFP in the presence of varying amounts of NO. (f) Fluorescence intensity at 525 nm as a function of NO concentrations.

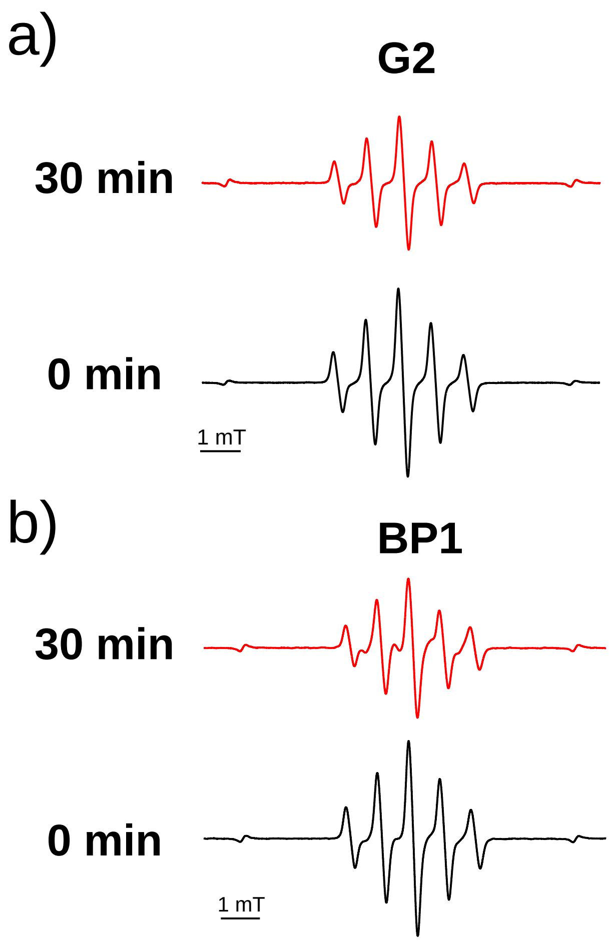

Supplementary Fig. 35. EPR spectra of (a) **G2** and (b) **BP1** micelles with or without 630 nm irradiation for 30 min.

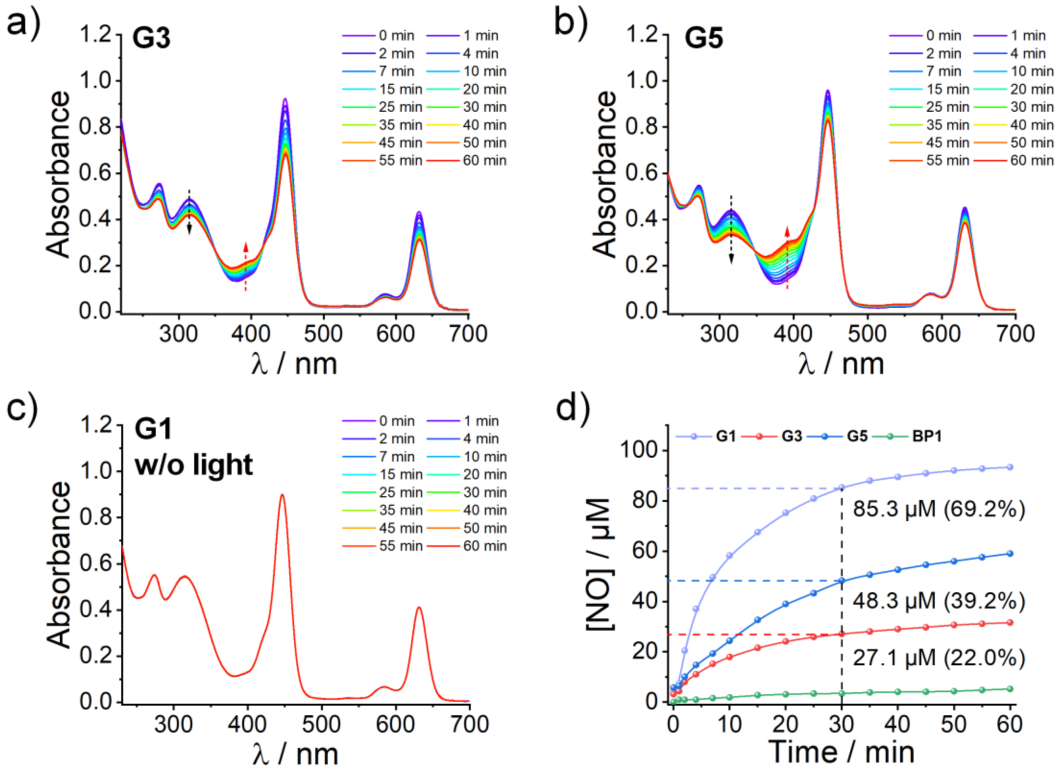

Supplementary Fig. **36**. Evolution of UV-vis absorbance spectra of (a) **G3** and (b) **G5** micelles in PBS (pH 7.4, 10 mM) under 630 nm light irradiation. (c) **G1** micelles in PBS (pH 7.4, 10 mM) under dark conditions. (d) NO release profiles of **G1**, **G3**, **G5**, and **BP1** micelles under 630 nm light irradiation.

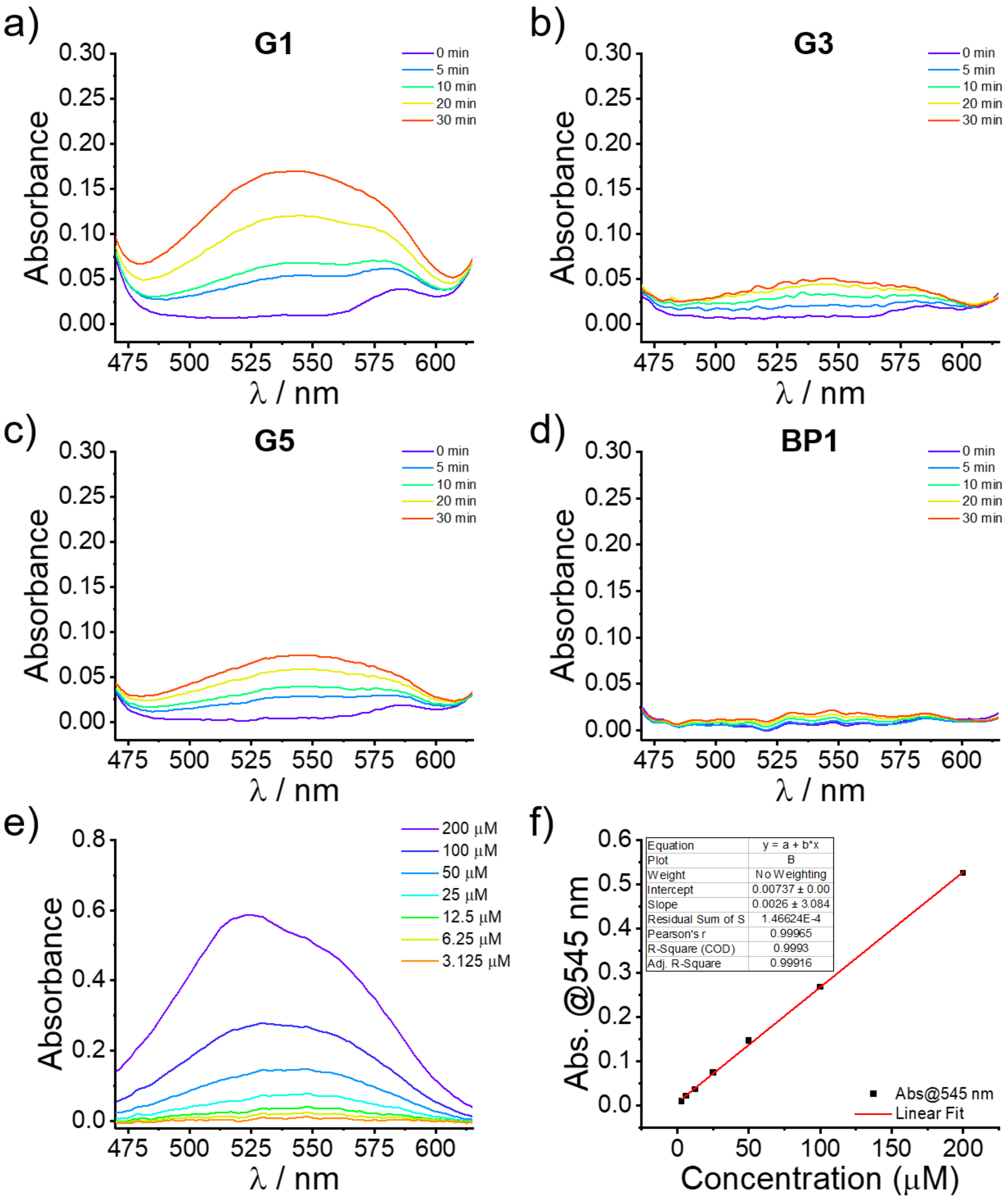

Supplementary Fig. 37. Quantification of nitrite contents using the standard Griess assay by measuring UV-vis absorbance spectra (a) **G1**, (b) **G3**, (c) **G5**, and (d) **BP1** micelles in the presence of Griess reagent under 630 nm irradiation. (e) UV-vis absorbance spectra of Griess reagent in the presence of varying amounts of nitrite. (f) Absorbance intensity at 545 nm as a function of nitrite concentrations.

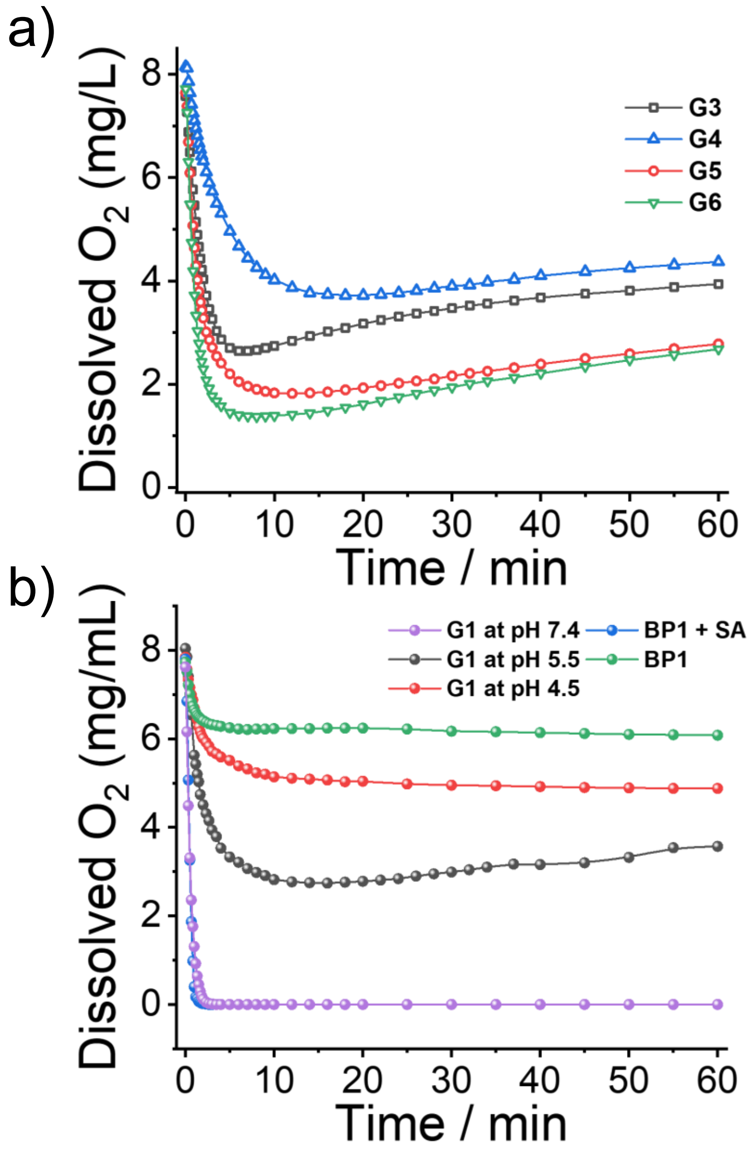

Supplementary Fig. 38. (a) Changes in dissolved oxygen of aqueous dispersions of **G3-G6** micelles (0.2 g/L) in PBS (pH 7.4, 10 mM) under 630 nm light irradiation. (b) Changes in dissolved oxygen of aqueous dispersions of **G1** micelles (0.2 g/L) at pH 7.5, 5.5, 4.5, and **BP1** micelles in the presence or absence of sodium ascorbate (SA, 10 mM) under 630 nm light irradiation (39 mW/cm^2). Note that all tests were performed under open air conditions.

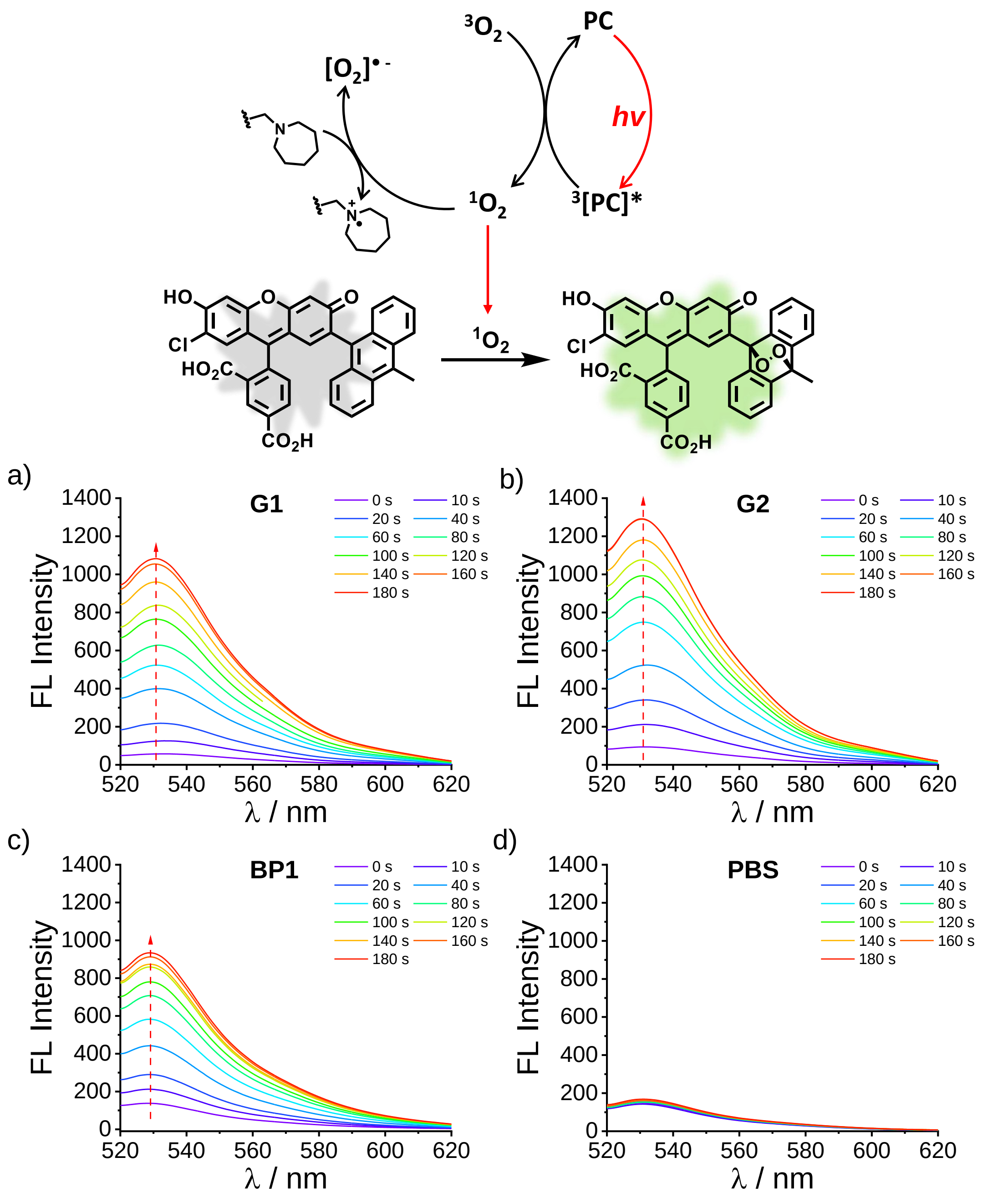

Supplementary Fig. **39**. Fluorescence emission spectra (λ_{ex} = 504 nm; slit width: Ex. 5 nm, Em. 5 nm) of aqueous dispersions of (a) **G1**, (b) **G2**, (c) **BP1** micelles, and (d) PBS in the presence of SOSG (5 µM) under 630 nm irradiation (39 mW/cm^2).

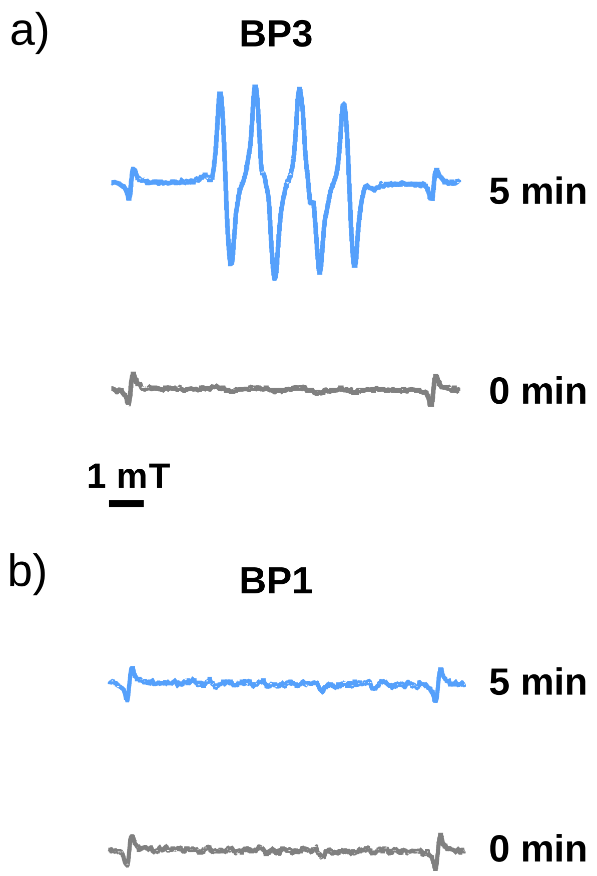

Supplementary Fig. 40. EPR spectra of (a) **BP3** and (b) **BP1** in DMF (0.1 g/L) with or without 630 nm irradiation for 5 min. DMPO (5 mM) was employed as a trapping agent for superoxide anions.

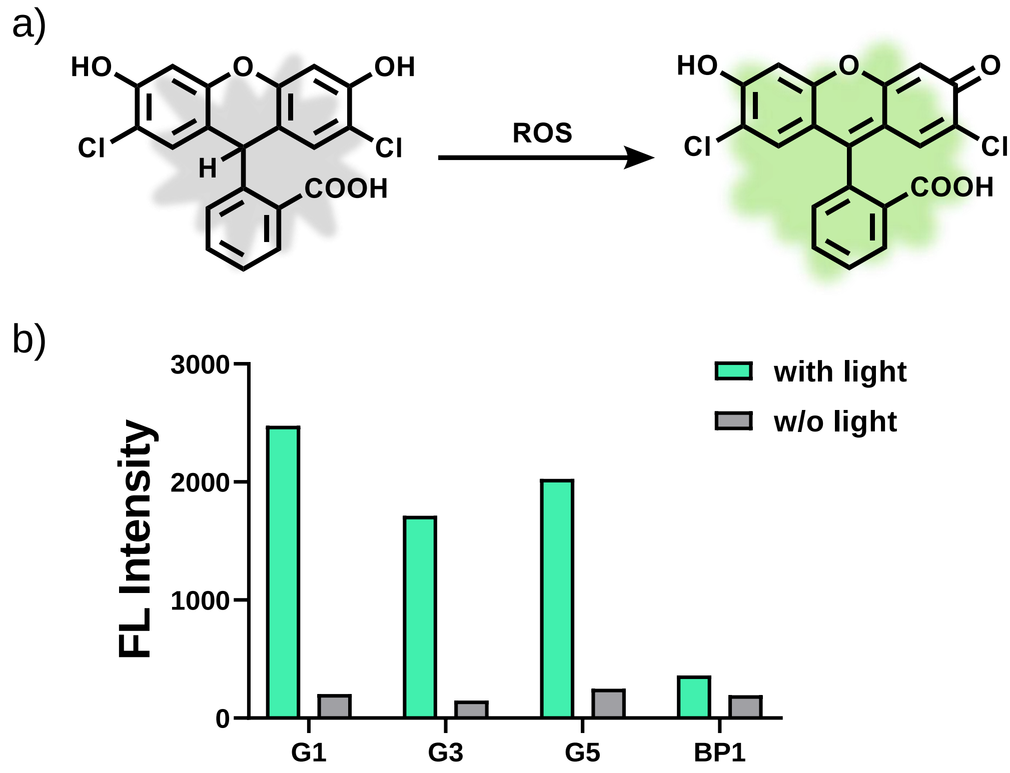

Supplementary Fig. 41. (a) The proposed working mechanism of DCFH. (b) Fluorescence emission spectra (λ_{ex} = 488 nm; slit width: Ex. 5 nm, Em. 5 nm) of aqueous dispersions of **G1**, **G3**, **G5**, and **BP1** micelles in the presence of **DCFH** (100 μM) with or without 630 nm irradiation (39 mW/cm^2) for 10 min.

Supplementary Notes. To minimize the effects of short-lived reactive oxygen species such as singlet oxygen and superoxide anions, micellar nanoparticles were first irradiated under 630 nm for 10 min. Afterward, the **DCFH** probe was added and the fluorescence emission spectra were recorded. The increased fluorescence was likely attributed to the formation of long-lived ROS, such as hydrogen peroxide.

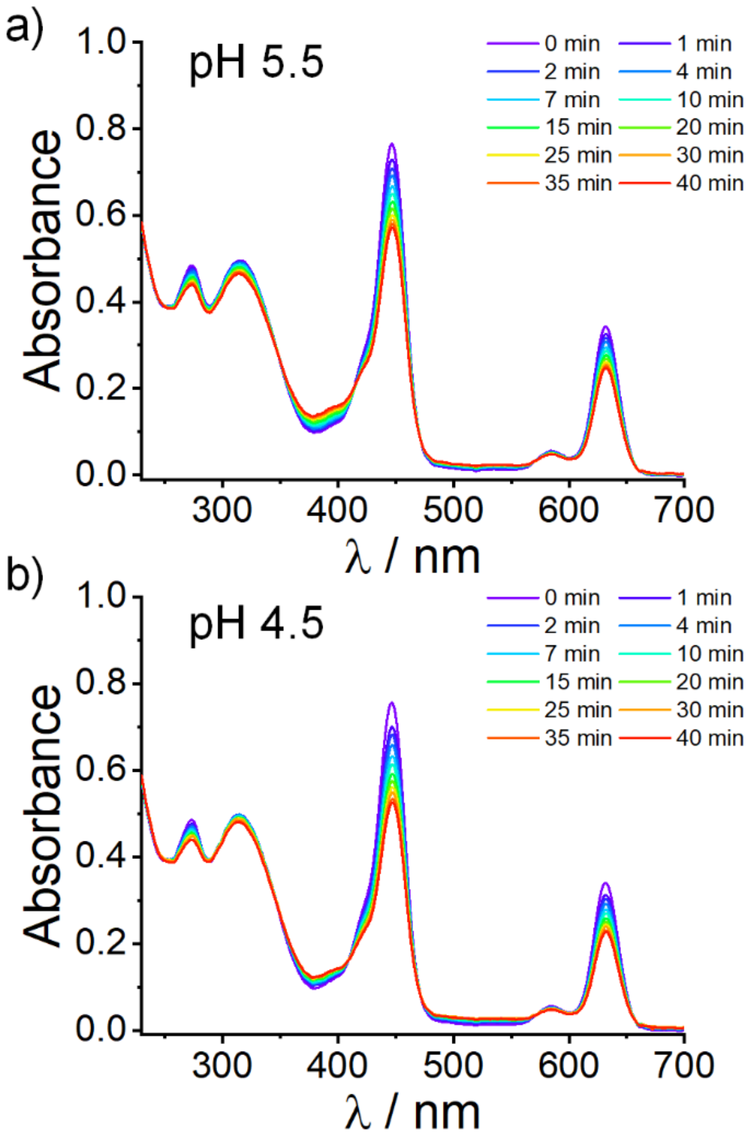

Supplementary Fig. **42**. Evolution of UV-vis absorbance spectra of **G1** micelles at pH (a) 5.5 and (b) 4.5 under 630 nm light irradiation (39 mW/cm^2).

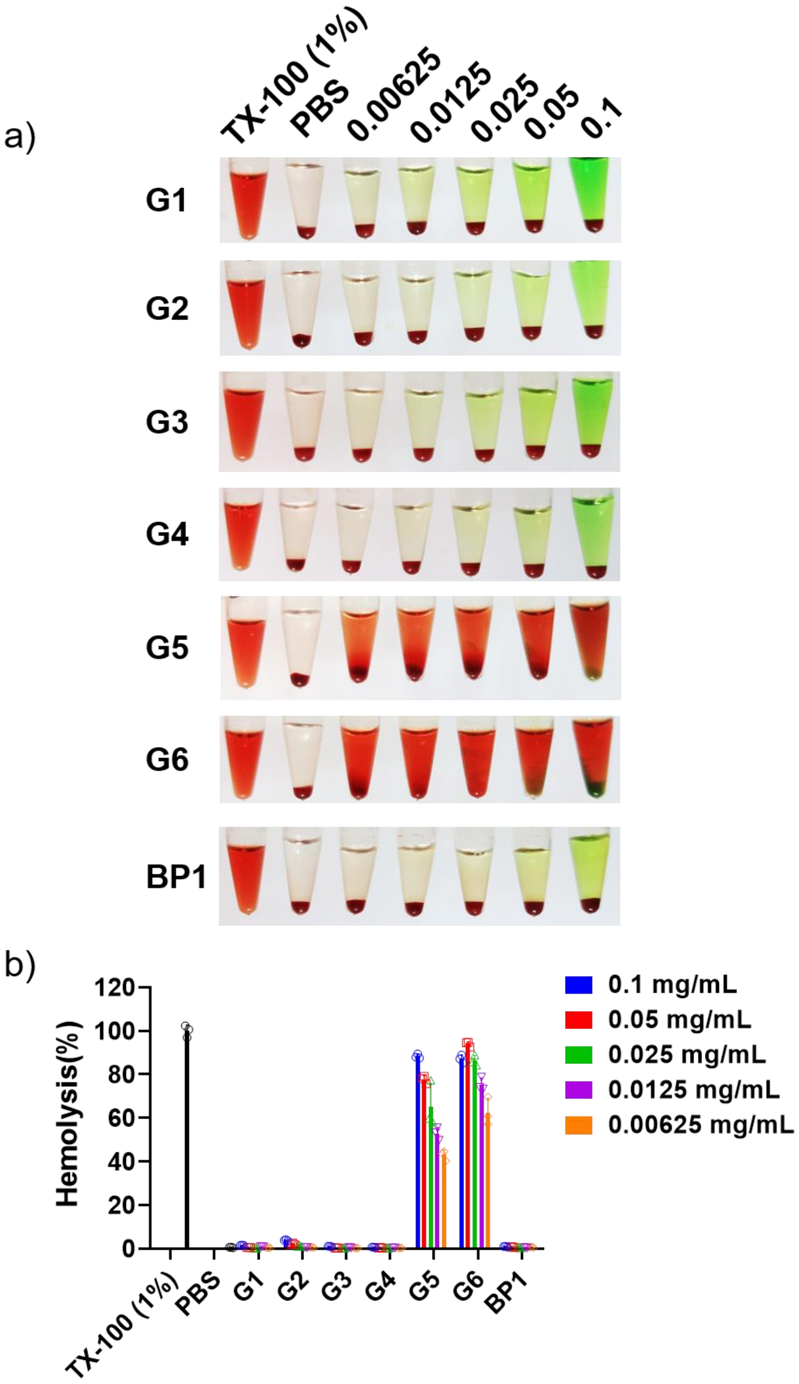

Supplementary Fig. 43. (a) Representative images of sheep red blood cells in the presence of Triton X-100 (1%), PBS, **G1**-**G6**, and **BP1** micelles. (b) Hemolytic assay of **G1**-**G6** and **BP1** micelles, respectively. Triton X-100 (1%) and PBS were used as the positive and negative control, respectively. Data are presented as the mean ± s.d. (n = 3 independent samples).

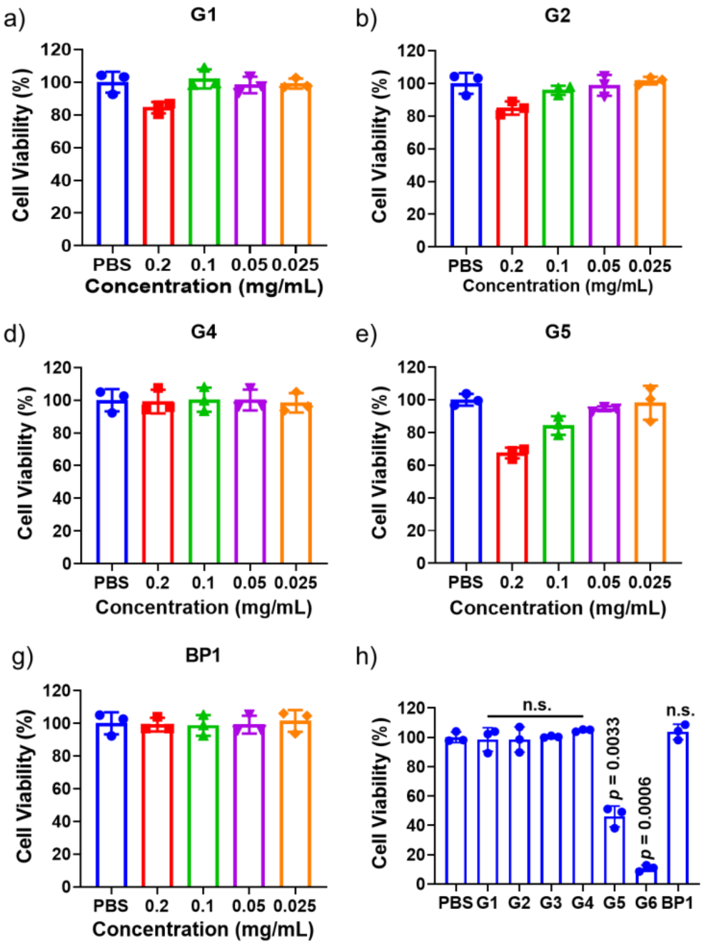

Supplementary Fig. **44**. (a-g) Cell viability of L929 cells as determined by MTT assay of aqueous dispersions of **G1**-**G6** and **BP1** micelles. (h) Cell viability of RAW264.7 cells as determined by MTT assay of aqueous dispersions of **G1**-**G6** and **BP1** micelles (0.1 mg/mL). Data are presented as the mean ± s.d. (n = 3 independent samples). n.s., not significant. Statistical analysis was calculated by two-tailed Student's t-test. Source data are provided as a Source Data file.

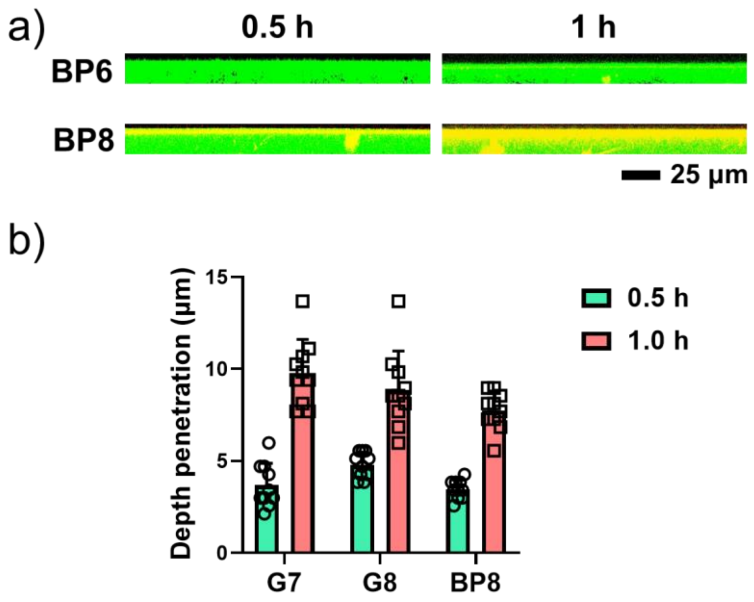

Supplementary Fig. **45**. (a) Representative 3D projection of image z-stacks showing the distribution of **BP6** and **BP8** micelles (red) in CRPA biofilms (Green). (b) Depth penetration in CRPA biofilms after treatment with **G7**, **G8**, and **BP8** micelles for 0.5 and 1 h. Data are presented as mean values ± SD (n = 10 independent locations).

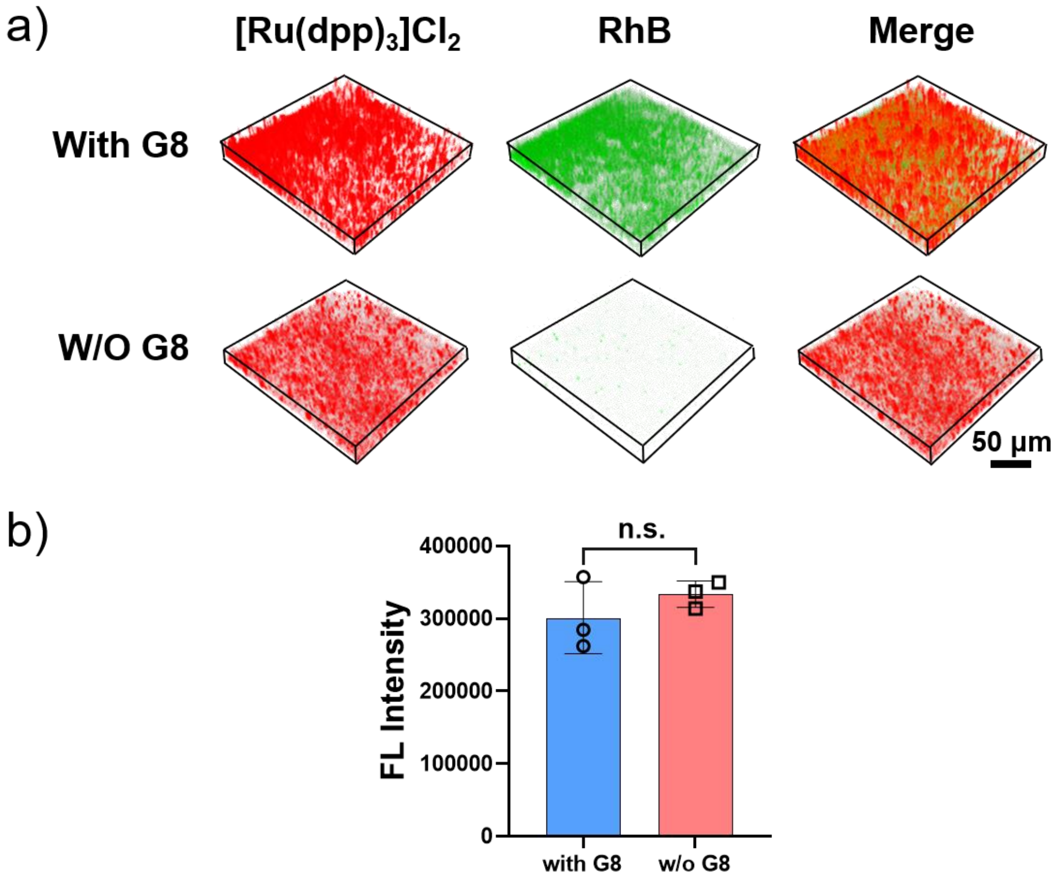

Supplementary Fig. **46**. (a) 3D CLSM images of CRPA biofilms stained with an O_2-specific probe (Ru(dpp)$_3$)Cl$_2$. The biofilm was treated with or without **G8** micelles (0.2 g/L). (b) Quantitative analysis of fluorescence intensities of the red channel. Data are presented as mean values ± SD (n = 3 independent samples). n.s., not significant.

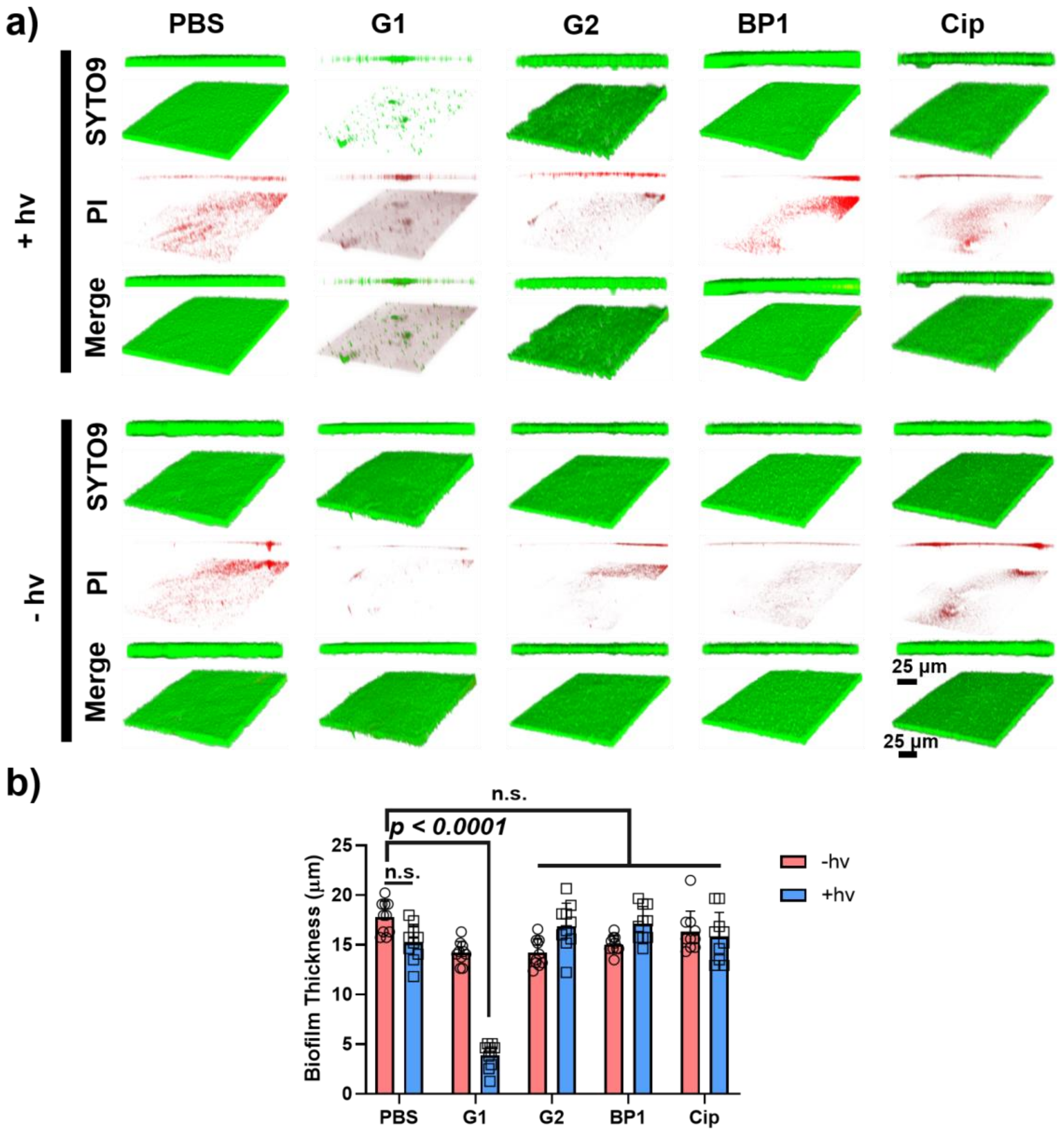

Supplementary Fig. **47**. (a) 3D Confocal microscopy images of Live/Dead staining of CRPA biofilms treated with **G1**, **G2**, **BP1** micelles (0.2 g/L), and Cip (25 μg/mL), with or without 630 nm light irradiation for 30 min (39 mW/cm^2). Green: live bacteria, Red: dead bacteria. (b) Quantification of biofilm thickness receiving various treatments. Data are presented as mean values ± SD (n = 10 independent locations). n.s., not significant. Statistical analysis was calculated by two-tailed Student's *t*-test. Source data are provided as a Source Data file.

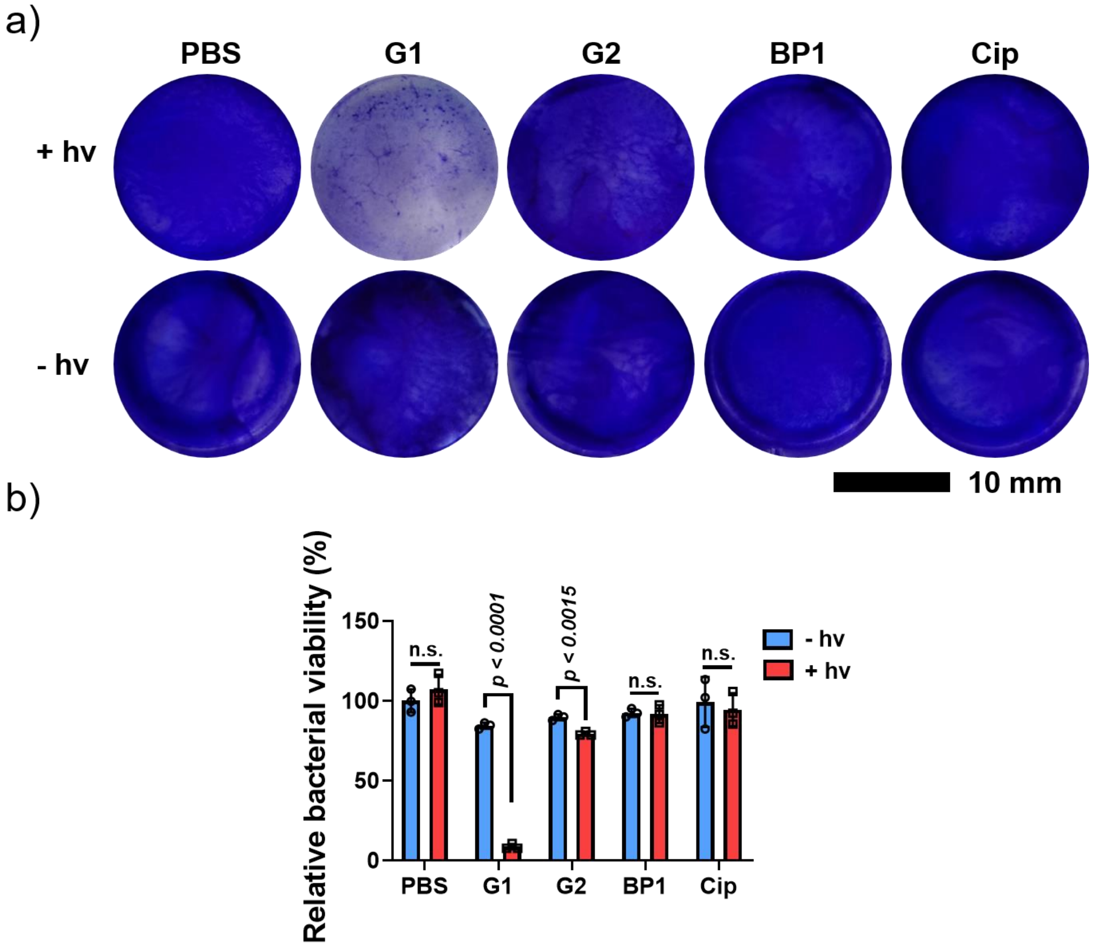

Supplementary Fig. 48. (a) Photographs of CRPA biofilms after treatment with **G1**, **G2**, **BP1** micelles, and Cip (25 µg/mL) with or without 630 nm light irradiation for 30 min. (b) Corresponding bacterial viability after treatment with **G1**, **G2**, **BP1** micelles, and Cip (25 µg/mL) with or without 630 nm light irradiation for 30 min. Data are presented as mean values ± SD (n = 3 independent samples). n.s., not significant. Statistical analysis was calculated by two-tailed Student's *t*-test. Source data are provided as a Source Data file.

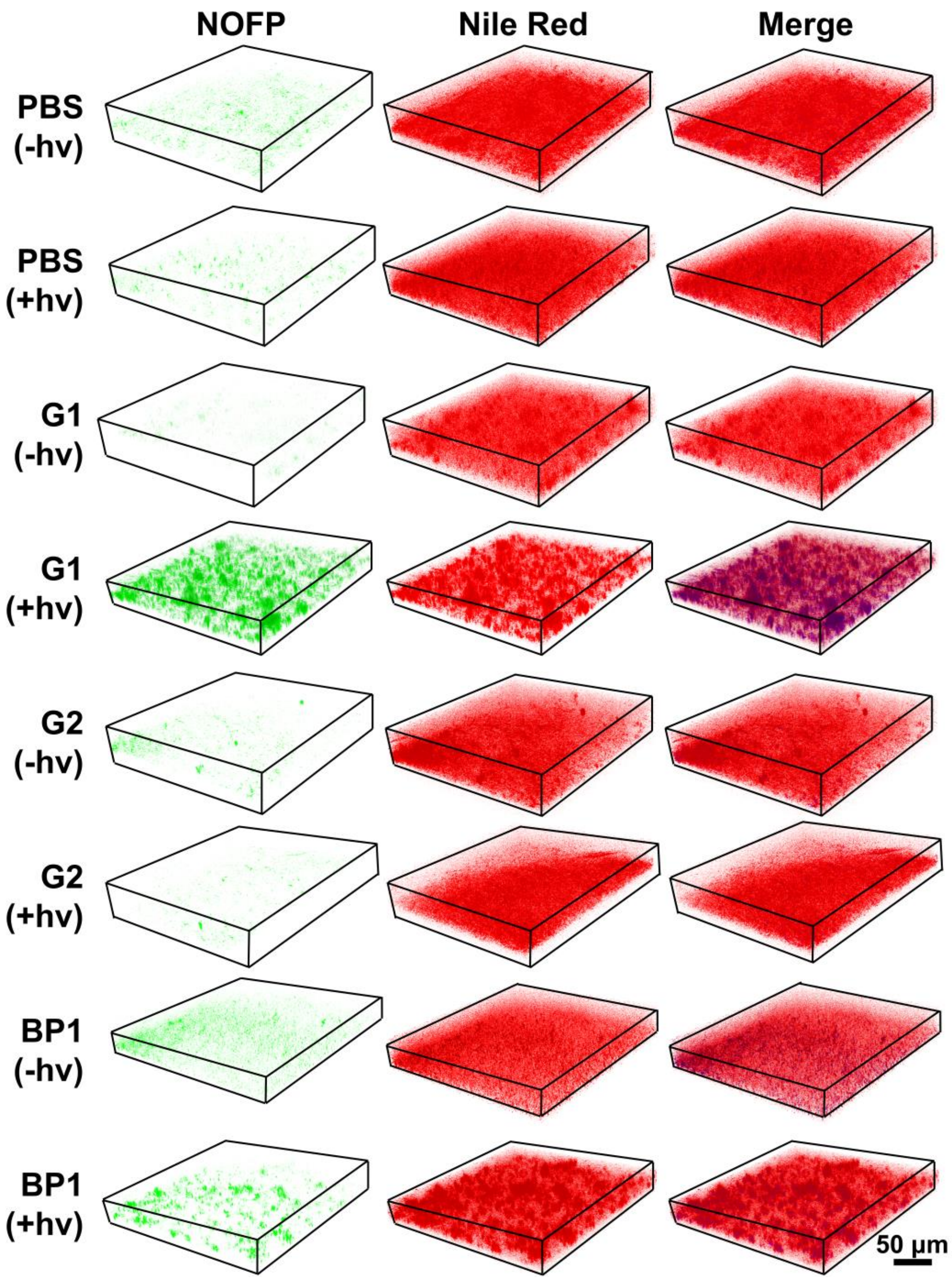

Supplementary Fig. 49. CLSM images of NO release in CRPA biofilms after different treatments. The green channel (NOFP) was excited at 488 nm and collected at 500-550 nm and the red channel (Nile red) was excited at 543 nm and collected at 600-650 nm.

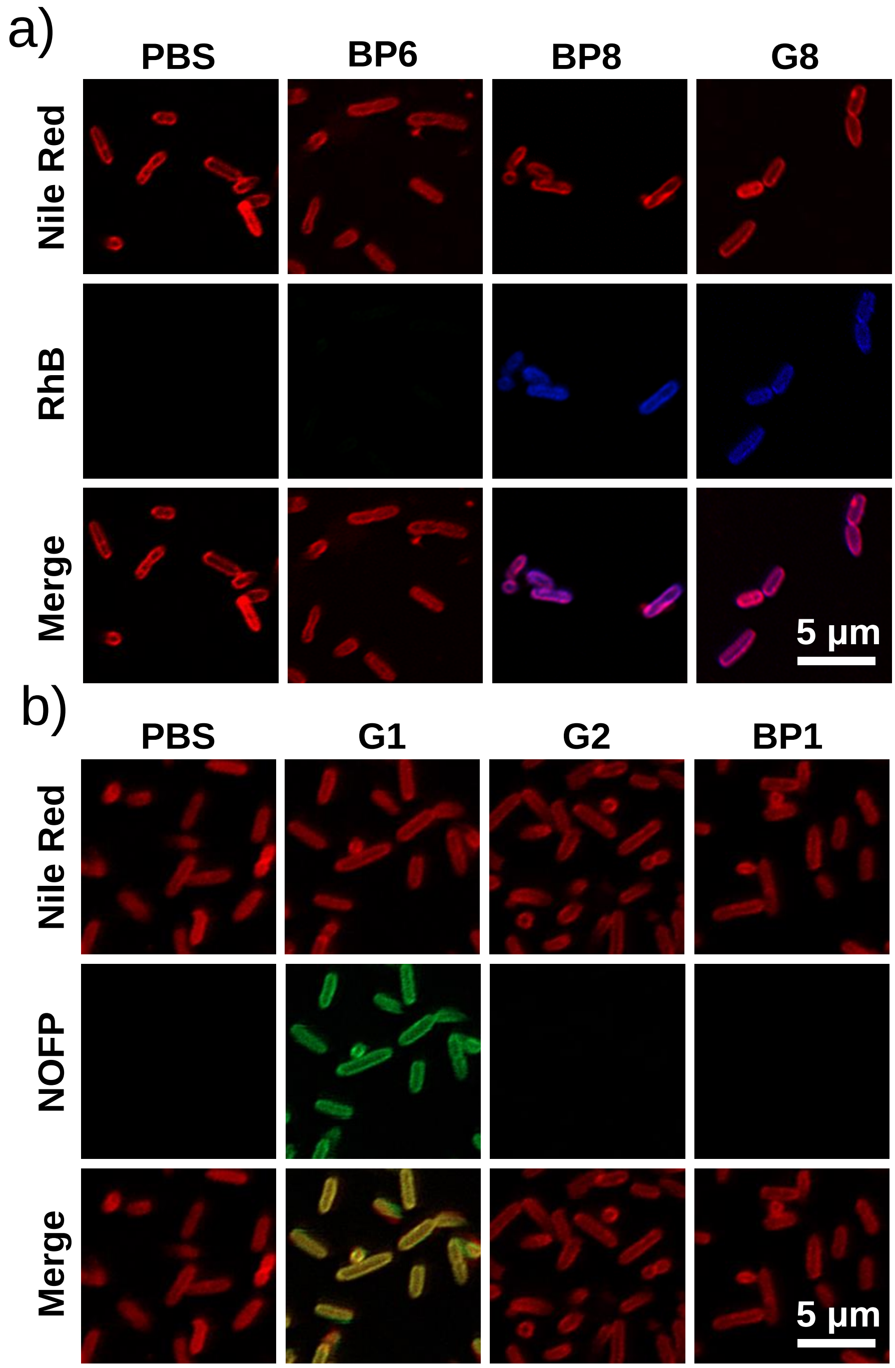

Supplementary Fig. 50. (a) CLSM images of CRPA bacteria incubated with PBS, **BP6**, **BP8**, and **G8** micelles. (b) CLSM images of NO release in CRPA bacteria after different treatments. The Nile red channel was excited at 543 nm and collected at 600-650 nm, the RhB channel was excited at 543 nm and collected at 560-590 nm, and the NOFP channel was excited at 488 nm and collected at 500-550 nm.

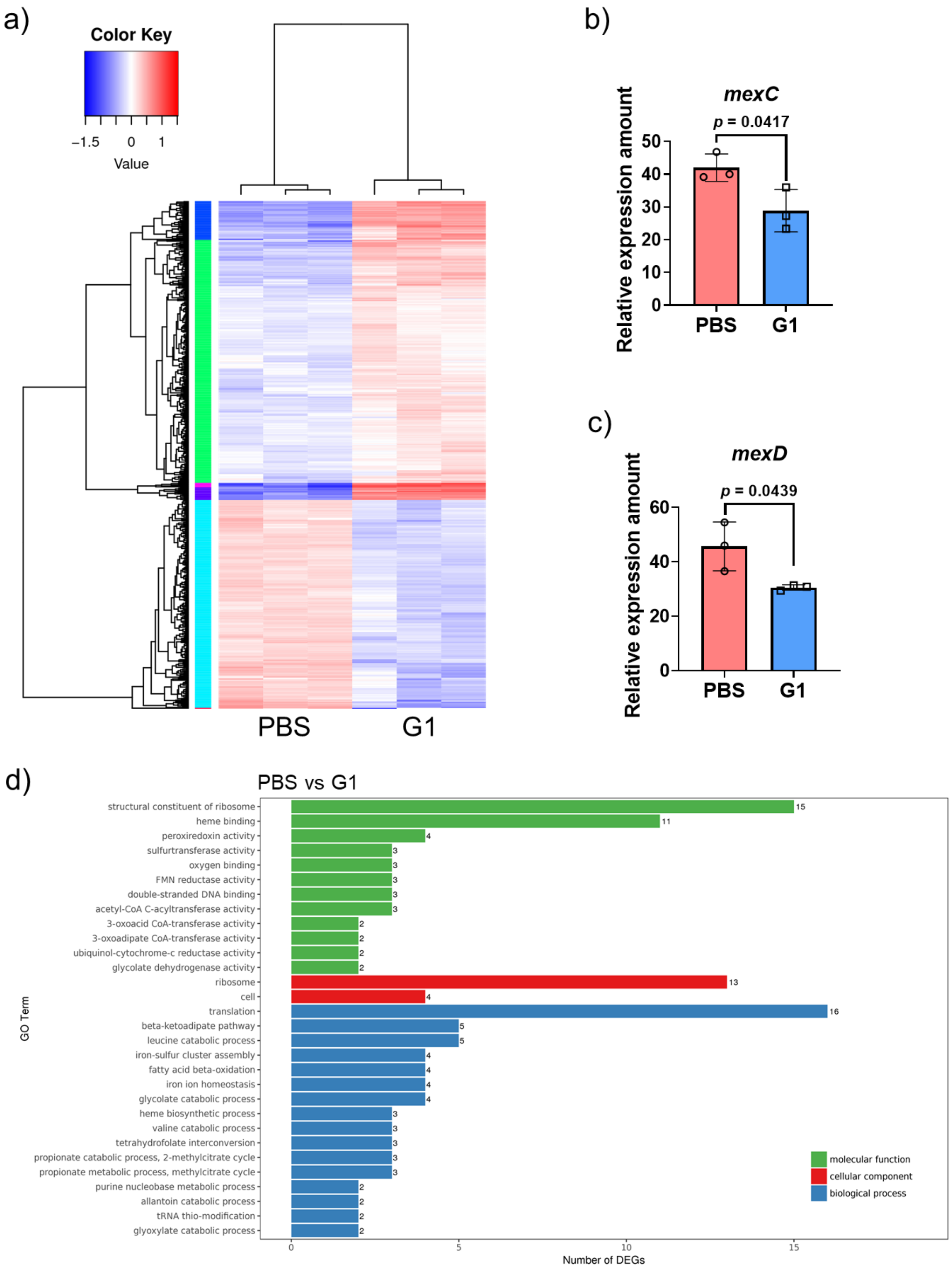

Supplementary Fig. 51. (a) Heat map of differentially expressed genes among CRPA bacteria treated with PBS and **G1** micelles. The relative expression amounts of (b) *mexC*, and (c) *mexD* in CRPA bacteria treated with PBS and **G1** micelles with 630 nm light irradiation for 30 min. (d) GO enrichment histogram. Data are presented as mean values ± SD (n = 3 independent samples). Statistical analysis was calculated by two-tailed Student's *t*-test.

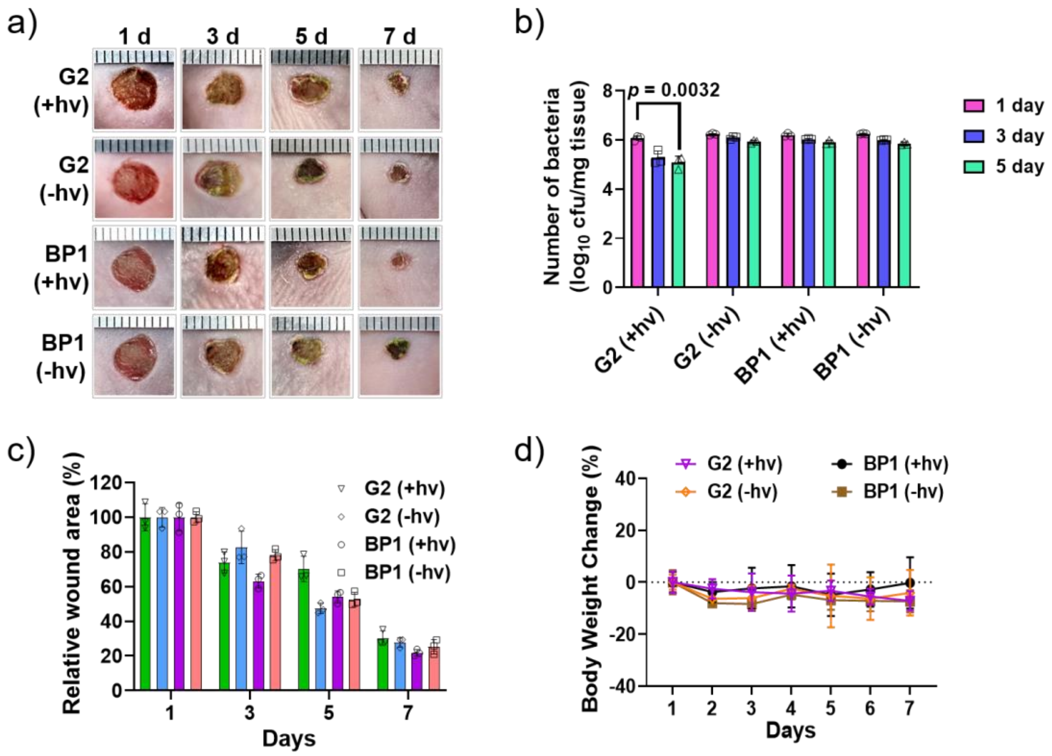

Supplementary Fig. 52. (a) Representative skin wound images at 1, 3, 5, and 7 days and (b) bacterial colony-forming units separated from wound tissues with varying treatments. (c) Quantitative analysis of the residual wounded areas receiving different treatments. (d) Changes in body weights of CRPA-infected mice after different treatments. Data are presented as mean values ± SD (n = 3 independent samples). Statistical analysis was calculated by two-tailed Student's *t*-test.

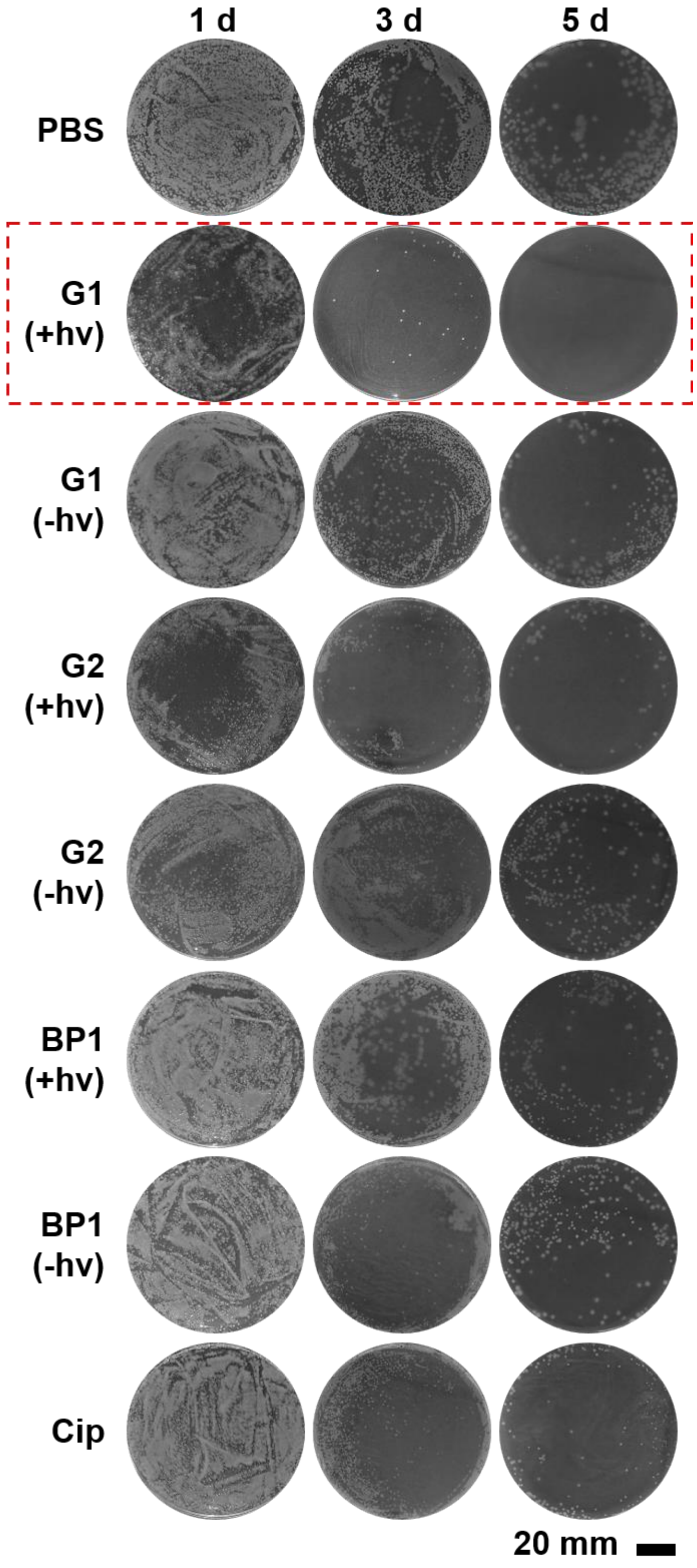

Supplementary Fig. **53**. Images of CRPA colonies growing on agar plates derived from the homogenized infected tissues after various treatments at 1, 3, and 5 days.

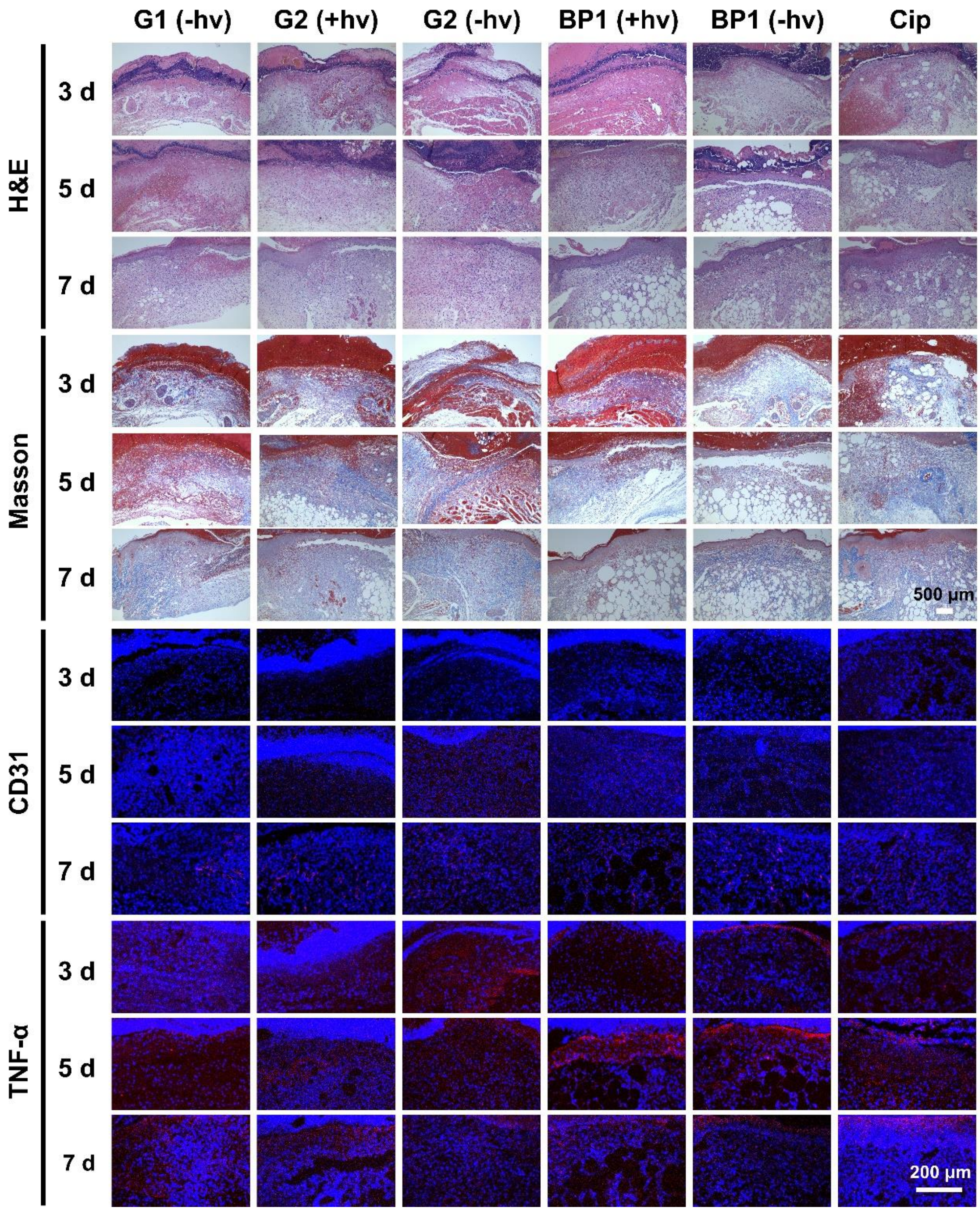

Supplementary Fig. 54. Histological and immunohistological analysis of the CRPA-infected tissues after various treatments.

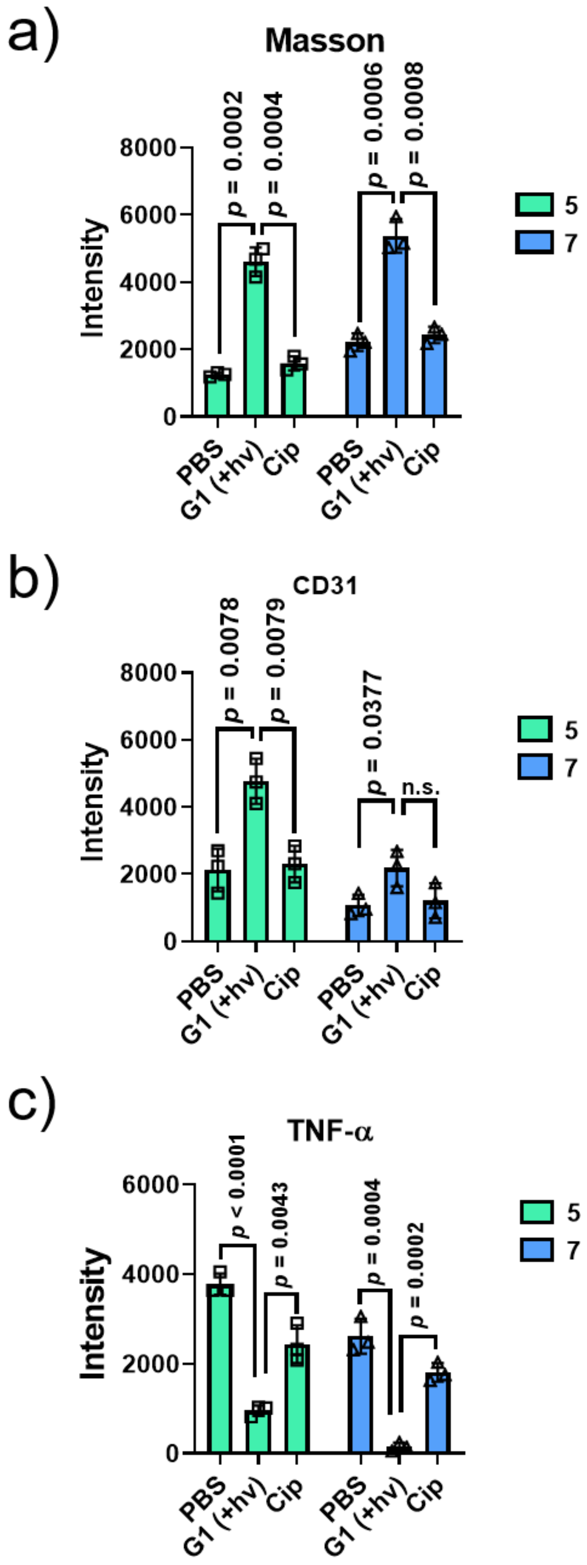

Supplementary Fig. 55. Quantitative comparison of the relative intensities of collagen, CD31, and TNF-α on days 5 and 7. Data are presented as mean values ± SD (n = 3 independent samples). n.s., not significant. Statistical analysis was calculated by two-tailed Student's *t*-test.

Supplementary Table 1. Summary of the apparent photocatalysis rate constants (k_{obs}) of NBNO in the presence of different concentrations of PdTPTBP from Supplementary Fig. 12b.

**Concentration (μM)**	**0.2**	**0.5**	**1.0**	**5.0**	**10.0**
k_{obs} **(min^{-1})**	0.034	0.055	0.086	0.202	0.885
R^2	0.999	0.999	0.999	0.997	0.997

Supplementary Table 2. Structural parameters of diblock copolymers used in this study.

**Entry**	**Chemical Composition**[a]	$M_{n,\ NMR}$[b]	$M_{n,\ SEC}$[c]	M_w/M_n[c]	pK_a[d]
**BP1**	PEG_{113}-*b*-P($NBNO_{0.88}$-*co*-$PdM_{0.12}$)$_{21}$	15.0	7.0	1.06	/
**BP2**	PEG_{113}-*b*-P($NBNH_{0.86}$-*co*-$PdM_{0.14}$)$_{21}$	14.8	6.4	1.10	/
**BP3**	PEG_{113}-*b*-P($C7A_{0.95}$-*co*-$PdM_{0.05}$)$_{47}$	18.4	8.7	1.10	6.27
**BP4**	PEG_{113}-*b*-P($DPA_{0.95}$-*co*-$PdM_{0.05}$)$_{53}$	19.0	9.7	1.07	5.96
**BP5**	PEG_{113}-*b*-P($DEA_{0.95}$-*co*-$PdM_{0.05}$)$_{53}$	18.7	9.1	1.08	6.98
**BP6**	PEG_{113}-*b*-P($NBNO_{0.84}$-*co*-$RhB_{0.16}$)$_{32}$	18.0	8.5	1.06	/
**BP7**	PEG_{113}-*b*-$PC7A_{51}$	16.1	8.0	1.08	/
**BP8**	PEG_{113}-*b*-P($C7A_{0.90}$-*co*-$RhB_{0.10}$)$_{48}$	14.7	6.4	1.06	/

[a] Chemical compositions of block copolymers determined by ^{1}H NMR. [b] Molecular

weights calculated based on NMR results. [c] Number-average molecular weight, $M_{n, SEC}$, and molecular weight distribution, M_w/M_n, determined by SEC using THF as the eluent. [d] pK_a calculated from titration curves.

Supplementary Table 3. Summary of micellar nanoparticles co-assembled from varying diblock polymers.

Entry	Chemical Composition
**G1**	**BP1/BP3**
**G2**	**BP2/BP3**
**G3**	**BP1/BP4**
**G4**	**BP2/BP4**
**G5**	**BP1/BP5**
**G6**	**BP2/PB5**
**G7**	**BP6/BP7**
**G8**	**BP6/BP8**

Supplementary References

[1] A. Beltran, M. I. Burguete, D. R. Abanades, D. Perez-Sala, S. V. Luis, F. Galindo, *Chem. Commun.* **2014**, *50*, 3579-3581.

[2] S. Cui, J. Qiao, M. P. Xiong, *Mol. Pharm.* **2022**, *19*, 2406-2417.

[3] X. Cui, J. Zhao, P. Yang, J. Sun, *Chem. Commun.* **2013**, *49*, 10221-10223.

[4] K. Zhou, Y. Wang, X. Huang, K. Luby-Phelps, B. D. Sumer, J. Gao, *Angew. Chem. Int. Edit.* **2011**, *50*, 6109-6114.

[5] J. Rieger, W. J. Zhang, F. Stoffelbach, B. Charleux, *Macromolecules* **2010**, *43*, 6302-6310.

[6] W. Xiu, L. Wan, K. Yang, X. Li, L. Yuwen, H. Dong, Y. Mou, D. Yang, L. Wang, *Nat. Commun.* **2022**, *13*, 3875.
