# Supplementary material for: Biofilm heterogeneity-adaptive photoredox catalysis enables red light-triggered nitric oxide release for combating drug-resistant infections
Source: Nat Commun. 2023 Nov 18;14:7510. doi: 10.1038/s41467-023-43415-8 (PMC10657346; doi:10.1038/s41467-023-43415-8)
Supplement: Supplementary file 3 — Description of Additional Supplementary Files [file 41467_2023_43415_MOESM3_ESM.pdf]

Description of Additional Supplementary Files

File Name: Supplementary Data 1
Description: Atomic cartesian coordinates of intermediates and transition states in the most favored path (Å).
