# Supplementary material for: Biofilm heterogeneity-adaptive photoredox catalysis enables red light-triggered nitric oxide release for combating drug-resistant infections
Source: Nat Commun. 2023 Nov 18;14:7510. doi: 10.1038/s41467-023-43415-8 (PMC10657346; doi:10.1038/s41467-023-43415-8)
Supplement: Supplementary file 5 — Reporting Summary [file 41467_2023_43415_MOESM5_ESM.pdf]

nature portfolio

Corresponding author(s): Jinming Hu, Shiyong Liu, Chen Zhu

Last updated by author(s): Nov 9, 2023

Reporting Summary

Nature Portfolio wishes to improve the reproducibility of the work that we publish. This form provides structure for consistency and transparency in reporting. For further information on Nature Portfolio policies, see our Editorial Policies and the Editorial Policy Checklist.

Statistics

For all statistical analyses, confirm that the following items are present in the figure legend, table legend, main text, or Methods section.

n/a	Confirmed	
☐	☒	The exact sample size (n) for each experimental group/condition, given as a discrete number and unit of measurement
☐	☒	A statement on whether measurements were taken from distinct samples or whether the same sample was measured repeatedly
☐	☒	The statistical test(s) used AND whether they are one- or two-sided<br>*Only common tests should be described solely by name; describe more complex techniques in the Methods section.*
☒	☐	A description of all covariates tested
☒	☐	A description of any assumptions or corrections, such as tests of normality and adjustment for multiple comparisons
☐	☒	A full description of the statistical parameters including central tendency (e.g. means) or other basic estimates (e.g. regression coefficient) AND variation (e.g. standard deviation) or associated estimates of uncertainty (e.g. confidence intervals)
☐	☒	For null hypothesis testing, the test statistic (e.g. F, t, r) with confidence intervals, effect sizes, degrees of freedom and P value noted<br>*Give P values as exact values whenever suitable.*
☒	☐	For Bayesian analysis, information on the choice of priors and Markov chain Monte Carlo settings
☒	☐	For hierarchical and complex designs, identification of the appropriate level for tests and full reporting of outcomes
☒	☐	Estimates of effect sizes (e.g. Cohen's d, Pearson's r), indicating how they were calculated

Our web collection on statistics for biologists contains articles on many of the points above.

Software and code

Policy information about availability of computer code

Data collection	1H and 13C NMR spectra were taken on a 400 MHz Bruker nuclear magnetic resonance (NMR) spectrometer. Deuterated chloroform (CDCl3) and dimethylsulfoxide (DMSO-d6) were used as solvents. High-resolution electrospray ionization mass spectrometry (HR-ESI-MS) spectra were performed on Waters XEVO® G2-XS-TOF Mass Spectrometer equipped with an electrospray interface. High-performance liquid chromatography (HPLC) analysis was performed with a Shimadzu HPLC system, equipped with an LC-20AP binary pump, an SPD-20A UV-vis detector, and a Symmetry C18 column. UV-vis absorption spectra were acquired on a UV-3600i Plus UV-vis spectrophotometer (Shimadzu). Molecular weights and molecular weight distributions were determined by size exclusion chromatography (SEC) equipped with Waters 1515 pump and Waters 2414 differential refractive index detector (set at 30 °C). It used a series of two linear Styragel columns (HR2 and HR4) at an oven temperature of 45 °C. The eluent was THF at a flow rate of 1.0 mL/min. Narrowly dispersed polystyrenes were employed as the standards for calibration. Transmission electron microscopy (TEM) was conducted on a JEOL 2010 electron microscope at an acceleration voltage of 200 kV. Scanning electron microscopy (SEM) was conducted on a Zeiss Gemini 500 field emission scanning electron microscope at an acceleration voltage of 3 kV. Dynamic light scattering (DLS) and zeta potential measurements were conducted on a Zetasizer Nano ZS (Malvern). Electron paramagnetic resonance (EPR) spectra were recorded on a JEOL JES FA200 ESR spectrometer (300 K, 9.063 GHz, Xband) at room temperature. It used the following parameters, microwave power: 1 mW; modulation frequency: 100 kHz; and modulation amplitude: 0.35 mT. Hematoxylin and Eosin (H&E), Masson staining, CD31, and TNF-α staining images were obtained on an IX71 fluorescence microscope (Olympus, Japan). Confocal laser scanning microscopy (CLSM) images were acquired using a Leica TCS SP5 microscope.
Data analysis	The wound area and quantitative comparison of the relative intensities of collagen, CD31, and TNF-α was carried out using image J Fiji. Data process was conducted using Origin 2021 and Graph Pad prism 8.0. All the density-functional theory (DFT) calculations were performed at the level of B3LYP/6-31G+(d, p) (LANL2DZ on Pd) in IEFPCM-DMSO solvent using the Gaussian 09 software package.

For manuscripts utilizing custom algorithms or software that are central to the research but not yet described in published literature, software must be made available to editors and reviewers. We strongly encourage code deposition in a community repository (e.g. GitHub). See the Nature Portfolio guidelines for submitting code & software for further information.

Data

Policy information about availability of data

All manuscripts must include a data availability statement. This statement should provide the following information, where applicable:

- Accession codes, unique identifiers, or web links for publicly available datasets
- A description of any restrictions on data availability
- For clinical datasets or third party data, please ensure that the statement adheres to our policy

The RNA-Seq data generated in this study have been deposited in the NCBI Gene Expression Omnibus database under the accession code GSE246965. The experimental data generated in this study are available within the Article and Supplementary Information. Source data are provided with this paper. All other data are available from the corresponding authors upon request.

Research involving human participants, their data, or biological material

Policy information about studies with human participants or human data. See also policy information about sex, gender (identity/presentation), and sexual orientation and race, ethnicity and racism.

Reporting on sex and gender	Not applicable in this study.
Reporting on race, ethnicity, or other socially relevant groupings	Not applicable in this study.
Population characteristics	Not applicable in this study.
Recruitment	Not applicable in this study.
Ethics oversight	Not applicable in this study.

Note that full information on the approval of the study protocol must also be provided in the manuscript.

Field-specific reporting

Please select the one below that is the best fit for your research. If you are not sure, read the appropriate sections before making your selection.

[x] Life sciences [ ] Behavioural & social sciences [ ] Ecological, evolutionary & environmental sciences

For a reference copy of the document with all sections, see nature.com/documents/nr-reporting-summary-flat.pdf

Life sciences study design

All studies must disclose on these points even when the disclosure is negative.

Sample size	The sample size were determined as per the pilot study or previous experimental experience and standard protocols.
Data exclusions	No data were excluded.
Replication	All replication attempts were successful and observed marked expression patterns were consistent with previously known results. Each experiments was carried out at least three independent replications and was described in the figure legend with similar results.
Randomization	BALB/c mice were randomly distributed into each subgroup.
Blinding	The technicians were blinded to tissues group during data collection and analyses.

Reporting for specific materials, systems and methods

We require information from authors about some types of materials, experimental systems and methods used in many studies. Here, indicate whether each material, system or method listed is relevant to your study. If you are not sure if a list item applies to your research, read the appropriate section before selecting a response.

Materials & experimental systems

n/a	Involved in the study
☐	☒ Antibodies
☐	☒ Eukaryotic cell lines
☒	☐ Palaeontology and archaeology
☐	☒ Animals and other organisms
☒	☐ Clinical data
☒	☐ Dual use research of concern
☒	☐ Plants

Methods

n/a	Involved in the study
☒	☐ ChIP-seq
☒	☐ Flow cytometry
☒	☐ MRI-based neuroimaging

Antibodies

Antibodies used	Immunofluorescence staining:<br>Anti-TNF-α Rabbit pAb, GB11188-100, Servicebio Biological, China, used at a dilution of 1:200.<br>Anti-CD31 Rabbit pAb, GB11063-2-100, Servicebio Biological, China, used at a dilution of 1:200.<br>Cy3® Goat Anti-Rabbit, GB21303, Servicebio Biological, China, used at a dilution of 1:300.
Validation	All antibodies were well-recognized in the filed and have their validation statement on their manufactures' website.<br>Anti-TNF-α Rabbit pAb, GB11188-100, Servicebio Biological, China, used at a dilution of 1:200. https://www.servicebio.cn/goodsdetail?id=4760<br>Anti-CD31 Rabbit pAb, GB11063-2-100, Servicebio Biological, China, used at a dilution of 1:200. https://www.servicebio.cn/goodsdetail?id=1345<br>Cy3® Goat Anti-Rabbit, GB21303, Servicebio Biological, China, used at a dilution of 1:300. https://www.servicebio.cn/goodsdetail?id=253

Eukaryotic cell lines

Policy information about cell lines and Sex and Gender in Research

Cell line source(s)	Raw264.7 and L929 cells were purchased from Shanghai Gaining Biological Technologu Co., Ltd.
Authentication	None of the cell lines used in this study were authenticated by the authors.
Mycoplasma contamination	All cell lines were tested negative for mycoplasma contamination.
Commonly misidentified lines (See ICLAC register)	No misidentified cell line was involved in the study.

Animals and other research organisms

Policy information about studies involving animals; ARRIVE guidelines recommended for reporting animal research, and Sex and Gender in Research

Laboratory animals	All BALB/c mice (6-8 weeks, female) were purchased from Experimental Animal Center of Anhui Medical University and all animal experiments were conducted following a protocol approved by institutional Anhui care and Use Committee (University of Science and Technology of China).<br>All animals were maintained on a 12-12 light-dark cycle with a temperature of 25 °C and humidity of 48-52%.
Wild animals	No wild animals were used in the study.
Reporting on sex	BALB/c mice (6-8 weeks, female) were used in this study.
Field-collected samples	The study does not involve field-collected study.
Ethics oversight	All the animal studies described in this research were approved by the Committee on the Ethics of Animal Experiments of the University of Science and Technology of China (USTC) and were performed in strict accordance with the Animal Care and Use Committee of USTC.

Note that full information on the approval of the study protocol must also be provided in the manuscript.
